# Grey and white matter associations of psychotic-like experiences in a general population sample (UK Biobank)

**DOI:** 10.1038/s41398-020-01131-7

**Published:** 2021-01-07

**Authors:** Julie Schoorl, Miruna C. Barbu, Xueyi Shen, Mat R. Harris, Mark J. Adams, Heather C. Whalley, Stephen M. Lawrie

**Affiliations:** Division of Psychiatry, Kennedy Tower, Royal Edinburgh Hospital, Edinburgh, EH10 5HF UK

**Keywords:** Schizophrenia, Predictive markers

## Abstract

There has been a substantial amount of research reporting the neuroanatomical associations of psychotic symptoms in people with schizophrenia. Comparatively little attention has been paid to the neuroimaging correlates of subclinical psychotic symptoms, so-called “psychotic-like experiences” (PLEs), within large healthy populations. PLEs are relatively common in the general population (7–13%), can be distressing and negatively affect health. This study therefore examined gray and white matter associations of four different PLEs (auditory or visual PLEs, and delusional ideas about conspiracies or communications) in subjects of the UK Biobank study with neuroimaging data (*N* = 21,390, mean age = 63 years). We tested for associations between any PLE (*N* = 768) and individual PLEs with gray and white matter brain structures, controlling for sex, age, intracranial volume, scanning site, and position in the scanner. Individuals that reported having experienced auditory hallucinations (*N* = 272) were found to have smaller volumes of the caudate, putamen, and accumbens (*β* = −0.115–0.134, *p*_corrected_ = 0.048–0.036), and reduced temporal lobe volume (*β* = −0.017, *p*_corrected_ = 0.047) compared to those that did not. People who indicated that they had ever believed in unreal conspiracies (*N* = 111) had a larger volume of the left amygdala (*β* = 0.023, *p*_corrected_ = 0.038). Individuals that reported a history of visual PLEs (*N* = 435) were found to have reduced white matter microstructure of the forceps major (*β* = −0.029, *p*_corrected_ = 0.009), an effect that was more marked in participants who reported PLEs as distressing. These associations were not accounted for by diagnoses of psychotic or depressive illness, nor the known risk factors for psychotic symptoms of childhood adversity or cannabis use. These findings suggest altered regional gray matter volumes and white matter microstructure in association with PLEs in the general population. They further suggest that these alterations may appear more frequently with the presentation of different psychotic symptoms in the absence of clinically diagnosed psychotic disorders.

## Introduction

Psychotic symptoms can manifest in various psychiatric disorders such as schizophrenia, bipolar disorder and depression^1,2^. People can however also experience similar “psychotic-like symptoms” (PLEs), which can occur in the absence of psychiatric disorders^3,4^. The lifetime prevalence of such PLEs in the general population is estimated to range from 7 to 13%^5^. PLEs can mark a prodromal phase of a full first psychotic episode, and the estimated risk of developing a psychotic disorder in those with PLEs is around 30–40%^6^. This risk however varies depending on several other factors, of which childhood adversity and cannabis use further increase risk^5^. The high prevalence of PLEs and their possible contribution to the development of diagnosable psychotic disorders highlights the importance of developing a better understanding of their neurobiological concomitants.

There has been extensive research on gray matter volumes in groups of patients with schizophrenia, compared to healthy controls, with most studies indicating reduced volumes of prefrontal and temporal lobes, and of total gray matter, that continues to decline over time^7–10^. Studies of individuals at high risk of developing a psychotic disorder with sub-threshold psychotic symptoms, also indicate smaller gray matter volumes in these regions. For instance, Jacobson et al.^11^ found gray matter increases within the middle and superior temporal gyri, angular gyrus, orbitofrontal gyrus, as well as gray matter decreases in the inferior temporal gyrus, in children aged 11–12 at symptomatic risk for psychosis. In a more recent study, Calvo et al.^12^ reported reduced bilateral hippocampal volume in adolescents who reported psychotic experiences. White matter decreases within this group included the inferior fronto-occipital fasciculus, cingulum, and inferior longitudinal fasciculus^11^. Further, O’Hanlon et al.^13^ investigated white matter microstructural differences in 28 adolescents aged 13–16 years with self-reported psychotic experiences and 28 adolescents with no self-reported symptoms, and found differences between the two groups in the uncinate fasciculus as well as in striatal regions close to the putamen^13^. Further studies investigating adolescents at high risk of psychosis found similar results^14,15^.

Studies using the Enhancing Neuroimaging Genetics through Meta-Analysis (ENIGMA) consortium have shown smaller subcortical volumes in patients with schizophrenia (*N* = 2028) as compared to healthy controls (*N* = 2540), including in the hippocampus, amygdala, thalamus, and accumbens, as well as smaller intercranial volumes^16^. Cortical differences using the ENIGMA consortium were also identified in patients with schizophrenia, specifically global cortical thinning, with the largest effect sizes within frontal and temporal lobe regions^17,18^. Despite findings from large-scale consortia, studies to date have not had sufficient power to relate particular symptom types to specific regional alterations^19,20^. Consistent relationships have however been found in schizophrenia between the severity of auditory verbal hallucinations (AVHs) and reduced temporal lobe volumes, especially of the superior temporal gyrus^21–24^. There is also evidence that AVHs in schizophrenia and Parkinson’s disease may map on to different parts of the temporal lobes—particularly the hippocampus in the latter case^25^. These results indicate potentially specific associations between volumes of brain structures and particular psychotic symptoms in different neuropsychiatric disorders. As far as we are aware, however, there are no consistent gray matter correlates of visual hallucinations or different types of delusion in schizophrenia, in other psychiatric disorders, or in the general population. Insights into whether these correlates would differ from AVHs could give us valuable information regarding a possible differential neuroanatomy of psychotic symptoms.

Other studies have tested the hypothesis that abnormal white matter connectivity is correlated with schizophrenia^26,27^. Kelly et al.^28^ utilized the ENIGMA consortium and identified widespread reductions in fractional anisotropy (FA) in patients with schizophrenia (*N* = 1963) as compared with healthy controls (*N* = 2359), with the greatest effects shown in the anterior corona radiata, and body and genu of the corpus callosum^28^. Clark et al.^29^ showed that, compared to healthy controls, schizophrenia patients had higher mean diffusivity (MD) in the right anterior thalamic radiations, forceps minor, bilateral inferior fronto-occipital fasciculus, and bilateral uncinated. In addition, FA alterations were shown in the bilateral inferior fronto-occipital fasciculus, as well as the left inferior and superior longitudinal fasciculi^29^.

Associations have also been reported between various psychotic symptoms and altered white matter microstructure, such as between delusions and the cingulum^30,31^, but no consistent pattern has emerged. Many studies have found reduced white matter microstructure in schizophrenia in the uncinate fasciculus^32,33^, arcuate fasciculus^34^, frontal networks^35–37^, and projection fibers^38^. However, several meta-analyses have reported that these disruptions are often associated with increased age and duration of illness, rather than specific symptoms^38–40^. For example, studies that have focussed specifically on AVHs have observed reduced fractional anisotropy (FA) in some tracts^41,42^, while others have found higher FA values in auditory and other pathways^43–46^. The lack of consistent gray and white matter correlates of hallucinations and delusions in psychotic disorders, apart from the association between the superior temporal gyrus and AVHs, could be attributable to the small number of studies and their small sample sizes as well as the clinical heterogeneity of patients with different symptoms and diagnoses. Regarding subclinical psychotic symptoms, research shows that there are subtle overall reductions in efficiency and connectivity of white matter networks related to psychotic experiences (see ref. ^47^, O’Neill et al. 2020). Certainly, very few studies have looked at different types of PLEs and they tend to focus on children and young adolescent populations (see refs. ^48,49^, Kӓrcher et al. 2019). This can in turn be traced to difficulties in the recruitment and scanning of large samples that suffer from psychotic symptoms. Here, we have taken advantage of the very large sample of the UK Biobank (UKB) who have had structural magnetic resonance imaging (MRI) brain scans and PLE assessments. Another advantage of focusing on this general population, is that it reduces the susceptibility to the potentially confounding effects of diagnoses, such as schizophrenia and its associations with a range of factors including childhood adversity, cannabis use, and antipsychotic medication-related changes.

The aim of the current study was therefore to determine possible gray and white matter associations of the four different types of PLEs reported in UKB in a large sample of the general population. More specifically, we chose to investigate global, regional, and individual cortical and subcortical areas, as well as global, tract category, and tract-specific white matter microstructure. Because of the size of the UKB study, we were also able to examine possible contributing effects of childhood adversity, cannabis use, or distress related to the experience of PLEs in the absence of the full disorder.

## Materials and methods

Please see the Supplementary Tables 1–110 for additional analyses.

### Participants

The data were provided by the UK Biobank^50^ and the October 2018 release was used for this study. This contained imaging-derived phenotypes (IDPs) of *N* = 21,390 participants and mental health questionnaire data of 157,366 participants. The participants’ age ranged from 40 to 69 years upon recruitment. Details on image acquisition can be found in the supplementary material and in previous publications^51,52^. Table 1 shows the four specific questions targeting psychotic-like experiences. Individuals were asked if they had ever experienced visions, voices, communications, and conspiracies, representing lifetime reporting of the four PLEs. We determined the number of people that answered positively to at least one of the questions about ever having experienced either a vision, a voice, a conspiracy or a communication (“any PLE”). We then split that group into the four different psychotic symptom types, depending on whether they answered positively to the questions about particular types of PLEs (see Table 2). Participants with incomplete answers were excluded. Subjects were also asked to rate how distressing these experiences were (see Table 1).

**Table 1:** Questions from UK Biobank Mental Health Questionnaire about psychotic experiences.

Question	Type of PLE	Clinical relevance
Did you ever see something that wasn’t really there that other people could not see?	Visions	Visual hallucinations
Did you ever hear things that other people said did not exist, like strange voices coming from inside your head talking to you or about you, or voices coming out of the air when there was no one around?	Voices	Auditory hallucinations
Did you ever believe that a strange force was trying to communicate directly with you by sending special signs or signals that you could understand but that no one else could understand (for example through the radio or television)?	Communications	Delusions of reference
Did you ever believe that that there was an unjust plot going on to harm you or to have people follow you, and which your family and friends did not believe existed?	Conspiracies	Paranoid delusions
If any of the questions above was answered with “Yes”: How distressing did you find having any of these experiences (seeing a vision, hearing a voice, or believing that something strange was trying to communicate with you, or there was a plot against you)?^a^		

For all four questions, it was specified in the subtext that the subject should not include any times where the subject could have been half-asleep or under the influence of drugs or alcohol. The possible answers participants could choose from were: Yes, No, Prefer not to answer, Do not know. Column 3 represents the clinical term to which PLEs in column 2 correspond; it is important to note that the PLEs in column 2 do not completely encompass the symptoms in column 3 (e.g., auditory hallucinations may also represent noises, and not just voices).

^a^Options subject could choose from: Not distressing, a neutral experience/A bit distressing/Quite distressing/Very distressing/Do not know/Prefer not to answer.

**Table 2:** Prevalence of the different types of PLE in those that completed the UKB mental health questionnaire (*N* = 157,366 with 89,101 women) (see Supplementary Tables 1 and 2 for the numbers on different imaging data types).

PLE type	N. of participants with PLE (%)	N. of females with PLE (%)	N. (%) of those with PLEs with any form of psychosis	N. (%) of those with PLEs with self-reported depression	N (%) of participants that found PLE distressing	N. of participants with PLE and (sub)cortical imaging data	N. of participants with PLE and FA and MD imaging data
Vision	5031 (3.20%)	3183 (63.3%)	232 (4.6%)	1975 (39.3%)	1647 (32.7%)	444	417
Voices	2777 (1.76%)	1658 (59.7%)	280 (10.1%)	1316 (47.4%)	1146 (41.3%)	272	254
Communication	1138 (0.72%)	629 (55.3%)	245 (21.5%)	533 (46.8%)	472 (41.5%)	100	94
Conspiracy	1262 (0.80%)	599 (47.5%)	283 (22.4%)	771 (61.1%)	1046 (82.9%)	111	106
Any PLE	7803 (4.96%)	4718 (60.5%)	458 (5.9%)	3239 (41.5%)	3012 (38.6%)	712	671

Note that diagnoses are based on self-report.

*PLE* psychotic like experience, *SCZ* schizophrenia.

UK Biobank data acquisition was approved by Research Ethics Committee (reference 14/NW/0382). The analysis and data acquisition for the present study were conducted under application 16,124, linked to #4844. Written consent was obtained for all participants.

### Image acquisition

The imaging data that were used for this study consisted of quality-controlled IDPs that were generated and provided by UK Biobank, with the exact procedure of acquisition, quality checking (e.g., exclusion due to segmentation and cortical parcellation errors) described elsewhere (for details, see supplementary materials)^53^. The gray matter segments that were obtained were subsequently segmented into 34 cortical and 14 subcortical regions per hemisphere, following the Desikan-Killany atlas^54^. The DTI data from UK Biobank provided tract-averaged fractional anisotropy and mean diffusivity of 12 bilateral and 3 unilateral major tracts.

### Statistical analysis

Statistical analysis was performed in R version 3.2.3 (https://cran.r-project.org) within a Linux environment. For a more detailed description of methods, see supplementary materials. Before analysis was performed, outliers were removed^55^. For the gray matter data, this was done by looking at the intracranial volume (ICV). The ICV was calculated by adding the total volume of gray matter, white matter and ventricular cerebrospinal fluid, all normalized for head size, when looking at the volume of the subcortical and cortical regions. Subjects with ICVs that deviated three standard deviations above or below the mean were excluded. This resulted in a maximum of *N* = 14,375 participants remaining with cortical and subcortical imaging data and PLE data. For DTI data this was achieved by performing separate principal component analysis for each DTI measure on the overall sample of N = 19,345, those whose score for the first principal component were outside of +/− 3 standard deviations from mean were removed^55^. This resulted in a maximum of *N* = 13,877 participants remaining with DTI data and PLE information.

Our overall approach was to examine associations between the imaging variables of all four modalities (cortical, subcortical, FA, and MD) and “any-PE”, and then associations with each of the four PLE types separately. We used the “lme” function in R to test for associations between the structures and PLEs. Sex, age, age², scanner site, ICV, and the scanner positions *x*, *y*, and *z* were added to the model as covariates (Neilson et al. 2019). For bilateral structures, we used a repeated-effect linear model where hemisphere was added as a covariate. Additionally, we inspected the interaction effect between PLEs and hemisphere. If these interactions were significant, separate analyses on hemispheres were performed^55^.

Effect sizes were standardized throughout. *p*-values were corrected by applying the false discovery rate (FDR) at a rate of *p* < 0.05, using the p.adjust function, as per supplementary materials. These corrections were applied over the individual brain regions for each imaging modality (34 cortical regions, 7 subcortical regions, 15 white matter tracts (split into 12 bilateral tracts and 3 unilateral tracts)). Additionally, we applied the FDR correction over the global and regional measures for each imaging modality (4 lobes for cortical measures and 1 global and 3 tract categories for white matter measures).

### Gray matter

We first of all looked at associations between PLEs and imaging measures at an increasing level of regional detail. This involved first testing associations between PLEs and lobar brain regions, obtained by adding up the volume of cortical segments (see Supplementary material Table 3), then individual cortical segments and then individual subcortical gray matter volumes. We used the R package “lme” for the analysis of bilateral regions (where hemisphere was controlled for, after testing and ensuring there were no any significant hemisphere interaction effects) and each bilateral region modeled as a repeated measure^56^.

### White matter

As per the gray matter analysis, associations were tested at increasing levels of regional detail The first linear models that we applied examined whole brain general FA and MD measures, followed by the general FA and MD of the three subsets of white matter tracts categorized as association fibers, thalamic radiations, and projection fibers^55^. We also applied a repeated-effect linear model to inspect the bilateral structures individually, as with the cortical structures. Again, interaction effects with hemisphere and sex were also examined, and FDR correction was applied to correct for possible false positives.

### Additional analyses

We inspected interaction effects between sex and PLE type to examine whether differences between sexes might influence our findings. We ran separate models with the interaction effect as a predictor for the brain region, using the same covariates as the models used in the main analysis described above. Additionally, given the evidence that distress caused by a PLE may mark the clinical significance of a psychotic symptom, we examined the effects of distress in association with PLEs. We performed a separate analysis, where we included only those who reported PLEs as distressing (as opposed to neutral or positive) versus those who reported no PLE to determine whether experiencing stress would impact on the size of the standardized coefficients for the different structures and regions.

We additionally examined whether our main findings could be attributable to the possibly confounding effects of childhood adversity, cannabis use, antipsychotic medication, and self-declared diagnosis of any psychotic disorder or depression. We added these factors as covariates to our model to determine their impact/contribution on our main findings and results. A mediation analysis was performed if these exploratory analyses revealed possible influences of any of these factors. We controlled for depression by both looking at a possible interaction effect with PLEs, as well as including it as a covariate to the model, due to recent studies working with the UK Biobank sample that have shown altered white matter microstructure associated with MDD^55^. These analyses are described in more detail in the supplementary material.

## Results

### PLE frequencies in the cohort

Table 2 outlines the main results on the mental health questionnaire. In total, *N* = 7803 (4.96%) of the participants reported ever having had any PLE. The proportion of people that indicated they had ever seen an unreal vision (*N* = 5031, 3.20%) was the single largest group. The mean age and standard deviation were similar across all four PLE types (63.12 ± 7.4 years). With regards to distress, 38.6% (*N* = 3012 subjects) found any PLE distressing, whereas the “conspiracy” group had the largest proportion of subjects who found the experience distressing (*N* = 1046, 82.9%).

When combining the subjects that had completed the mental health questionnaire with the available imaging data, the ratio of PLEs remained roughly the same, see Table 2. For gray matter imaging data, there were 712 (5.0%) who reported any PLE, of which 444 (3.2%) reported a visual PLE. With regards to white matter imaging data, 671 (4.8%) reported a history of any PLE, with 417 (3.0%) reporting a visual PLE (see supplementary material Tables 1 and 2 for sample sizes for all PLE types).

### Gray matter—cortical volumes

#### Any PLE

We did not find any statistically significant associations between any PLE and cortical brain volumes (*β* = −0.088–0.042, *p*_corrected_ = 0.148–0.959).

#### Individual PLE types

**Fig. 1:**
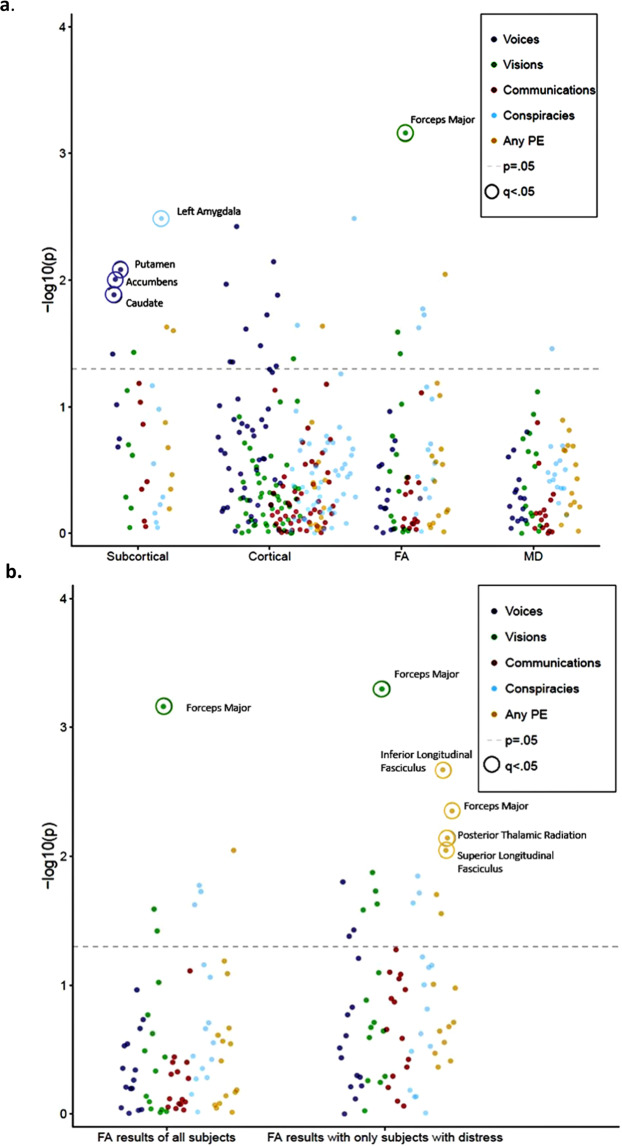
Summary of results. **a** Summary of results of significant differences between groups with and without PLE of different types for each imaging type. Each colored dot represents the adjusted *p*-value of different types of PLEs. Encircled dots (q) illustrate significant findings (with *p*_corrected_ < 0.05). **b** Significant associations of FA differences in all subjects and those reporting distress during PLEs. Each colored dot represents the adjusted *p*-value of different types of PLEs. Encircled dots (q) illustrate significant findings (with *p*_corrected_ < 0.05).

For the individual PLE types, there was a statistically significant reduced overall volume of the GM in the temporal lobe in people who had experienced hearing unreal voices (*β* = −0.017, *p*_corrected_ = 0.038) compared to those who had not (Table 3). Among the other PLE types, none of the lobes showed any significant differences in volume (see Fig. 1a).

**Table 3:** The associations between “voices” (*N* = 254) and lobar volumes.

Lobes	*p*-value	FDR *p*-value	Β-coefficient
Temporal lobe*	0.009	0.038	−0.017
Occipital lobe	0.043	0.108	−0.013
Parietal lobe	0.245	0.245	−0.008
Frontal lobe	0.076	0.114	−0.012

*FDR* false discovery rate.

*p_corrected_ < 0.05.

### Gray matter—subcortical volumes

#### Any PLE

None of the subcortical volumes were significantly different in those that reported any PLE versus those that did not (*β* = −0.075–0.040, *p*_corrected_ = 0.072–0.685).

#### Individual PLE types

In the separate PLE type analyses, those who indicated ever having heard an unreal voice (*N* = 272) had significantly smaller volumes of the caudate, putamen, and accumbens than those who did not (*β* = −0.128, *β* = −0.134, *β* = −0.115 respectively, *p*_corrected_ = 0.048 for the caudate and *p*_corrected_ = 0.036 for putamen and accumbens—see Table 4).

**Table 4:** The association between “visions” (*N* = 417) and (1) subcortical volumes and (2) fractional anisotropy values of white matter tracts.

Brain Structure	*P*-Value	FDR *P*-value	Β-coefficient [95% CI]
Subcortical volumes			
Thalamus	0.051	0.090	−0.097 [−0.195;0.001]
Caudate*	**0.020**	**0.048**	**−0.128** [**−0.236;−0.020]**
Putamen*	**0.007**	**0.036**	**−0.134** [**−0.232;−0.036]**
Pallidum	0.091	0.128	−0.091 [−0.196;0.015]
Hippocampus	0.264	0.264	−0.058 [−0.159;0.044]
Amygdala	0.243	0.264	−0.057 [−0.153;0.039]
Accumbens*	**0.010**	**0.036**	**−0.115** [**−0.203;−0.027]**
White matter microstructure			
Acoustic Radiation	0.412	0.772	−0.035 [−0.118;0.048]
Anterior Thalamic Radiation	0.788	0.977	−0.012 [−0.101;0.077]
Cingulate gyrus – part of cingulum	0.197	0.592	−0.049 [−0.124;0.026]
Parahippocampal part of cingulum	0.844	0.977	−0.008 [−0.091;0.075]
Corticospinal Tract	0.970	0.977	0.002 [−0.088;0.091]
Inferior fronto-occipital fasciculus	0.315	0.772	−0.045 [−0.133;0.043]
Inferior longitudinal fasciculus	0.035	0.262	−0.094 [−0.181;−0.007]
Medial Lemniscus	0.542	0.904	−0.026 [−0.108;0.057]
Posterior Thalamic Radiation	0.061	0.304	−0.084 [−0.172;0.004]
Superior Longitudinal fasciculus	0.153	0.573	−0.061 [−0.144;0.023]
Superior Thalamic Radiation	0.729	0.977	0.016 [−0.076;0.109]
Uncinate Fasciculus	0.977	0.977	−0.001 [−0.086;0.083]
Forceps Major*	**0.001**	**0.009**	**−0.029** [**−0.008;−0.002]**
Forceps Minor	0.403	0.772	−0.007 [−0.003;0.001]
Middle Cerebellar Peduncle	0.884	0.977	−0.001 [−0.003;0.003]

*CI* Confidence Interval, *FDR* false discovery rate, *BOLD p*_corrected _ < 0.05.

*p_corrected_ < 0.05.

Individuals that reported ever having believed in an unreal conspiracy (*N* = 111) showed significant hemisphere-group interaction effects for the volume of the amygdala (*β* = −0.219, *p* = 0.041), so we therefore conducted our analyses on the lateralised structures separately. This model showed that the left amygdala was significantly bigger (*β* = 0.023, *p*_corrected_ = 0.049) in those that believed in an unreal conspiracy compared to those that did not, with no significant difference found in the right amygdala (*β* = 0.005, *p*_corrected _= 0.702).

Both the unreal communications and visions group did not show any significant differences between groups in association with subcortical volumes (see Fig. 1a).

#### Additional analyses

There were no significant sex-PLE interaction effects after correction. There were also no significant changes to any of these effects when only including those who reported the PLE as distressing. Since both childhood adversity and cannabis use were higher among the groups with PLEs, these factors were added as covariates to the model to see whether this would have an effect on our results. Adding the covariates only slightly increased the adjusted *p*-values and effect sizes. A mediation analysis for either cannabis use or childhood adversity on the association between putamen volume and “voices” found only direct significant effects (see supplementary materials).

### White matter—fractional anisotropy

#### Any PLE

None of the effects of PLEs on FA values of the subsets of white matter tracts were statistically significant. There was a general trend towards lower FA values for those with any PLE, but none of the differences were statistically different.

#### Individual PLE types

Within separate PLE modalities, again the subsets of white matter tracts did not render any significant results. However, when examining the individual tracts, the FA of the forceps major was significantly lower in the group who reported ever having an unreal vision (*β* = −0.028, *p*_corrected_ = 0.010) compared to subjects who do not report having had a “vision” (see Table 4).

### White matter—mean diffusivity

We generally observed higher values of mean diffusivity in both subsets and individual tracts of white matter among those that had experienced psychotic like symptoms, but none of the white matter tract MD values were significantly associated with any PLE. We also did not find any significant results for the individual tracts (all *p*_corrected_ > 0.05). The reporting of distressing PLEs did not significantly alter these results.

### Additional analyses

We did not find any significant interaction effects between a self-declared diagnosis or treatment of psychosis or depression and PLEs for either (sub)cortical and white matter imaging data. Adding either childhood adversity or cannabis use as covariates to the model only resulted in slight increases of the adjusted *p*-values.

When only taking the cases that reported the PLE as “distressing”, there were stronger associations between lower FA values and the group with any PLE for the inferior longitudinal fasciculus, posterior thalamic radiation, superior longitudinal fasciculus, and the forceps major (*β* = −0.17, *p*_corrected_ = 0.03, *β* = −0.14, *p*_corrected_ = 0.03, *β* = −0.14, *p*_corrected_ = 0.03, *β* = −0.023, *p*_corrected_ = 0.03, respectively (see Fig. 1b and supplementary materials).

## Discussion

We investigated whether there are structural differences in subcortical and cortical brain structures and white matter tracts between people with and without psychotic-like experiences of four different types. In what is we believe the largest study to date of the neuroimaging associations of PLEs, we found associations between reduced (sub)cortical volumes and “voices”, an enlarged amygdala and “conspiracies”, and reduced white matter microstructure with “visions” and “any PLE” in subjects who had experienced distress (see Fig. 1b). No associations were found with “communications”. These associations are unlikely be accounted for by diagnoses of psychotic or depressive illnesses or other possible confounders such as known risk factors for psychotic symptoms like childhood adversity or cannabis use.

### Gray matter

The temporal cortical structures together showed a statistically significant decrease in volume in those who reported auditory hallucinations, whereas individual temporal lobe structures did not show significant differences. This is consistent with knowledge that the structures and networks that process auditory stimuli are located in the temporal lobe^17,18,57^. Decreases in temporal lobe volume have previously been found in several psychotic disorders^25^, in subjects at high risk^19^ and in first episode psychosis patients^6^ and have been specifically linked to AVHs in schizophrenia^21^. In a previous study focussing on PLEs in young adults, without clinically diagnosed psychotic disorders, the largest reductions were found in the temporal lobe, and especially among subjects that had persistent PLEs^49^—findings that are in line with the results of the present study. Taking these results together with those reporting AVH associations with the hippocampus in Parkinson’s disease^25^ suggests that reductions in different parts of the temporal lobe may be differentially linked with auditory hallucinations in different conditions, rather than with schizophrenia or psychotic disorders per se.

The finding of a significantly enlarged left amygdala among people that had ever believed in unreal conspiracies is, as far as we are aware, a new finding. There are indications that the volume of the amygdala is altered in a range of psychiatric disorders, with an enlarged amygdala in some autism studies and a smaller amygdala in patients with schizophrenia^58^. Some schizophrenia studies have related reduced volume to social anxiety^59^ or to specific positive symptoms such as persecutory delusions^60^. In healthy populations, the size of the amygdala has been positively related to the size and complexity of people’s social network^61^. Believing in conspiracies could conceivably be associated with greater interest in complex social networks, and thus linked to the observed increase of amygdala volume in participants reporting a “conspiracy PLE”^61^. It could also be related to findings of functional MRI studies that patients with schizophrenia^62^, and perhaps those with persecutory delusions in particular, show an increased response to neutral stimuli in the amygdala^63^. Further studies are required to replicate these results and to test these potential ties.

We also found volume reductions in the putamen, the caudate and the nucleus accumbens in the group of subjects with a history of “voices”. Smaller caudate volume has previously been reported in first episode psychosis^64^, with further decreases as psychotic symptoms worsen^65^. There are also studies reporting larger volumes of striatal structures in schizophrenia^66^ but these tend to be linked to antipsychotic drug exposure. A larger accumbens has been linked resilience to subclinical symptoms^67^, while decreased volume of the putamen has been associated with AVHs in the context of schizophrenia^68^.

We ran several analyses to check that known risk factors for psychotic symptoms did not account for the neuroimaging associations we found. Distress affected less than half of the people who reported having experienced a PLE, with the exception of the “conspiracies” PLE, and did not alter gray matter associations. Childhood adversity and cannabis use were higher among the groups with PLEs but adding them as covariates only slightly increased the adjusted *p*-values. A mediation analysis for either cannabis use or PLEs on the association between putamen volume and “voices” found only direct significant effects. This indicates that both lifetime cannabis use and PLEs are independently related to the volume of brain structures such as the putamen, and the association we found was not simply attributable to cannabis use. This is in line with the many studies that have found that cannabis use impacts upon brain function in people without psychiatric disorder^69^, those at high familial risk^70^ and in people with schizophrenia^71^. The effects of familial risk, cannabis exposure, PLEs, and schizophrenia may therefore be dissociable.

### White matter

We did not find any significant associations of psychotic symptoms with MD values after applying FDR correction. We did however find a significantly lower FA of the bilateral forceps major in the group that had experienced unreal visions. There are several reports of cerebrovascular disease of the forceps major as a likely cause of visual hallucinations^72^, in keeping with our findings. Decreased FA values in the forceps major have previously been found in schizophrenia and other psychotic disorders^73^, and in early psychosis^74^, but no previous study has, as far as we are aware, linked that to visual or other hallucinations in those populations.

Interestingly, there were more significant white matter associations for any PLE when only including those that reported distress when experiencing a psychotic symptom. As shown in Table 1, the “conspiracy” PLE was reported most commonly as distressing by UKB subjects, even though hallucinations are usually reported as most distressing by patients with schizophrenia^75^. Distress can play an important role in the outcome of experiencing subclinical psychotic symptoms^76^, but our results are not generally driven by distress. Given that previous studies have linked reduced FA values in the forceps, superior, and inferior longitudinal fasciculi with psychotic symptom severity in early psychosis^74^ and in those at high clinical risk^77^, it is possible that such findings could be utilized to observe the development of psychotic disorders.

### Limitations

Some limitations of the present study should be considered. Above all, the sample consists of people over 40 years old and with a mean age of 63 years. Therefore our results are indicative of lifetime brain pattern changes, rather than identifying biological markers for people at risk or direct consequences of drug use and distress. In addition, the mental health questionnaire reports lifetime occurrence of PLEs, and not a specific timepoint. Thus, our results may be due to developmental changes across the lifespan, and may not apply to younger populations. Secondly, as the current paper investigated individuals with PLEs compared to those without PLEs, we did not include matched healthy controls in our analyses. Another potential issue is the fact that the measures of psychotic-like experiences and any associated distress or diagnosis are based on historical self-report. The relatively simple questions about possible PLEs might have resulted in lower reliability and validity compared to clinical or self-rating instruments. This would however have likely added noise to our analyses, in that some people may have had symptoms or diagnoses but did not describe them, and may thereby have obscured some associations. Further, due to the format of the questionnaire, it is not clear whether participants’ PLEs diverge from normal experiences or represent a symptom of psychiatric disorders. Similarly, the relatively small sample sizes of some of the groups with particular psychotic symptoms and distress or certain risk factors may also have reduced power to find some associations. Lastly, we did not report sensitivity analyses using a subset of individuals with no reported distress, although sensitivity analyses carried out in the subset who reported distress showed there was no association between the behavior and PLEs.

## Conclusions

This is one of the first studies to look at the associations of individual psychotic symptoms with neuroimaging indices in the general population—and the largest to date. We have found associations of “voices” with subcortical and temporal lobe structures in keeping with the schizophrenia literature. We also report two novel findings: an association between delusion like ideas about conspiracies and a large amygdala, and reduced white matter microstructure in the forceps major in people suffering from visual PLEs. Replication is needed for these novel findings, but they could represent specific alterations in the presence of such symptoms. Greater differentiation of the anatomical and functional correlates of different psychotic symptoms in different disorders could lead to a better understanding of the underlying mechanisms of these psychotic symptoms, and their various forms in subclinical and diagnosable psychotic and neurological disorders.

## Supplementary information

Supplementary materials
