# Supplementary material for: Grey and white matter associations of psychotic-like experiences in a general population sample (UK Biobank)
Source: Transl Psychiatry. 2021 Jan 7;11:21. doi: 10.1038/s41398-020-01131-7 (PMC7791107; doi:10.1038/s41398-020-01131-7)
Supplement: Supplementary file 1 — Supplementary materials [file 41398_2020_1131_MOESM1_ESM.docx]

# Supplementary material to ‘Grey and white matter associations of psychotic experiences in a general population sample’

This supplementary material provides all the results of the analyses performed for this research. Firstly, the general frequencies on different types of PE and several lifestyle factors are displayed. Subsequently we provided additional information on the methods used for image acquisition statistical measures. The results are presented per image modality (subcortical volumes, cortical volumes, FA and MD), starting with the results on ‘Any PE’ before showing the results per PE type (Visions, Voices, Communications and Conspiracies). The main analyses on volume per PE type also report the 95% confidence intervals for the standardized effect sizes. For all these types, results on additional interaction effects are also shown. The significant results are shown in bold. For some of the analyses, additional analyses are added at the end of the section to control for associations and mediating effects.

Content

[Supplementary material to ‘Grey and white matter associations of psychotic experiences in a general population sample’ 1](#_Toc39399761)

[Frequency of PE types per imaging type 4](#_Toc39399762)

[Supplementary Methods 5](#_Toc39399763)

[Supplementary Results 11](#_Toc39399764)

[Grey matter cortical volumes 11](#_Toc39399765)

[Any PE 11](#_Toc39399766)

[Visions 19](#_Toc39399767)

[Voices 26](#_Toc39399768)

[Communications 33](#_Toc39399769)

[Conspiracies 41](#_Toc39399770)

[Grey matter subcortical volumes 48](#_Toc39399771)

[Any PE 48](#_Toc39399772)

[Visions 49](#_Toc39399773)

[Voices 51](#_Toc39399774)

[Communications 53](#_Toc39399775)

[Conspiracies 54](#_Toc39399776)

[Cannabis as mediator 59](#_Toc39399777)

[Fractional anisotropy 61](#_Toc39399778)

[Any PE 61](#_Toc39399779)

[Visions 64](#_Toc39399780)

[Voices 69](#_Toc39399781)

[Communications 71](#_Toc39399782)

[Conspiracies 73](#_Toc39399783)

[Mean Diffusivity 76](#_Toc39399784)

[Any PE 76](#_Toc39399785)

[Visions 78](#_Toc39399786)

[Voices 80](#_Toc39399787)

[Communications 82](#_Toc39399788)

[Conspiracies 84](#_Toc39399789)

[References 87](#_Toc39399790)

## Frequency of PE types per imaging type

| **PE modality group** | **Total N. of subjects per PE with imaging data** | **Sex (F,%)** | **(%) of participants with PE** | **N. of females with PE (%)** |
| --- | --- | --- | --- | --- |
| **Vision** | 13777 | 7902 (57.4%) | 444 (3.2%) | 296 (66.7%) |
| **Voices** | 14037 | 7760 (55.3%) | 272 (1.9%) | 160 (58.9%) |
| **Communication** | 14232 | 7769 (54.2%) | 100 (0.7%) | 53 (53.0%) |
| **Conspiracy** | 14228 | 7766 (54.6%) | 111 (0.8%) | 50 (45.0%) |
| **Any PE** | 14375 | 7785 (54.2%) | 712 (5.0%) | 430 (60.4%) |

Table 1: Basic demographics of groups with (sub)cortical structures data subdivided per PE type, mean age and standard deviation 63.1 ± 7.4 years

| **PE modality group** | **Total N. of subjects per PE with imaging data** | **Sex (F,%)** | **(%) of participants with PE** | **N. of females with PE (%)** |
| --- | --- | --- | --- | --- |
| **Vision** | 13733 | 7410 (54.0%) | 417 (3.0%) | 274 (65.7%) |
| **Voices** | 13820 | 7667 (55.5%) | 254 (1.8%) | 143 (56.3%) |
| **Communication** | 13843 | 7476 (54.0%) | 94 (0.7%) | 48 (51%) |
| **Conspiracy** | 13846 | 7471 (54.0) | 106 (0.8%) | 47 (44.3%) |
| **Any PE** | 13877 | 7486 (54.0%) | 671 (4.8%) | 398 (59.3%) |

Table 2: Basic demographics with FA and MD data subdivided per PE type

# Supplementary Methods

*Imaging data acquisition and processing*

The imaging data that was used for this study consisted of the imaging derived phenotypes (IDPs) that were generated and provided by the UK Biobank, with the exact procedure of acquisition described by Alfaro-Almagro and colleagues. ^1,2^ In summary, the imaging data were obtained with a Siemens Skyra 3T scanner (<https://www.healthcare.siemens.com/magnetic-resonance-imaging>) after which the raw images were pre-processed through usage of a FSL package. The T1-weighted data that was utilized for the analysis in this study were obtained using 3D MPRAGE sequence, with a resolution of 1mm3 of isotropic voxels. For the grey matter volumes, segmentation of the T1-weighted data was performed in two steps for segmentation: cerebrospinal fluid and grey and white matter were first extracted by using FAST (FMRIB’s Automated Segmentation Tool).^3^ Subcortical structures were extracted with the usage of FIRST (FMRIB’s Integrated Registration and Segmentation Tool.^4^ For a more detailed description of our local processing of cortical metrics using Freesurfer, see Neilson and colleagues.^5^

With regards to white matter we looked at the general measures of global FA and MD, three different tract categories and the individual tracts, similar to our previous white matter integrity analysis in UKB.^6–8^ The three tract categories examined were: association and commissural fibres (forceps major and forceps minor, inferior fronto-occipital fasciculus, uncinate fasciculus, cingulum bundle and the superior longitudinal fasciculus), thalamic radiations (superior, posterior and anterior thalamic radiations) and the projection fibres (corticospinal tract, acoustic radiation, medial lemniscus and middle cerebellar peduncle). There was data available for 27 different white matter tracts in total, of which 12 were bilateral and 3 that spanned across the two hemispheres.

*Statistical analysis*

Statistical analysis was performed in R version 3.2.3 (<https://cran.r-project.org>) within a Linux environment. For all four PE symptom types and the group of ‘any PE’, the proportion of people who experienced each symptom, along with the respective male-female ratios, mean ages and standard deviations were determined.

*Grey matter.* For a more thorough understanding of possible differences in cortical volumes we firstly looked at lobar volumes per PE type. To obtain the total volumes of the different lobes, we added the various brain region volumes from UK Biobank, by following the structural mapping and classification as defined by Desikan *et al.* (2006) (see supplementary material ST4). As such, we did a separate statistical analysis on the volumes of the different lobes to examine whether there are different grey matter lobar volumes in people with specific PEs.

For analysis of the individual cortical and subcortical grey matter volumes related to PEs, we applied a linear model to detect possible significant links with having experienced any PE and then each PE of a particular type by utilizing the function ‘lme’ from the package ‘nlme’ (<https://cran.r-project.org/web/packages/nlme/index.html>). The ICV was calculated by adding the total volume of grey matter, white matter and ventricular cerebrospinal fluid, all normalized for head size, when looking at the volume of the subcortical and cortical regions.^7^ Subjects with ICVs that deviated three standard deviations above or below the mean were also excluded, so that outliers that could conceivably influence the data were excluded from the current analysis. We first inspected the bilateral subcortical and cortical volumes by applying a repeated-effects linear model with sex, age, age², hemisphere, intracranial volume (ICV) and the scanner positions x, y and z as covariates. We also examined the hemisphere-group interactions; if there was a significant interaction effect, we analysed the analysis on the bilateral structure separately. Effect sizes were standardized throughout. P-values were corrected by applying the false discovery rate (FDR).^9^

Group-sex interaction effects were also explored per structure or brain region. If the interaction proved to be significant for a certain brain region, we conducted a separate analysis (looking at the association between PEs in males and females separately).

*White matter.* The association between white matter integrity and PEs was tested by means of the same linear model described above with sex, age, age^2^ the positions in the scanner and the MRI site as covariates. The first linear models that we applied examined whole brain general fractional anisotropy and mean diffusivity, followed by the three subsets of white matter tracts (association fibres, thalamic radiations and projection fibres).^6,7^ We obtained the inputs of these four different categories by performing principal component analysis (PCA) on all 27 tracts to get a measure for global white matter integrity and subsequently performing PCA on the three subsets of tracts to get the value of the first un-rotated principal component. These scores were then set as the dependent variable of the general linear model to test the effect of PEs. The variance of the first principal component of each of these PCAs is described in detail in the supplementary material. We also applied a repeated-effect linear model to inspect the bilateral structures individually, as with the cortical structures. We also tested for sex-group interactions; if any of these proved to be significant, we tested for those tracts separately. Again, FDR correction was applied to correct for possible false positives.

*Additional analyses.*

Since the present study focusses primarily on associations with PEs as a subclinical symptom, we additionally performed a separate analysis where we excluded participants that had self-reported diagnoses of any form of psychosis, following a question in the Mental Health Questionnaire that asked whether the subject has ever been diagnosed with one or more of the following mental health problems by a professional. This question gave them to option to select either ‘Schizophrenia’ or ‘Any other type of psychosis or psychotic illness’. Additionally, we examined possible interaction effects with depression to see whether that could have an effect on our results.

As the literature also suggests that there are several possible environmental factors that could potentially confound our results,^10^ we examined the effects of several demographic and lifestyle factors (see tables 4-7), obtained through additional questions in UK Biobank’s mental health questionnaire. In particular, histories of cannabis use and adverse life events were substantially higher in the group with PEs, these were therefore added as covariates to our models to determine their influence on current findings. We also explored whether cannabis mediated the relationship between a PE and imaging features. The significantly associated (subcortical) brain structures were tested for significant effect of cannabis use in a separate lme model. If that result was significant and there was a significant result for χ² tests between cannabis use and the presence of PEs, we applied a mediation model to determine possible mediating effects of either cannabis or PEs using the package ‘lavaan’ (<https://cran.r-project.org/web/packages/lavaan/lavaan.pdf>).

| **Temporal** | **OCCIPITAL** | **PARIETAL** | **FRONTAL** | **OTHER** |
| --- | --- | --- | --- | --- |
| Temporal Pole | Lateral Occipital Cortex | Postcentral Gyrus | Frontal Pole | Insular Cortex |
| Superior Temporal Gyrus | Intracalcarine Cortex | Superior Parietal Lobule | Superior Frontal Gyrus | Angular Gyrus |
| Middle Temporal Gyrus | Cuneal Cortex | Supramarginal Gyrus | Middle Frontal Gyrus | Subcallosal Cortex |
| Inferior Temporal Gyrus | Lingual Gyrus | Angular Gyrus | Inferior Frontal Gyrus | Paracingulate Gyrus |
| Parahippocampal Gyrus | Occipital Fusiform Gyrus | Precuneous Cortex | Precentral Gyrus | Cingulate Gyrus |
| Temporal Fusiform Cortex | Occipital Pole | Parietal Operculum Cortex | Frontal Medial Cortex | Ventral Striatum |
| Temporal Occipital Fusiform Cortex |  | Supracalcerine Cortex | Juxtapositional Lobule Cortex | Brain Stem |
| Planum Polare |  |  | Frontal Orbital Cortex | Cerebellum |
| Heschls Gyrus |  |  | Frontal Operculum Cortex |  |
| Planum Temporale |  |  | Central Opercular Cortex |  |

Table 3 Cortical structures per lobe, following the structural mapping and classification as defined by Desikan and colleagues ^11^

| **Ever taken cannabis** | **No PE**  **(n= 148,349)** | **Any PE**  **(n=7803)** | **Visions**  **(n= 5031)** | **Voices**  **(n=2777)** | **Communications**  **(n=1138)** | **Conspiracies**  **(n=1262)** |
| --- | --- | --- | --- | --- | --- | --- |
| **More than 100 times** | 3804 (2.5%) | 404 (5.2%) | 238 (4.7%) | 178 (6.4%) | 81 (7.1%) | 113 (9.0%) |
| **11 – 100 times** | 6446 (4.3%) | 551 (7.1%) | 324 (6.4%) | 223 (8.0%) | 82 (7.2%) | 118 (9.4%) |
| **3-10 times** | 7988 (5.3%) | 616 (7.9%) | 423 (8.4%) | 229 (8.2%) | 84 (7.4%) | 89 (7.1%) |
| **1-2 times** | 13,863 (9.3%) | 986 (12.6%) | 634 (12.6%) | 369 (13.3%) | 131 (11.5%) | 177 (14.0%) |
| **Never** | 117,246 (78.4%) | 5233 (67.1%) | 3402 (67.6%) | 1774 (63.9%) | 758 (66.6%) | 762 (60.4%) |
| **Prefer not to answer** | 216 (0.1%) | 13 (0.2%) | 10 (0.2%) | 4 (0.1%) | 2 (0.2%) | 3 (0.2%) |

Table 4: Numbers on reports of cannabis use per PE type

| **Trauma** | **No PE**  **(N= 148,349)** | **Any PE**  **(N=7803)** | **Visions**  **(N= 5031)** | **Voices**  **(N=2777)** | **Communications**  **(N=1138)** | **Conspiracies**  **(N=1262)** |
| --- | --- | --- | --- | --- | --- | --- |
| **Childhood** | 66,461 (43.3%) | 4783 (63.9%) | 3039 (62.9%) | 1814 (68.4%) | 711 (65.1%) | 871 (72.2%) |
| **Adult** | 75,675 (52.7%) | 5284 (71.2%) | 3372 (70.5%) | 1968 (74.6%) | 809 (74.6%) | 960 (79.5%) |
| **Catastrophic** | 74,355 (49.7%) | 5439 (69.7%) | 3502 (69.6%) | 2013 (72.5%) | 802 (70.5%) | 938 (74.3%) |
| **Total** | 120,213 (81.8%) | 7216 (93.7%) | 4643 (93.5%) | 2609 (94.9%) | 1048 (93.5%) | 1196 (95.8%) |
| **None** | 26,731 (18.2%) | 488 (6.3%) | 321 (6.5%) | 140 (5.1%) | 73 (6.5%) | 53 (4.2%) |
| **Prefer not to answer** | 2619 (1.7%) | 99 (0.06%) | 67 (0.04%) | 28 (0.02%) | 17 (0.01%) | 13 (0.01%) |

Table 5: Number on reports of traumatic events per PE type

| **Alcohol consumption behaviour** | **No PE**  **(n= 149,563)** | **Any PE**  **(n=7803)** | **Visions**  **(n= 5031)** | **Voices**  **(n=2777)** | **Communications**  **(n=1138)** | **Conspiracies**  **(n=1262)** |
| --- | --- | --- | --- | --- | --- | --- |
| **Daily or almost daily** | (23.4%) | (20.1%) | 992 (19.7%) | 533 (19.2%) | 213 (18.7%) | 257 (20.4%) |
| **Three or four times a week** | (26.2%) | (22.2%) | 1108 (22.0%) | 566 (20.4%) | 251 (22.1%) | 253 (20.1%) |
| **Once or twice a week** | (24.9%) | (24.2%) | 1247 (24.8%) | 672 (24.2%) | 266 (23.4%) | 271 (21.5%) |
| **One to three times a month** | (10.9%) | (12.4%) | 620 (12.3%) | 349 (12.6%) | 156 (13.7%) | 148 (11.7%) |
| **Special occasions only** | (9.0%) | (12.7%) | 651 (12.9%) | 388 (14.0%) | 142 (12.5%) | 177 (14.0%) |
| **Never** | (0.05%) | (8.2%) | 407 (8.1%) | 265 (9.5%) | 108 (9.5%) | 154 (12.2%) |
| **Prefer not to answer** | 43 (0.03%) | 8 (0.1%) | 6 (0.2%) | 4 (0.11%) | 2 (0.009%) | 2 (0.08%) |

Table 6: Number on reports of alcohol use per PE type

| **Medication** | **No PE**  **(N= 148,349)** | **Any PE**  **(N=7803)** | **Visions**  **(N= 5031)** | **Voices**  **(N=2777)** | **Communications**  **(N=1138)** | **Conspiracies**  **(N=1262)** |
| --- | --- | --- | --- | --- | --- | --- |
| **Antipsychotics** | 0 | 879 (11.3%) | 430 (8.5%) | 467 (16.8%) | 326 (28.6%) | 465 (36.8%) |
| *Excluded* | 0 | 76 | 29 | 31 | 19 | 31 |
| **Antidepressants** | 36,200 (24.4%) | 3638 (46.6%) | 2206 (43.8%) | 1449 (52.2%) | 647 (56.9%) | 892 (70.1%) |
| *Excluded* | 5438 | 444 | 295 | 153 | 59 | 74 |
| **Anti-anxiety** | 19,621 (13.2%) | 2369 (30.4%) | 1376 (27.4%) | 981 (35.3%) | 450 (39.5%) | 710 (56.3%) |
| *Excluded* | 4069 | 484 | 283 | 153 | 64 | 97 |

Table 7: Medication use per PE type. Numbers include subjects that reported use of unprescribed medication (more than once) or prescribed medication (for at least two weeks). Excluded are subjects who reported use of alcohol or drugs as medication or who answer

# Supplementary Results

When performing all analyses while excluding participants who self-reported that they suffered from a form of psychotic disorder, the results did not significantly alter. Similarly, adding the use of medication for depression, anxiety or psychosis as a covariate to the model did not significantly impact on the associations between PEs and volumes or integrity. Cannabis was also tested as a mediator, but this did not show significant indirect or total effects (see supplementary material ST20-ST21).

## Grey matter cortical volumes

### Any PE

| Lobes | P-Value | FDR P-value | β-coefficient |
| --- | --- | --- | --- |
| Temporal Lobe | 0.316 | 0.749 | -0.006 |
| Occipital Lobe | 0.749 | 0.749 | -0.002 |
| Parietal Lobe | 0.676 | 0.749 | -0.003 |
| Frontal Lobe | 0.666 | 0.749 | -0.003 |

Table 37: Effect of any PE on volume per lobe

| B**rain Structure** | **P-values** | **FDR P-value** | β-coefficient |
| --- | --- | --- | --- |
| **Frontal Pole** | 0.857 | 0.959 | -0.005 |
| **Insular Cortex** | 0.236 | 0.959 | -0.039 |
| **Superior Frontal Gyrus** | 0.508 | 0.959 | -0.019 |
| **Middle Frontal Gyrus** | 0.596 | 0.959 | 0.016 |
| **Inferior Frontal Gyrus – pars triangularis** | 0.869 | 0.959 | -0.005 |
| **Inferior Frontal Gyrus – pars opercularis** | 0.993 | 0.993 | 0.000 |
| **Precentral Gyrus** | 0.415 | 0.959 | -0.025 |
| **Temporal Pole** | 0.988 | 0.993 | 0.000 |
| **Superior Temporal Gyrus – anterior division** | 0.911 | 0.972 | -0.003 |
| **Superior Temporal Gyrus – posterior division** | 0.030 | 0.474 | -0.057 |
| **Middle Temporal Gyrus – anterior division** | 0.494 | 0.959 | 0.020 |
| **Middle Temporal Gyrus – posterior division** | 0.425 | 0.959 | -0.024 |
| **Middle Temporal Gyrus – temporo-occipital part** | 0.633 | 0.959 | -0.012 |
| **Inferior Temporal Gyrus – anterior division** | 0.280 | 0.959 | 0.032 |
| **Inferior Temporal Gyrus – posterior division** | 0.478 | 0.959 | -0.022 |
| **Inferior Temporal Gyrus – temporo-occipital part** | 0.760 | 0.959 | -0.008 |
| **Postcentral Gyrus** | 0.170 | 0.959 | -0.041 |
| **Superior Parietal Lobule** | 0.461 | 0.959 | -0.021 |
| **Supramarginal Gyrus – anterior division** | 0.437 | 0.959 | -0.023 |
| **Supramarginal Gyrus – posterior division** | 0.728 | 0.959 | -0.009 |
| **Angular Gyrus** | 0.450 | 0.959 | 0.018 |
| **Lateral Occipital Cortex – superior division** | 0.561 | 0.959 | -0.017 |
| **Lateral Occipital Cortex – inferior division** | 0.829 | 0.959 | -0.006 |
| **Intracalcarine Cortex** | 0.597 | 0.959 | 0.018 |
| **Frontal Medial Cortex** | 0.673 | 0.959 | -0.014 |
| **Juxtapositional Lobule Cortex** | 0.213 | 0.959 | 0.042 |
| **Subcallosal Cortex** | 0.699 | 0.959 | -0.012 |
| **Paracingulate Gyrus** | 0.575 | 0.959 | -0.018 |
| **Cingulate Gyrus – anterior division** | 0.204 | 0.959 | 0.042 |
| **Cingulate Gyrus – posterior division** | 0.362 | 0.959 | 0.029 |
| **Precuneous Cortex** | 0.291 | 0.959 | 0.034 |
| **Cuneal Cortex** | 0.672 | 0.959 | 0.012 |
| **Frontal Orbital Cortex** | 0.040 | 0.474 | -0.059 |
| **Parahippocampal Gyrus – anterior division** | 0.781 | 0.959 | 0.009 |
| **Parahippocampal Gyrus – posterior division** | 0.963 | 0.993 | 0.001 |
| **Lingual Gyrus** | 0.879 | 0.959 | 0.004 |
| **Temporal Fusiform Cortex – anterior division** | 0.615 | 0.959 | -0.015 |
| **Temporal Fusiform Cortex – posterior division** | 0.310 | 0.959 | -0.026 |
| **Temporal Occipital Fusiform Cortex** | 0.851 | 0.959 | -0.005 |
| **Occipital Fusiform Cortex** | 0.296 | 0.959 | -0.030 |
| **Frontal Operculum Cortex** | 0.559 | 0.959 | -0.016 |
| **Central Opercular Cortex** | 0.667 | 0.959 | -0.013 |
| **Parietal Operculum Cortex** | 0.003 | 0.148 | -0.088 |
| **Planum Polare** | 0.851 | 0.959 | -0.006 |
| **Heschl’s Gyrus** | 0.116 | 0.959 | -0.045 |
| **Planum Temporale** | 0.006 | 0.148 | -0.071 |
| **Supracalcarine Cortex** | 0.571 | 0.959 | 0.015 |
| **Occipital Pole** | 0.721 | 0.959 | -0.011 |

Table 38: Effect of any PE on cortical structures

| B**rain Structure** | **P-values** | **FDR P-value** | **β-coefficient** |
| --- | --- | --- | --- |
| **Frontal Pole** | 0.289 | 0.920 | -0.021 |
| **Insular Cortex** | 0.766 | 0.920 | -0.005 |
| **Superior Frontal Gyrus** | 0.736 | 0.920 | 0.010 |
| **Middle Frontal Gyrus** | 0.613 | 0.920 | -0.016 |
| **Inferior Frontal Gyrus – pars triangularis** | 0.217 | 0.920 | -0.053 |
| **Inferior Frontal Gyrus – pars opercularis** | 0.832 | 0.929 | -0.009 |
| **Precentral Gyrus** | 0.748 | 0.920 | -0.009 |
| **Temporal Pole** | 0.803 | 0.928 | 0.007 |
| **Superior Temporal Gyrus – anterior division** | 0.362 | 0.920 | -0.036 |
| **Superior Temporal Gyrus – posterior division** | 0.460 | 0.920 | 0.025 |
| **Middle Temporal Gyrus – anterior division** | 0.636 | 0.920 | -0.017 |
| **Middle Temporal Gyrus – posterior division** | 0.192 | 0.920 | 0.045 |
| **Middle Temporal Gyrus – temporo-occipital part** | 0.812 | 0.928 | 0.008 |
| **Inferior Temporal Gyrus – anterior division** | 0.652 | 0.920 | 0.018 |
| **Inferior Temporal Gyrus – posterior division** | 0.568 | 0.920 | 0.021 |
| **Inferior Temporal Gyrus – temporo-occipital part** | 0.639 | 0.920 | -0.016 |
| **Postcentral Gyrus** | 0.654 | 0.920 | -0.014 |
| **Superior Parietal Lobule** | 0.044 | 0.482 | -0.081 |
| **Supramarginal Gyrus – anterior division** | 0.699 | 0.920 | 0.017 |
| **Supramarginal Gyrus – posterior division** | 0.876 | 0.949 | 0.006 |
| **Angular Gyrus** | 0.739 | 0.920 | 0.012 |
| **Lateral Occipital Cortex – superior division** | 0.540 | 0.920 | 0.021 |
| **Lateral Occipital Cortex – inferior division** | 0.378 | 0.920 | -0.034 |
| **Intracalcarine Cortex** | 0.458 | 0.920 | 0.015 |
| **Frontal Medial Cortex** | 0.287 | 0.920 | -0.028 |
| **Juxtapositional Lobule Cortex** | 0.565 | 0.920 | -0.015 |
| **Subcallosal Cortex** | 0.890 | 0.949 | 0.002 |
| **Paracingulate Gyrus** | 0.060 | 0.482 | 0.046 |
| **Cingulate Gyrus – anterior division** | 0.550 | 0.920 | -0.014 |
| **Cingulate Gyrus – posterior division** | 0.265 | 0.920 | 0.020 |
| **Precuneous Cortex** | 0.721 | 0.920 | 0.007 |
| **Cuneal Cortex** | 0.662 | 0.920 | 0.013 |
| **Frontal Orbital Cortex** | 0.001 | 0.044 | 0.083 |
| **Parahippocampal Gyrus – anterior division** | 0.130 | 0.889 | -0.041 |
| **Parahippocampal Gyrus – posterior division** | 0.652 | 0.920 | 0.011 |
| **Lingual Gyrus** | 0.667 | 0.920 | -0.011 |
| **Temporal Fusiform Cortex – anterior division** | 0.917 | 0.956 | -0.004 |
| **Temporal Fusiform Cortex – posterior division** | 0.504 | 0.920 | 0.016 |
| **Temporal Occipital Fusiform Cortex** | 0.749 | 0.920 | -0.010 |
| **Occipital Fusiform Cortex** | 0.014 | 0.324 | 0.092 |
| **Frontal Operculum Cortex** | 0.972 | 0.992 | -0.001 |
| **Central Opercular Cortex** | 0.609 | 0.920 | -0.016 |
| **Parietal Operculum Cortex** | 0.022 | 0.344 | 0.082 |
| **Planum Polare** | 0.992 | 0.992 | 0.000 |
| **Heschl’s Gyrus** | 0.509 | 0.920 | 0.021 |
| **Planum Temporale** | 0.057 | 0.482 | 0.060 |
| **Supracalcarine Cortex** | 0.338 | 0.920 | -0.023 |
| **Occipital Pole** | 0.385 | 0.920 | -0.027 |

Table 39: Interaction effect between hemisphere and any PE on cortical volumes

| **Brain Structure** | **P-values** | **FDR P-value** | **β-coefficient** |
| --- | --- | --- | --- |
| **Frontal Pole** | 0.233 | 0.856 | -0.064 |
| **Insular Cortex** | 0.409 | 0.856 | -0.055 |
| **Superior Frontal Gyrus** | 0.723 | 0.856 | 0.021 |
| **Middle Frontal Gyrus** | 0.279 | 0.856 | -0.069 |
| **Inferior Frontal Gyrus – pars triangularis** | 0.609 | 0.856 | -0.030 |
| **Inferior Frontal Gyrus – pars opercularis** | 0.427 | 0.856 | -0.046 |
| **Precentral Gyrus** | 0.046 | 0.773 | -0.125 |
| **Temporal Pole** | 0.598 | 0.856 | -0.032 |
| **Superior Temporal Gyrus – anterior division** | 0.763 | 0.856 | 0.018 |
| **Superior Temporal Gyrus – posterior division** | 0.601 | 0.856 | -0.028 |
| **Middle Temporal Gyrus – anterior division** | 0.709 | 0.856 | 0.022 |
| **Middle Temporal Gyrus – posterior division** | 0.557 | 0.856 | 0.036 |
| **Middle Temporal Gyrus – temporo-occipital part** | 0.602 | 0.856 | -0.026 |
| **Inferior Temporal Gyrus – anterior division** | 0.720 | 0.856 | -0.022 |
| **Inferior Temporal Gyrus – posterior division** | 0.471 | 0.856 | 0.045 |
| **Inferior Temporal Gyrus – temporo-occipital part** | 0.048 | 0.773 | -0.102 |
| **Postcentral Gyrus** | 0.146 | 0.856 | -0.088 |
| **Superior Parietal Lobule** | 0.310 | 0.856 | 0.060 |
| **Supramarginal Gyrus – anterior division** | 0.040 | 0.773 | -0.125 |
| **Supramarginal Gyrus – posterior division** | 0.767 | 0.856 | 0.016 |
| **Angular Gyrus** | 0.726 | 0.856 | -0.017 |
| **Lateral Occipital Cortex – superior division** | 0.979 | 0.996 | -0.002 |
| **Lateral Occipital Cortex – inferior division** | 0.372 | 0.856 | -0.052 |
| **Intracalcarine Cortex** | 0.424 | 0.856 | 0.055 |
| **Frontal Medial Cortex** | 0.467 | 0.856 | -0.049 |
| **Juxtapositional Lobule Cortex** | 0.474 | 0.856 | 0.049 |
| **Subcallosal Cortex** | 0.125 | 0.856 | -0.099 |
| **Paracingulate Gyrus** | 0.996 | 0.996 | 0.000 |
| **Cingulate Gyrus – anterior division** | 0.390 | 0.856 | -0.058 |
| **Cingulate Gyrus – posterior division** | 0.435 | 0.856 | -0.050 |
| **Precuneous Cortex** | 0.875 | 0.933 | -0.010 |
| **Cuneal Cortex** | 0.268 | 0.856 | 0.066 |
| **Frontal Orbital Cortex** | 0.789 | 0.861 | -0.015 |
| **Parahippocampal Gyrus – anterior division** | 0.154 | 0.856 | 0.090 |
| **Parahippocampal Gyrus – posterior division** | 0.731 | 0.856 | 0.017 |
| **Lingual Gyrus** | 0.281 | 0.856 | 0.063 |
| **Temporal Fusiform Cortex – anterior division** | 0.950 | 0.991 | -0.004 |
| **Temporal Fusiform Cortex – posterior division** | 0.518 | 0.856 | 0.034 |
| **Temporal Occipital Fusiform Cortex** | 0.674 | 0.856 | 0.021 |
| **Occipital Fusiform Cortex** | 0.717 | 0.856 | -0.021 |
| **Frontal Operculum Cortex** | 0.188 | 0.856 | -0.075 |
| **Central Opercular Cortex** | 0.521 | 0.856 | -0.040 |
| **Parietal Operculum Cortex** | 0.239 | 0.856 | -0.072 |
| **Planum Polare** | 0.503 | 0.856 | -0.041 |
| **Heschl’s Gyrus** | 0.674 | 0.856 | 0.024 |
| **Planum Temporale** | 0.662 | 0.856 | -0.023 |
| **Supracalcarine Cortex** | 0.567 | 0.856 | -0.030 |
| **Occipital Pole** | 0.591 | 0.856 | -0.033 |

Table 40: Interaction effect between sex and any PE on cortical volumes

### Visions

| Lobes | P-Value | FDR P-value | β-coefficient |
| --- | --- | --- | --- |
| Temporal Lobe | 0.614 | 0.963 | -0.003 |
| Occipital Lobe | 0.963 | 0.963 | 0.000 |
| Parietal Lobe | 0.748 | 0.963 | -0.002 |
| Frontal Lobe | 0.889 | 0.963 | -0.001 |

Table 41: Effect of 'Visions' on volume per lobe

| Brain Structure | P-values | FDR P-value | β-coefficient |
| --- | --- | --- | --- |
| Frontal Pole | 0.789 | 0.980 | 0.009 |
| Insular Cortex | 0.298 | 0.980 | -0.043 |
| Superior Frontal Gyrus | 0.761 | 0.980 | -0.011 |
| Middle Frontal Gyrus | 0.964 | 0.980 | 0.002 |
| Inferior Frontal Gyrus – pars triangularis | 0.575 | 0.980 | 0.020 |
| Inferior Frontal Gyrus – pars opercularis | 0.214 | 0.980 | 0.044 |
| Precentral Gyrus | 0.966 | 0.980 | -0.002 |
| Temporal Pole | 0.511 | 0.980 | 0.025 |
| Superior Temporal Gyrus – anterior division | 0.828 | 0.980 | 0.008 |
| Superior Temporal Gyrus – posterior division | 0.204 | 0.980 | -0.042 |
| Middle Temporal Gyrus – anterior division | 0.745 | 0.980 | 0.012 |
| Middle Temporal Gyrus – posterior division | 0.263 | 0.980 | -0.043 |
| Middle Temporal Gyrus – temporo-occipital part | 0.886 | 0.980 | 0.004 |
| Inferior Temporal Gyrus – anterior division | 0.770 | 0.980 | 0.011 |
| Inferior Temporal Gyrus – posterior division | 0.327 | 0.980 | -0.038 |
| Inferior Temporal Gyrus – temporo-occipital part | 0.353 | 0.980 | 0.029 |
| Postcentral Gyrus | 0.435 | 0.980 | -0.029 |
| Superior Parietal Lobule | 0.522 | 0.980 | -0.023 |
| Supramarginal Gyrus – anterior division | 0.368 | 0.980 | -0.034 |
| Supramarginal Gyrus – posterior division | 0.497 | 0.980 | -0.022 |
| Angular Gyrus | 0.421 | 0.980 | 0.024 |
| Lateral Occipital Cortex – superior division | 0.898 | 0.980 | -0.005 |
| Lateral Occipital Cortex – inferior division | 0.976 | 0.980 | 0.001 |
| Intracalcarine Cortex | 0.881 | 0.980 | 0.006 |
| Frontal Medial Cortex | 0.537 | 0.980 | -0.026 |
| Juxtapositional Lobule Cortex | 0.157 | 0.980 | 0.060 |
| Subcallosal Cortex | 0.962 | 0.980 | 0.002 |
| Paracingulate Gyrus | 0.587 | 0.980 | -0.022 |
| Cingulate Gyrus – anterior division | 0.728 | 0.980 | 0.014 |
| Cingulate Gyrus – posterior division | 0.273 | 0.980 | 0.043 |
| Precuneous Cortex | 0.527 | 0.980 | 0.025 |
| Cuneal Cortex | 0.604 | 0.980 | 0.019 |
| Frontal Orbital Cortex | 0.122 | 0.980 | -0.055 |
| Parahippocampal Gyrus – anterior division | 0.434 | 0.980 | 0.031 |
| Parahippocampal Gyrus – posterior division | 0.743 | 0.980 | 0.010 |
| Lingual Gyrus | 0.980 | 0.980 | 0.001 |
| Temporal Fusiform Cortex – anterior division | 0.882 | 0.980 | -0.005 |
| Temporal Fusiform Cortex – posterior division | 0.322 | 0.980 | -0.032 |
| Temporal Occipital Fusiform Cortex | 0.872 | 0.980 | -0.005 |
| Occipital Fusiform Cortex | 0.624 | 0.980 | -0.018 |
| Frontal Operculum Cortex | 0.908 | 0.980 | -0.004 |
| Central Opercular Cortex | 0.860 | 0.980 | -0.007 |
| Parietal Operculum Cortex | 0.033 | 0.980 | -0.080 |
| Planum Polare | 0.796 | 0.980 | -0.010 |
| Heschl’s Gyrus | 0.511 | 0.980 | -0.023 |
| Planum Temporale | 0.094 | 0.980 | -0.054 |
| Supracalcarine Cortex | 0.428 | 0.980 | 0.026 |
| Occipital Pole | 0.865 | 0.980 | 0.006 |

Table 42: Effect of ‘Visions’ PE on cortical volumes

| Brain Structure | P-values | FDR P-value | β-coefficient |
| --- | --- | --- | --- |
| Frontal Pole | 0.534 | 0.931 | -0.016 |
| Insular Cortex | 0.684 | 0.931 | 0.008 |
| Superior Frontal Gyrus | 0.182 | 0.794 | 0.048 |
| Middle Frontal Gyrus | 0.336 | 0.897 | -0.038 |
| Inferior Frontal Gyrus – pars triangularis | 0.651 | 0.931 | -0.024 |
| Inferior Frontal Gyrus – pars opercularis | 0.794 | 0.977 | -0.014 |
| Precentral Gyrus | 0.591 | 0.931 | -0.018 |
| Temporal Pole | 0.109 | 0.748 | 0.058 |
| Superior Temporal Gyrus – anterior division | 0.312 | 0.897 | -0.051 |
| Superior Temporal Gyrus – posterior division | 0.977 | 0.990 | 0.001 |
| Middle Temporal Gyrus – anterior division | 0.890 | 0.990 | -0.006 |
| Middle Temporal Gyrus – posterior division | 0.286 | 0.897 | 0.046 |
| Middle Temporal Gyrus – temporo-occipital part | 0.360 | 0.911 | -0.039 |
| Inferior Temporal Gyrus – anterior division | 0.269 | 0.897 | 0.055 |
| Inferior Temporal Gyrus – posterior division | 0.489 | 0.931 | 0.031 |
| Inferior Temporal Gyrus – temporo-occipital part | 0.657 | 0.931 | -0.018 |
| Postcentral Gyrus | 0.109 | 0.748 | -0.065 |
| Superior Parietal Lobule | 0.175 | 0.794 | -0.069 |
| Supramarginal Gyrus – anterior division | 0.818 | 0.981 | -0.012 |
| Supramarginal Gyrus – posterior division | 0.883 | 0.990 | -0.007 |
| Angular Gyrus | 0.990 | 0.990 | 0.001 |
| Lateral Occipital Cortex – superior division | 0.294 | 0.897 | 0.046 |
| Lateral Occipital Cortex – inferior division | 0.558 | 0.931 | -0.028 |
| Intracalcarine Cortex | 0.717 | 0.931 | 0.009 |
| Frontal Medial Cortex | 0.125 | 0.748 | -0.050 |
| Juxtapositional Lobule Cortex | 0.738 | 0.932 | 0.011 |
| Subcallosal Cortex | 0.322 | 0.897 | 0.020 |
| Paracingulate Gyrus | 0.173 | 0.794 | 0.042 |
| Cingulate Gyrus – anterior division | 0.464 | 0.931 | -0.022 |
| Cingulate Gyrus – posterior division | 0.517 | 0.931 | 0.014 |
| Precuneous Cortex | 0.570 | 0.931 | 0.014 |
| Cuneal Cortex | 0.681 | 0.931 | -0.015 |
| Frontal Orbital Cortex | 0.112 | 0.748 | 0.050 |
| Parahippocampal Gyrus – anterior division | 0.453 | 0.931 | -0.025 |
| Parahippocampal Gyrus – posterior division | 0.949 | 0.990 | -0.002 |
| Lingual Gyrus | 0.516 | 0.931 | 0.021 |
| Temporal Fusiform Cortex – anterior division | 0.916 | 0.990 | 0.004 |
| Temporal Fusiform Cortex – posterior division | 0.230 | 0.897 | 0.036 |
| Temporal Occipital Fusiform Cortex | 0.978 | 0.990 | -0.001 |
| Occipital Fusiform Cortex | 0.013 | 0.624 | 0.116 |
| Frontal Operculum Cortex | 0.683 | 0.931 | 0.018 |
| Central Opercular Cortex | 0.488 | 0.931 | -0.027 |
| Parietal Operculum Cortex | 0.055 | 0.748 | 0.086 |
| Planum Polare | 0.705 | 0.931 | 0.014 |
| Heschl’s Gyrus | 0.502 | 0.931 | 0.026 |
| Planum Temporale | 0.122 | 0.748 | 0.061 |
| Supracalcarine Cortex | 0.935 | 0.990 | 0.002 |
| Occipital Pole | 0.098 | 0.748 | -0.064 |

Table 43: Interaction effect of hemisphere and ‘Visions’ PE on cortical volumes

| Brain Structure | P-values | FDR P-value | β-coefficient |
| --- | --- | --- | --- |
| Frontal Pole | 0.030 | 0.354 | -0.152 |
| Insular Cortex | 0.061 | 0.486 | -0.162 |
| Superior Frontal Gyrus | 0.763 | 0.872 | 0.023 |
| Middle Frontal Gyrus | 0.153 | 0.609 | -0.117 |
| Inferior Frontal Gyrus – pars triangularis | 0.468 | 0.796 | -0.055 |
| Inferior Frontal Gyrus – pars opercularis | 0.838 | 0.924 | -0.015 |
| Precentral Gyrus | 0.021 | 0.354 | -0.188 |
| Temporal Pole | 0.177 | 0.609 | -0.107 |
| Superior Temporal Gyrus – anterior division | 0.602 | 0.811 | 0.041 |
| Superior Temporal Gyrus – posterior division | 0.423 | 0.796 | -0.056 |
| Middle Temporal Gyrus – anterior division | 0.874 | 0.924 | -0.012 |
| Middle Temporal Gyrus – posterior division | 0.590 | 0.811 | -0.043 |
| Middle Temporal Gyrus – temporo-occipital part | 0.276 | 0.747 | -0.069 |
| Inferior Temporal Gyrus – anterior division | 0.906 | 0.926 | -0.009 |
| Inferior Temporal Gyrus – posterior division | 0.488 | 0.796 | -0.056 |
| Inferior Temporal Gyrus – temporo-occipital part | 0.073 | 0.499 | -0.120 |
| Postcentral Gyrus | 0.085 | 0.511 | -0.135 |
| Superior Parietal Lobule | 0.467 | 0.796 | -0.056 |
| Supramarginal Gyrus – anterior division | 0.022 | 0.354 | -0.181 |
| Supramarginal Gyrus – posterior division | 0.852 | 0.924 | 0.013 |
| Angular Gyrus | 0.651 | 0.822 | -0.028 |
| Lateral Occipital Cortex – superior division | 0.599 | 0.811 | -0.041 |
| Lateral Occipital Cortex – inferior division | 0.203 | 0.650 | -0.096 |
| Intracalcarine Cortex | 0.311 | 0.747 | 0.090 |
| Frontal Medial Cortex | 0.396 | 0.796 | -0.074 |
| Juxtapositional Lobule Cortex | 0.472 | 0.796 | -0.064 |
| Subcallosal Cortex | 0.014 | 0.354 | -0.208 |
| Paracingulate Gyrus | 0.763 | 0.872 | -0.025 |
| Cingulate Gyrus – anterior division | 0.055 | 0.486 | -0.167 |
| Cingulate Gyrus – posterior division | 0.150 | 0.609 | -0.120 |
| Precuneous Cortex | 0.885 | 0.924 | -0.012 |
| Cuneal Cortex | 0.531 | 0.811 | 0.048 |
| Frontal Orbital Cortex | 0.178 | 0.609 | -0.101 |
| Parahippocampal Gyrus – anterior division | 0.625 | 0.811 | 0.040 |
| Parahippocampal Gyrus – posterior division | 0.497 | 0.796 | 0.044 |
| Lingual Gyrus | 0.618 | 0.811 | 0.038 |
| Temporal Fusiform Cortex – anterior division | 0.440 | 0.796 | -0.059 |
| Temporal Fusiform Cortex – posterior division | 0.429 | 0.796 | -0.053 |
| Temporal Occipital Fusiform Cortex | 0.615 | 0.811 | -0.033 |
| Occipital Fusiform Cortex | 0.965 | 0.965 | 0.003 |
| Frontal Operculum Cortex | 0.271 | 0.747 | -0.082 |
| Central Opercular Cortex | 0.304 | 0.747 | -0.082 |
| Parietal Operculum Cortex | 0.101 | 0.537 | -0.130 |
| Planum Polare | 0.146 | 0.609 | -0.116 |
| Heschl’s Gyrus | 0.399 | 0.796 | -0.063 |
| Planum Temporale | 0.291 | 0.747 | -0.072 |
| Supracalcarine Cortex | 0.741 | 0.872 | 0.023 |
| Occipital Pole | 0.763 | 0.872 | -0.024 |

Table 44: Interaction effect between sex and ‘Visions’ PE on cortical volumes

### Voices

| Lobes | P-Value | FDR P-value | β-coefficient |
| --- | --- | --- | --- |
| Temporal Lobe * | 0.009 | 0.047 | -0.017 |
| Occipital Lobe | 0.043 | 0.108 | -0.013 |
| Parietal Lobe | 0.245 | 0.245 | -0.008 |
| Frontal Lobe | 0.076 | 0.114 | -0.012 |

Table 45: Effect of 'Voices' on volume per lobe

| Brain Structure | P-values | FDR P-value | β-coefficient |
| --- | --- | --- | --- |
| Frontal Pole | 0.194 | 0.376 | -0.055 |
| Insular Cortex | 0.113 | 0.353 | -0.083 |
| Superior Frontal Gyrus | 0.204 | 0.376 | -0.060 |
| Middle Frontal Gyrus | 0.511 | 0.628 | -0.033 |
| Inferior Frontal Gyrus – pars triangularis | 0.696 | 0.761 | 0.018 |
| Inferior Frontal Gyrus – pars opercularis | 0.221 | 0.392 | -0.056 |
| Precentral Gyrus | 0.017 | 0.195 | -0.119 |
| Temporal Pole | 0.840 | 0.876 | -0.010 |
| Superior Temporal Gyrus – anterior division | 0.434 | 0.579 | -0.038 |
| Superior Temporal Gyrus – posterior division | 0.045 | 0.214 | -0.085 |
| Middle Temporal Gyrus – anterior division | 0.531 | 0.633 | -0.029 |
| Middle Temporal Gyrus – posterior division | 0.062 | 0.247 | -0.092 |
| Middle Temporal Gyrus – temporo-occipital part | 0.146 | 0.376 | -0.056 |
| Inferior Temporal Gyrus – anterior division | 0.976 | 0.976 | -0.001 |
| Inferior Temporal Gyrus – posterior division | 0.008 | 0.152 | -0.132 |
| Inferior Temporal Gyrus – temporo-occipital part | 0.112 | 0.353 | -0.065 |
| Postcentral Gyrus | 0.172 | 0.376 | -0.065 |
| Superior Parietal Lobule | 0.254 | 0.407 | -0.053 |
| Supramarginal Gyrus – anterior division | 0.168 | 0.376 | -0.067 |
| Supramarginal Gyrus – posterior division | 0.406 | 0.556 | -0.035 |
| Angular Gyrus | 0.398 | 0.556 | -0.032 |
| Lateral Occipital Cortex – superior division | 0.024 | 0.195 | -0.108 |
| Lateral Occipital Cortex – inferior division | 0.118 | 0.353 | -0.072 |
| Intracalcarine Cortex | 0.816 | 0.870 | 0.013 |
| Frontal Medial Cortex | 0.466 | 0.588 | -0.039 |
| Juxtapositional Lobule Cortex | 0.698 | 0.761 | 0.021 |
| Subcallosal Cortex | 0.183 | 0.376 | -0.068 |
| Paracingulate Gyrus | 0.159 | 0.376 | -0.073 |
| Cingulate Gyrus – anterior division | 0.541 | 0.633 | 0.033 |
| Cingulate Gyrus – posterior division | 0.392 | 0.556 | -0.044 |
| Precuneous Cortex | 0.665 | 0.760 | 0.022 |
| Cuneal Cortex | 0.145 | 0.376 | -0.069 |
| Frontal Orbital Cortex | 0.030 | 0.195 | -0.100 |
| Parahippocampal Gyrus – anterior division | 0.179 | 0.376 | -0.068 |
| Parahippocampal Gyrus – posterior division | 0.340 | 0.527 | -0.038 |
| Lingual Gyrus | 0.245 | 0.405 | -0.054 |
| Temporal Fusiform Cortex – anterior division | 0.202 | 0.376 | -0.060 |
| Temporal Fusiform Cortex – posterior division | 0.033 | 0.195 | -0.088 |
| Temporal Occipital Fusiform Cortex | 0.083 | 0.306 | -0.069 |
| Occipital Fusiform Cortex | 0.034 | 0.195 | -0.098 |
| Frontal Operculum Cortex | 0.466 | 0.588 | -0.033 |
| Central Opercular Cortex | 0.055 | 0.238 | -0.094 |
| Parietal Operculum Cortex | 0.004 | 0.152 | -0.139 |
| Planum Polare | 0.394 | 0.556 | -0.042 |
| Heschl’s Gyrus | 0.037 | 0.195 | -0.096 |
| Planum Temporale | 0.009 | 0.152 | -0.108 |
| Supracalcarine Cortex | 0.877 | 0.896 | -0.006 |
| Occipital Pole | 0.239 | 0.405 | -0.057 |

| Brain Structure | P-values | FDR P-value | β-coefficient |
| --- | --- | --- | --- |
| Frontal Pole | 0.485 | 0.961 | 0.022 |
| Insular Cortex | 0.286 | 0.961 | -0.028 |
| Superior Frontal Gyrus | 0.952 | 0.970 | 0.003 |
| Middle Frontal Gyrus | 0.499 | 0.961 | 0.035 |
| Inferior Frontal Gyrus – pars triangularis | 0.267 | 0.961 | -0.076 |
| Inferior Frontal Gyrus – pars opercularis | 0.351 | 0.961 | -0.064 |
| Precentral Gyrus | 0.032 | 0.593 | -0.094 |
| Temporal Pole | 0.173 | 0.961 | -0.063 |
| Superior Temporal Gyrus – anterior division | 0.554 | 0.970 | -0.038 |
| Superior Temporal Gyrus – posterior division | 0.220 | 0.961 | 0.068 |
| Middle Temporal Gyrus – anterior division | 0.798 | 0.970 | -0.015 |
| Middle Temporal Gyrus – posterior division | 0.713 | 0.970 | 0.021 |
| Middle Temporal Gyrus – temporo-occipital part | 0.932 | 0.970 | -0.005 |
| Inferior Temporal Gyrus – anterior division | 0.890 | 0.970 | -0.009 |
| Inferior Temporal Gyrus – posterior division | 0.824 | 0.970 | -0.013 |
| Inferior Temporal Gyrus – temporo-occipital part | 0.942 | 0.970 | -0.004 |
| Postcentral Gyrus | 0.420 | 0.961 | -0.042 |
| Superior Parietal Lobule | 0.637 | 0.970 | -0.031 |
| Supramarginal Gyrus – anterior division | 0.494 | 0.961 | 0.048 |
| Supramarginal Gyrus – posterior division | 0.178 | 0.961 | 0.088 |
| Angular Gyrus | 0.776 | 0.970 | 0.016 |
| Lateral Occipital Cortex – superior division | 0.745 | 0.970 | -0.018 |
| Lateral Occipital Cortex – inferior division | 0.792 | 0.970 | 0.016 |
| Intracalcarine Cortex | 0.469 | 0.961 | 0.024 |
| Frontal Medial Cortex | 0.260 | 0.961 | 0.047 |
| Juxtapositional Lobule Cortex | 0.304 | 0.961 | -0.042 |
| Subcallosal Cortex | 0.399 | 0.961 | -0.022 |
| Paracingulate Gyrus | 0.179 | 0.961 | 0.053 |
| Cingulate Gyrus – anterior division | 0.550 | 0.970 | -0.023 |
| Cingulate Gyrus – posterior division | 0.933 | 0.970 | 0.002 |
| Precuneous Cortex | 0.776 | 0.970 | -0.009 |
| Cuneal Cortex | 0.617 | 0.970 | -0.024 |
| Frontal Orbital Cortex | 0.437 | 0.961 | 0.031 |
| Parahippocampal Gyrus – anterior division | 0.158 | 0.961 | -0.062 |
| Parahippocampal Gyrus – posterior division | 0.495 | 0.961 | 0.027 |
| Lingual Gyrus | 0.966 | 0.970 | 0.002 |
| Temporal Fusiform Cortex – anterior division | 0.769 | 0.970 | 0.016 |
| Temporal Fusiform Cortex – posterior division | 0.487 | 0.961 | 0.027 |
| Temporal Occipital Fusiform Cortex | 0.767 | 0.970 | -0.014 |
| Occipital Fusiform Cortex | 0.838 | 0.970 | 0.012 |
| Frontal Operculum Cortex | 0.970 | 0.970 | 0.002 |
| Central Opercular Cortex | 0.036 | 0.593 | -0.104 |
| Parietal Operculum Cortex | 0.501 | 0.961 | 0.039 |
| Planum Polare | 0.610 | 0.970 | -0.024 |
| Heschl’s Gyrus | 0.772 | 0.970 | -0.015 |
| Planum Temporale | 0.399 | 0.961 | 0.043 |
| Supracalcarine Cortex | 0.037 | 0.593 | -0.081 |
| Occipital Pole | 0.168 | 0.961 | -0.069 |

| Brain Structure | P-values | FDR P-value | β-coefficient |
| --- | --- | --- | --- |
| Frontal Pole | 0.886 | 0.910 | -0.012 |
| Insular Cortex | 0.279 | 0.910 | 0.115 |
| Superior Frontal Gyrus | 0.381 | 0.910 | 0.084 |
| Middle Frontal Gyrus | 0.526 | 0.910 | -0.064 |
| Inferior Frontal Gyrus – pars triangularis | 0.712 | 0.910 | -0.035 |
| Inferior Frontal Gyrus – pars opercularis | 0.512 | 0.910 | -0.061 |
| Precentral Gyrus | 0.388 | 0.910 | -0.087 |
| Temporal Pole | 0.741 | 0.910 | -0.032 |
| Superior Temporal Gyrus – anterior division | 0.733 | 0.910 | 0.033 |
| Superior Temporal Gyrus – posterior division | 0.862 | 0.910 | 0.015 |
| Middle Temporal Gyrus – anterior division | 0.498 | 0.910 | -0.063 |
| Middle Temporal Gyrus – posterior division | 0.203 | 0.910 | 0.126 |
| Middle Temporal Gyrus – temporo-occipital part | 0.885 | 0.910 | -0.011 |
| Inferior Temporal Gyrus – anterior division | 0.178 | 0.910 | -0.129 |
| Inferior Temporal Gyrus – posterior division | 0.811 | 0.910 | 0.024 |
| Inferior Temporal Gyrus – temporo-occipital part | 0.188 | 0.910 | -0.109 |
| Postcentral Gyrus | 0.832 | 0.910 | -0.021 |
| Superior Parietal Lobule | 0.421 | 0.910 | 0.076 |
| Supramarginal Gyrus – anterior division | 0.085 | 0.910 | -0.168 |
| Supramarginal Gyrus – posterior division | 0.605 | 0.910 | -0.044 |
| Angular Gyrus | 0.682 | 0.910 | -0.031 |
| Lateral Occipital Cortex – superior division | 0.918 | 0.918 | -0.010 |
| Lateral Occipital Cortex – inferior division | 0.891 | 0.910 | 0.013 |
| Intracalcarine Cortex | 0.879 | 0.910 | -0.017 |
| Frontal Medial Cortex | 0.428 | 0.910 | -0.086 |
| Juxtapositional Lobule Cortex | 0.234 | 0.910 | 0.131 |
| Subcallosal Cortex | 0.826 | 0.910 | -0.023 |
| Paracingulate Gyrus | 0.747 | 0.910 | 0.034 |
| Cingulate Gyrus – anterior division | 0.266 | 0.910 | 0.120 |
| Cingulate Gyrus – posterior division | 0.820 | 0.910 | 0.023 |
| Precuneous Cortex | 0.436 | 0.910 | -0.081 |
| Cuneal Cortex | 0.386 | 0.910 | 0.083 |
| Frontal Orbital Cortex | 0.561 | 0.910 | -0.054 |
| Parahippocampal Gyrus – anterior division | 0.459 | 0.910 | 0.075 |
| Parahippocampal Gyrus – posterior division | 0.141 | 0.910 | 0.119 |
| Lingual Gyrus | 0.120 | 0.910 | 0.145 |
| Temporal Fusiform Cortex – anterior division | 0.389 | 0.910 | -0.081 |
| Temporal Fusiform Cortex – posterior division | 0.694 | 0.910 | 0.033 |
| Temporal Occipital Fusiform Cortex | 0.794 | 0.910 | 0.021 |
| Occipital Fusiform Cortex | 0.203 | 0.910 | -0.118 |
| Frontal Operculum Cortex | 0.580 | 0.910 | -0.051 |
| Central Opercular Cortex | 0.672 | 0.910 | -0.042 |
| Parietal Operculum Cortex | 0.192 | 0.910 | -0.127 |
| Planum Polare | 0.596 | 0.910 | 0.052 |
| Heschl’s Gyrus | 0.608 | 0.910 | 0.047 |
| Planum Temporale | 0.455 | 0.910 | -0.063 |
| Supracalcarine Cortex | 0.315 | 0.910 | -0.085 |
| Occipital Pole | 0.849 | 0.910 | -0.019 |

### Communications

| Brain Structure | P-values | FDR P-value | β-coefficient |
| --- | --- | --- | --- |
| Temporal Lobe | 0.993 | 0.993 | 0.000 |
| Occipital Lobe | 0.712 | 0.890 | -0.002 |
| Parietal Lobe | 0.621 | 0.890 | -0.003 |
| Frontal Lobe | 0.516 | 0.890 | 0.004 |

Table 46: Effect of 'Communications' on volume per lobe

| Brain Structure | P-values | FDR P-value | β-coefficient |
| --- | --- | --- | --- |
| Frontal Pole | 0.564 | 0.998 | 0.039 |
| Insular Cortex | 0.808 | 0.998 | -0.020 |
| Superior Frontal Gyrus | 0.421 | 0.998 | 0.060 |
| Middle Frontal Gyrus | 0.063 | 0.998 | 0.147 |
| Inferior Frontal Gyrus – pars triangularis | 0.944 | 0.998 | -0.005 |
| Inferior Frontal Gyrus – pars opercularis | 0.761 | 0.998 | -0.022 |
| Precentral Gyrus | 0.995 | 0.998 | 0.001 |
| Temporal Pole | 0.749 | 0.998 | 0.025 |
| Superior Temporal Gyrus – anterior division | 0.891 | 0.998 | 0.010 |
| Superior Temporal Gyrus – posterior division | 0.430 | 0.998 | -0.053 |
| Middle Temporal Gyrus – anterior division | 0.272 | 0.998 | 0.080 |
| Middle Temporal Gyrus – posterior division | 0.768 | 0.998 | 0.023 |
| Middle Temporal Gyrus – temporo-occipital part | 0.998 | 0.998 | 0.000 |
| Inferior Temporal Gyrus – anterior division | 0.514 | 0.998 | 0.049 |
| Inferior Temporal Gyrus – posterior division | 0.528 | 0.998 | 0.049 |
| Inferior Temporal Gyrus – temporo-occipital part | 0.838 | 0.998 | -0.013 |
| Postcentral Gyrus | 0.859 | 0.998 | 0.013 |
| Superior Parietal Lobule | 0.971 | 0.998 | -0.003 |
| Supramarginal Gyrus – anterior division | 0.356 | 0.998 | 0.070 |
| Supramarginal Gyrus – posterior division | 0.924 | 0.998 | -0.006 |
| Angular Gyrus | 0.682 | 0.998 | -0.024 |
| Lateral Occipital Cortex – superior division | 0.989 | 0.998 | -0.001 |
| Lateral Occipital Cortex – inferior division | 0.866 | 0.998 | -0.012 |
| Intracalcarine Cortex | 0.502 | 0.998 | -0.058 |
| Frontal Medial Cortex | 0.141 | 0.998 | 0.124 |
| Juxtapositional Lobule Cortex | 0.866 | 0.998 | 0.014 |
| Subcallosal Cortex | 0.303 | 0.998 | -0.084 |
| Paracingulate Gyrus | 0.756 | 0.998 | -0.025 |
| Cingulate Gyrus – anterior division | 0.328 | 0.998 | 0.082 |
| Cingulate Gyrus – posterior division | 0.731 | 0.998 | 0.028 |
| Precuneous Cortex | 0.695 | 0.998 | -0.032 |
| Cuneal Cortex | 0.219 | 0.998 | 0.091 |
| Frontal Orbital Cortex | 0.404 | 0.998 | -0.060 |
| Parahippocampal Gyrus – anterior division | 0.761 | 0.998 | 0.024 |
| Parahippocampal Gyrus – posterior division | 0.360 | 0.998 | 0.058 |
| Lingual Gyrus | 0.858 | 0.998 | 0.013 |
| Temporal Fusiform Cortex – anterior division | 0.475 | 0.998 | -0.053 |
| Temporal Fusiform Cortex – posterior division | 0.736 | 0.998 | -0.022 |
| Temporal Occipital Fusiform Cortex | 0.977 | 0.998 | -0.002 |
| Occipital Fusiform Cortex | 0.227 | 0.998 | -0.088 |
| Frontal Operculum Cortex | 0.829 | 0.998 | 0.015 |
| Central Opercular Cortex | 0.881 | 0.998 | -0.012 |
| Parietal Operculum Cortex | 0.128 | 0.998 | -0.116 |
| Planum Polare | 0.338 | 0.998 | -0.074 |
| Heschl’s Gyrus | 0.437 | 0.998 | -0.056 |
| Planum Temporale | 0.240 | 0.998 | -0.077 |
| Supracalcarine Cortex | 0.607 | 0.998 | -0.034 |
| Occipital Pole | 0.863 | 0.998 | -0.013 |

Table 47: Effect of 'communications' PE on cortical volumes

| Brain Structure | P-values | FDR P-value | β-coefficient |
| --- | --- | --- | --- |
| Frontal Pole | 0.866 | 0.986 | -0.009 |
| Insular Cortex | 0.664 | 0.942 | 0.018 |
| Superior Frontal Gyrus | 0.199 | 0.599 | -0.093 |
| Middle Frontal Gyrus | 0.287 | 0.657 | 0.086 |
| Inferior Frontal Gyrus – pars triangularis | 0.964 | 0.986 | 0.005 |
| Inferior Frontal Gyrus – pars opercularis | 0.466 | 0.713 | 0.079 |
| Precentral Gyrus | 0.942 | 0.986 | -0.005 |
| Temporal Pole | 0.986 | 0.986 | -0.001 |
| Superior Temporal Gyrus – anterior division | 0.222 | 0.599 | -0.124 |
| Superior Temporal Gyrus – posterior division | 0.164 | 0.599 | 0.122 |
| Middle Temporal Gyrus – anterior division | 0.044 | 0.599 | -0.187 |
| Middle Temporal Gyrus – posterior division | 0.347 | 0.690 | 0.083 |
| Middle Temporal Gyrus – temporo-occipital part | 0.908 | 0.986 | 0.010 |
| Inferior Temporal Gyrus – anterior division | 0.263 | 0.632 | 0.114 |
| Inferior Temporal Gyrus – posterior division | 0.341 | 0.690 | 0.088 |
| Inferior Temporal Gyrus – temporo-occipital part | 0.305 | 0.665 | -0.086 |
| Postcentral Gyrus | 0.237 | 0.599 | 0.097 |
| Superior Parietal Lobule | 0.221 | 0.599 | -0.126 |
| Supramarginal Gyrus – anterior division | 0.229 | 0.599 | -0.132 |
| Supramarginal Gyrus – posterior division | 0.475 | 0.713 | 0.073 |
| Angular Gyrus | 0.815 | 0.986 | -0.021 |
| Lateral Occipital Cortex – superior division | 0.105 | 0.599 | 0.143 |
| Lateral Occipital Cortex – inferior division | 0.019 | 0.599 | -0.228 |
| Intracalcarine Cortex | 0.851 | 0.986 | 0.010 |
| Frontal Medial Cortex | 0.168 | 0.599 | -0.092 |
| Juxtapositional Lobule Cortex | 0.076 | 0.599 | -0.116 |
| Subcallosal Cortex | 0.385 | 0.690 | -0.036 |
| Paracingulate Gyrus | 0.089 | 0.599 | 0.105 |
| Cingulate Gyrus – anterior division | 0.183 | 0.599 | -0.081 |
| Cingulate Gyrus – posterior division | 0.775 | 0.986 | 0.013 |
| Precuneous Cortex | 0.722 | 0.963 | 0.018 |
| Cuneal Cortex | 0.135 | 0.599 | 0.111 |
| Frontal Orbital Cortex | 0.403 | 0.690 | 0.053 |
| Parahippocampal Gyrus – anterior division | 0.379 | 0.690 | -0.060 |
| Parahippocampal Gyrus – posterior division | 0.445 | 0.713 | 0.048 |
| Lingual Gyrus | 0.083 | 0.599 | -0.112 |
| Temporal Fusiform Cortex – anterior division | 0.228 | 0.599 | 0.104 |
| Temporal Fusiform Cortex – posterior division | 0.093 | 0.599 | 0.101 |
| Temporal Occipital Fusiform Cortex | 0.975 | 0.986 | -0.002 |
| Occipital Fusiform Cortex | 0.216 | 0.599 | 0.117 |
| Frontal Operculum Cortex | 0.667 | 0.942 | -0.039 |
| Central Opercular Cortex | 0.402 | 0.690 | 0.065 |
| Parietal Operculum Cortex | 0.439 | 0.713 | -0.071 |
| Planum Polare | 0.845 | 0.986 | -0.015 |
| Heschl’s Gyrus | 0.885 | 0.986 | 0.012 |
| Planum Temporale | 0.912 | 0.986 | -0.009 |
| Supracalcarine Cortex | 0.192 | 0.599 | -0.080 |
| Occipital Pole | 0.705 | 0.963 | -0.030 |

Table 48: Interaction effect between hemisphere and 'communications' PE on cortical volumes

| Brain Structure | P-values | FDR P-value | β-coefficient |
| --- | --- | --- | --- |
| Frontal Pole | 0.620 | 0.866 | -0.067 |
| Insular Cortex | 0.508 | 0.866 | -0.110 |
| Superior Frontal Gyrus | 0.741 | 0.866 | -0.049 |
| Middle Frontal Gyrus | 0.137 | 0.827 | -0.236 |
| Inferior Frontal Gyrus – pars triangularis | 0.856 | 0.893 | 0.027 |
| Inferior Frontal Gyrus – pars opercularis | 0.343 | 0.866 | -0.137 |
| Precentral Gyrus | 0.054 | 0.827 | -0.303 |
| Temporal Pole | 0.603 | 0.866 | 0.080 |
| Superior Temporal Gyrus – anterior division | 0.758 | 0.866 | -0.047 |
| Superior Temporal Gyrus – posterior division | 0.572 | 0.866 | 0.076 |
| Middle Temporal Gyrus – anterior division | 0.833 | 0.889 | 0.031 |
| Middle Temporal Gyrus – posterior division | 0.614 | 0.866 | -0.079 |
| Middle Temporal Gyrus – temporo-occipital part | 0.186 | 0.827 | -0.162 |
| Inferior Temporal Gyrus – anterior division | 0.419 | 0.866 | 0.122 |
| Inferior Temporal Gyrus – posterior division | 0.355 | 0.866 | 0.145 |
| Inferior Temporal Gyrus – temporo-occipital part | 0.017 | 0.817 | -0.308 |
| Postcentral Gyrus | 0.304 | 0.866 | -0.156 |
| Superior Parietal Lobule | 0.134 | 0.827 | 0.221 |
| Supramarginal Gyrus – anterior division | 0.222 | 0.827 | -0.186 |
| Supramarginal Gyrus – posterior division | 0.522 | 0.866 | -0.086 |
| Angular Gyrus | 0.736 | 0.866 | -0.040 |
| Lateral Occipital Cortex – superior division | 0.364 | 0.866 | -0.137 |
| Lateral Occipital Cortex – inferior division | 0.776 | 0.866 | -0.041 |
| Intracalcarine Cortex | 0.666 | 0.866 | 0.074 |
| Frontal Medial Cortex | 0.533 | 0.866 | -0.105 |
| Juxtapositional Lobule Cortex | 0.380 | 0.866 | -0.151 |
| Subcallosal Cortex | 0.141 | 0.827 | -0.239 |
| Paracingulate Gyrus | 0.712 | 0.866 | 0.060 |
| Cingulate Gyrus – anterior division | 0.962 | 0.962 | -0.008 |
| Cingulate Gyrus – posterior division | 0.224 | 0.827 | -0.195 |
| Precuneous Cortex | 0.531 | 0.866 | -0.102 |
| Cuneal Cortex | 0.447 | 0.866 | 0.114 |
| Frontal Orbital Cortex | 0.224 | 0.827 | -0.176 |
| Parahippocampal Gyrus – anterior division | 0.728 | 0.866 | 0.055 |
| Parahippocampal Gyrus – posterior division | 0.128 | 0.827 | -0.192 |
| Lingual Gyrus | 0.668 | 0.866 | 0.062 |
| Temporal Fusiform Cortex – anterior division | 0.486 | 0.866 | 0.103 |
| Temporal Fusiform Cortex – posterior division | 0.638 | 0.866 | 0.061 |
| Temporal Occipital Fusiform Cortex | 0.631 | 0.866 | -0.060 |
| Occipital Fusiform Cortex | 0.223 | 0.827 | -0.178 |
| Frontal Operculum Cortex | 0.200 | 0.827 | -0.184 |
| Central Opercular Cortex | 0.511 | 0.866 | -0.102 |
| Parietal Operculum Cortex | 0.652 | 0.866 | 0.069 |
| Planum Polare | 0.897 | 0.916 | 0.020 |
| Heschl’s Gyrus | 0.201 | 0.827 | 0.185 |
| Planum Temporale | 0.488 | 0.866 | 0.091 |
| Supracalcarine Cortex | 0.816 | 0.889 | 0.031 |
| Occipital Pole | 0.535 | 0.866 | -0.095 |

Table 49: Interaction effect between sex and 'communications' PE on cortical volumes

### Conspiracies

| Lobes | P-Value | FDR P-value | β-coefficient |
| --- | --- | --- | --- |
| Temporal Lobe | 0.753 | 0.850 | -0.002 |
| Occipital Lobe | 0.795 | 0.850 | -0.002 |
| Parietal Lobe | 0.850 | 0.850 | -0.001 |
| Frontal Lobe | 0.486 | 0.850 | -0.005 |

Table 50: Effect of 'conspiracy' PE on volume per lobe

| Brain Structure | P-values | FDR P-value | β-coefficient |
| --- | --- | --- | --- |
| Frontal Pole | 0.973 | 0.973 | -0.002 |
| Insular Cortex | 0.308 | 0.771 | -0.083 |
| Superior Frontal Gyrus | 0.576 | 0.790 | -0.041 |
| Middle Frontal Gyrus | 0.900 | 0.958 | 0.010 |
| Inferior Frontal Gyrus – pars triangularis | 0.031 | 0.756 | -0.154 |
| Inferior Frontal Gyrus – pars opercularis | 0.027 | 0.756 | -0.156 |
| Precentral Gyrus | 0.619 | 0.803 | -0.038 |
| Temporal Pole | 0.384 | 0.790 | -0.065 |
| Superior Temporal Gyrus – anterior division | 0.213 | 0.771 | -0.092 |
| Superior Temporal Gyrus – posterior division | 0.432 | 0.790 | -0.051 |
| Middle Temporal Gyrus – anterior division | 0.938 | 0.958 | 0.006 |
| Middle Temporal Gyrus – posterior division | 0.818 | 0.958 | -0.017 |
| Middle Temporal Gyrus – temporo-occipital part | 0.535 | 0.790 | -0.037 |
| Inferior Temporal Gyrus – anterior division | 0.569 | 0.790 | -0.042 |
| Inferior Temporal Gyrus – posterior division | 0.204 | 0.771 | 0.097 |
| Inferior Temporal Gyrus – temporo-occipital part | 0.837 | 0.958 | -0.013 |
| Postcentral Gyrus | 0.504 | 0.790 | -0.049 |
| Superior Parietal Lobule | 0.698 | 0.859 | 0.028 |
| Supramarginal Gyrus – anterior division | 0.856 | 0.958 | 0.013 |
| Supramarginal Gyrus – posterior division | 0.400 | 0.790 | -0.055 |
| Angular Gyrus | 0.490 | 0.790 | 0.040 |
| Lateral Occipital Cortex – superior division | 0.309 | 0.771 | 0.075 |
| Lateral Occipital Cortex – inferior division | 0.439 | 0.790 | -0.055 |
| Intracalcarine Cortex | 0.509 | 0.790 | -0.055 |
| Frontal Medial Cortex | 0.928 | 0.958 | 0.007 |
| Juxtapositional Lobule Cortex | 0.875 | 0.958 | 0.013 |
| Subcallosal Cortex | 0.216 | 0.771 | -0.098 |
| Paracingulate Gyrus | 0.146 | 0.771 | -0.115 |
| Cingulate Gyrus – anterior division | 0.169 | 0.771 | 0.113 |
| Cingulate Gyrus – posterior division | 0.571 | 0.790 | 0.044 |
| Precuneous Cortex | 0.623 | 0.803 | 0.039 |
| Cuneal Cortex | 0.915 | 0.958 | -0.008 |
| Frontal Orbital Cortex | 0.100 | 0.771 | -0.116 |
| Parahippocampal Gyrus – anterior division | 0.450 | 0.790 | 0.058 |
| Parahippocampal Gyrus – posterior division | 0.364 | 0.790 | 0.056 |
| Lingual Gyrus | 0.515 | 0.790 | 0.046 |
| Temporal Fusiform Cortex – anterior division | 0.325 | 0.771 | -0.071 |
| Temporal Fusiform Cortex – posterior division | 0.257 | 0.771 | 0.072 |
| Temporal Occipital Fusiform Cortex | 0.125 | 0.771 | 0.094 |
| Occipital Fusiform Cortex | 0.319 | 0.771 | -0.071 |
| Frontal Operculum Cortex | 0.289 | 0.771 | -0.074 |
| Central Opercular Cortex | 0.337 | 0.771 | -0.072 |
| Parietal Operculum Cortex | 0.636 | 0.803 | -0.035 |
| Planum Polare | 0.287 | 0.771 | -0.080 |
| Heschl’s Gyrus | 0.121 | 0.771 | -0.109 |
| Planum Temporale | 0.239 | 0.771 | -0.075 |
| Supracalcarine Cortex | 0.242 | 0.771 | -0.075 |
| Occipital Pole | 0.211 | 0.771 | -0.093 |

Table 51: Effect of 'conspiracy' on cortical volumes

| Brain Structure | P-values | FDR P-value | β-coefficient |
| --- | --- | --- | --- |
| Frontal Pole | 0.794 | 0.996 | -0.013 |
| Insular Cortex | 0.259 | 0.996 | -0.046 |
| Superior Frontal Gyrus | 0.997 | 0.997 | 0.000 |
| Middle Frontal Gyrus | 0.441 | 0.996 | -0.061 |
| Inferior Frontal Gyrus – pars triangularis | 0.762 | 0.996 | -0.032 |
| Inferior Frontal Gyrus – pars opercularis | 0.785 | 0.996 | 0.029 |
| Precentral Gyrus | 0.318 | 0.996 | -0.067 |
| Temporal Pole | 0.303 | 0.996 | -0.074 |
| Superior Temporal Gyrus – anterior division | 0.442 | 0.996 | 0.076 |
| Superior Temporal Gyrus – posterior division | 0.220 | 0.996 | -0.104 |
| Middle Temporal Gyrus – anterior division | 0.898 | 0.996 | 0.012 |
| Middle Temporal Gyrus – posterior division | 0.286 | 0.996 | -0.092 |
| Middle Temporal Gyrus – temporo-occipital part | 0.026 | 0.996 | 0.188 |
| Inferior Temporal Gyrus – anterior division | 0.132 | 0.996 | 0.149 |
| Inferior Temporal Gyrus – posterior division | 0.342 | 0.996 | 0.085 |
| Inferior Temporal Gyrus – temporo-occipital part | 0.510 | 0.996 | 0.054 |
| Postcentral Gyrus | 0.599 | 0.996 | 0.042 |
| Superior Parietal Lobule | 0.918 | 0.996 | 0.010 |
| Supramarginal Gyrus – anterior division | 0.768 | 0.996 | 0.032 |
| Supramarginal Gyrus – posterior division | 0.655 | 0.996 | -0.045 |
| Angular Gyrus | 0.140 | 0.996 | 0.127 |
| Lateral Occipital Cortex – superior division | 0.967 | 0.996 | -0.004 |
| Lateral Occipital Cortex – inferior division | 0.919 | 0.996 | -0.010 |
| Intracalcarine Cortex | 0.514 | 0.996 | 0.034 |
| Frontal Medial Cortex | 0.747 | 0.996 | -0.021 |
| Juxtapositional Lobule Cortex | 0.754 | 0.996 | 0.020 |
| Subcallosal Cortex | 0.469 | 0.996 | -0.030 |
| Paracingulate Gyrus | 0.337 | 0.996 | 0.058 |
| Cingulate Gyrus – anterior division | 0.610 | 0.996 | -0.030 |
| Cingulate Gyrus – posterior division | 0.417 | 0.996 | -0.036 |
| Precuneous Cortex | 0.535 | 0.996 | 0.031 |
| Cuneal Cortex | 0.222 | 0.996 | 0.088 |
| Frontal Orbital Cortex | 0.049 | 0.996 | 0.121 |
| Parahippocampal Gyrus – anterior division | 0.975 | 0.996 | 0.002 |
| Parahippocampal Gyrus – posterior division | 0.847 | 0.996 | 0.012 |
| Lingual Gyrus | 0.756 | 0.996 | -0.020 |
| Temporal Fusiform Cortex – anterior division | 0.075 | 0.996 | 0.150 |
| Temporal Fusiform Cortex – posterior division | 0.582 | 0.996 | 0.032 |
| Temporal Occipital Fusiform Cortex | 0.209 | 0.996 | 0.095 |
| Occipital Fusiform Cortex | 0.584 | 0.996 | -0.051 |
| Frontal Operculum Cortex | 0.849 | 0.996 | 0.017 |
| Central Opercular Cortex | 0.846 | 0.996 | -0.015 |
| Parietal Operculum Cortex | 0.445 | 0.996 | 0.068 |
| Planum Polare | 0.955 | 0.996 | -0.004 |
| Heschl’s Gyrus | 0.370 | 0.996 | -0.069 |
| Planum Temporale | 0.884 | 0.996 | -0.011 |
| Supracalcarine Cortex | 0.704 | 0.996 | 0.023 |
| Occipital Pole | 0.857 | 0.996 | 0.014 |

Table 52: Interaction of hemisphere and 'conspiracy' PE on cortical volumes

| Brain Structure | P-values | FDR P-value | β-coefficient |
| --- | --- | --- | --- |
| Frontal Pole | 0.666 | 0.905 | -0.057 |
| Insular Cortex | 0.183 | 0.601 | 0.218 |
| Superior Frontal Gyrus | 0.023 | 0.534 | 0.335 |
| Middle Frontal Gyrus | 0.446 | 0.785 | -0.119 |
| Inferior Frontal Gyrus – pars triangularis | 0.565 | 0.861 | 0.083 |
| Inferior Frontal Gyrus – pars opercularis | 0.953 | 0.953 | 0.008 |
| Precentral Gyrus | 0.698 | 0.905 | -0.060 |
| Temporal Pole | 0.213 | 0.601 | 0.188 |
| Superior Temporal Gyrus – anterior division | 0.690 | 0.905 | 0.059 |
| Superior Temporal Gyrus – posterior division | 0.086 | 0.558 | 0.226 |
| Middle Temporal Gyrus – anterior division | 0.574 | 0.861 | 0.081 |
| Middle Temporal Gyrus – posterior division | 0.148 | 0.586 | 0.221 |
| Middle Temporal Gyrus – temporo-occipital part | 0.799 | 0.913 | -0.031 |
| Inferior Temporal Gyrus – anterior division | 0.898 | 0.953 | 0.019 |
| Inferior Temporal Gyrus – posterior division | 0.293 | 0.704 | 0.162 |
| Inferior Temporal Gyrus – temporo-occipital part | 0.368 | 0.785 | 0.114 |
| Postcentral Gyrus | 0.950 | 0.953 | -0.009 |
| Superior Parietal Lobule | 0.043 | 0.534 | 0.294 |
| Supramarginal Gyrus – anterior division | 0.596 | 0.867 | -0.079 |
| Supramarginal Gyrus – posterior division | 0.718 | 0.905 | 0.048 |
| Angular Gyrus | 0.159 | 0.586 | 0.165 |
| Lateral Occipital Cortex – superior division | 0.391 | 0.785 | -0.127 |
| Lateral Occipital Cortex – inferior division | 0.949 | 0.953 | -0.009 |
| Intracalcarine Cortex | 0.050 | 0.534 | 0.332 |
| Frontal Medial Cortex | 0.478 | 0.791 | -0.118 |
| Juxtapositional Lobule Cortex | 0.056 | 0.534 | 0.323 |
| Subcallosal Cortex | 0.341 | 0.780 | 0.152 |
| Paracingulate Gyrus | 0.773 | 0.905 | 0.046 |
| Cingulate Gyrus – anterior division | 0.230 | 0.613 | 0.199 |
| Cingulate Gyrus – posterior division | 0.458 | 0.785 | -0.117 |
| Precuneous Cortex | 0.451 | 0.785 | 0.120 |
| Cuneal Cortex | 0.119 | 0.572 | 0.229 |
| Frontal Orbital Cortex | 0.427 | 0.785 | 0.113 |
| Parahippocampal Gyrus – anterior division | 0.100 | 0.558 | 0.257 |
| Parahippocampal Gyrus – posterior division | 0.822 | 0.918 | 0.028 |
| Lingual Gyrus | 0.249 | 0.630 | 0.165 |
| Temporal Fusiform Cortex – anterior division | 0.105 | 0.558 | 0.235 |
| Temporal Fusiform Cortex – posterior division | 0.094 | 0.558 | 0.214 |
| Temporal Occipital Fusiform Cortex | 0.560 | 0.861 | 0.072 |
| Occipital Fusiform Cortex | 0.766 | 0.905 | 0.043 |
| Frontal Operculum Cortex | 0.918 | 0.953 | 0.015 |
| Central Opercular Cortex | 0.153 | 0.586 | 0.217 |
| Parietal Operculum Cortex | 0.412 | 0.785 | 0.123 |
| Planum Polare | 0.210 | 0.601 | 0.190 |
| Heschl’s Gyrus | 0.016 | 0.534 | 0.341 |
| Planum Temporale | 0.191 | 0.601 | 0.169 |
| Supracalcarine Cortex | 0.751 | 0.905 | 0.041 |
| Occipital Pole | 0.771 | 0.905 | -0.044 |

Table 53: Interaction effect of sex and 'conspiracy' PE on cortical volumes

## Grey matter subcortical volumes

### Any PE

| Brain Structure | P-value | FDR P-Value | β-coefficient [95% CI] |
| --- | --- | --- | --- |
| Thalamus | 0.174 | 0.274 | -0.043 [-0.104;0.019] |
| Caudate | 0.032 | 0.110 | -0.075 [-0.143;-0.007] |
| Putamen | 0.145 | 0.274 | -0.046 [-0.108;0.016] |
| Pallidum | 0.685 | 0.685 | -0.014 [-0.080;0.053] |
| Hippocampus | 0.356 | 0.416 | 0.030 [-0.034;0.094] |
| Amygdala | 0.195 | 0.274 | 0.040 [-0.020;0.100] |
| Accumbens | 0.010 | 0.072 | -0.072 [-0.127;-0.017] |

Table 8: Effects of any PE (N=768) on subcortical volumes

| Brain Structure | P-value | FDR P-Value | β-coefficient |
| --- | --- | --- | --- |
| Thalamus | 0.689 | 0.952 | -0.005 |
| Caudate | 0.302 | 0.688 | -0.018 |
| Putamen | 0.377 | 0.688 | -0.019 |
| Pallidum | 0.997 | 0.997 | 0.000 |
| Hippocampus | 0.393 | 0.688 | -0.029 |
| Amygdala | 0.816 | 0.952 | 0.010 |
| Accumbens | 0.090 | 0.631 | 0.053 |

Table 9: Hemisphere-PE interaction effect for any PE on volume of subcortical structures

| Brain Structure | P-value | FDR P-Value | β-coefficient |
| --- | --- | --- | --- |
| Thalamus | 0.698 | 0.981 | -0.025 |
| Caudate | 0.027 | 0.188 | -0.157 |
| Putamen | 0.841 | 0.981 | 0.013 |
| Pallidum | 0.250 | 0.584 | -0.079 |
| Hippocampus | 0.776 | 0.981 | -0.019 |
| Amygdala | 0.082 | 0.287 | 0.109 |
| Accumbens | 0.993 | 0.993 | 0.000 |

Table 10: Sex-PE interaction effect for any PE on volume of subcortical structures

| Brain Structure | P-value | FDR P-Value | β-coefficient |
| --- | --- | --- | --- |
| Thalamus | 0.175 | 0.649 | 0.087 |
| Caudate | 0.513 | 0.915 | 0.047 |
| Putamen | 0.915 | 0.915 | -0.007 |
| Pallidum | 0.719 | 0.915 | -0.025 |
| Hippocampus | 0.831 | 0.915 | 0.014 |
| Amygdala | 0.526 | 0.915 | -0.040 |
| Accumbens | 0.185 | 0.649 | 0.077 |

Table 11: Depression-PE interaction effect for any PE on volume of subcortical structures

### Visions

| Brain Structure | P-value | FDR P-Value | β-coefficient [95% CI] |
| --- | --- | --- | --- |
| Thalamus | 0.714 | 0.833 | -0.014 [-0.092;0.063] |
| Caudate | 0.121 | 0.425 | -0.068 [-0.153;0.018] |
| Putamen | 0.270 | 0.472 | -0.044 [-0.121;0.034] |
| Pallidum | 0.897 | 0.897 | 0.005 [-0.078;0.089] |
| Hippocampus | 0.445 | 0.623 | 0.031 [-0.049;0.111] |
| Amygdala | 0.206 | 0.472 | 0.049 [-0.027;0.125] |
| Accumbens | 0.030 | 0.207 | -0.077 [-0.146;-0.008] |

Table 12: Effect of ‘Visions’ PE on subcortical volumes

| Brain Structure | P-value | FDR P-Value | β-coefficient |
| --- | --- | --- | --- |
| Thalamus | 0.370 | 0.518 | -0.015 |
| Caudate | 0.071 | 0.338 | -0.039 |
| Putamen | 0.964 | 0.964 | 0.001 |
| Pallidum | 0.766 | 0.893 | 0.010 |
| Hippocampus | 0.118 | 0.338 | -0.067 |
| Amygdala | 0.145 | 0.338 | 0.078 |
| Accumbens | 0.359 | 0.518 | 0.036 |

Table 13: Interaction effect between ‘Visions’ PE and hemisphere on subcortical volumes

| Brain Structure | P-value | FDR P-Value | β-coefficient |
| --- | --- | --- | --- |
| Thalamus | 0.700 | 0.700 | -0.032 |
| Caudate | 0.056 | 0.394 | -0.176 |
| Putamen | 0.330 | 0.591 | -0.081 |
| Pallidum | 0.190 | 0.591 | -0.117 |
| Hippocampus | 0.594 | 0.693 | -0.046 |
| Amygdala | 0.337 | 0.591 | 0.078 |
| Accumbens | 0.468 | 0.655 | -0.054 |

Table 14: Interaction effect between sex and ‘Visions’ PE on subcortical volumes

| Brain Structure | P-value | FDR P-Value | β-coefficient |
| --- | --- | --- | --- |
| Thalamus | 0.665 | 0.723 | 0.035 |
| Caudate | 0.702 | 0.723 | -0.034 |
| Putamen | 0.632 | 0.723 | -0.039 |
| Pallidum | 0.421 | 0.723 | -0.070 |
| Hippocampus | 0.723 | 0.723 | -0.030 |
| Amygdala | 0.220 | 0.723 | -0.097 |
| Accumbens | 0.271 | 0.723 | 0.080 |

Table 15: Interaction effect between depression and ‘Visions’ PE on subcortical volumes

### Voices

| Brain Structure | P-value | FDR P-value | Β-coefficient |
| --- | --- | --- | --- |
| Thalamus | 0.546 | 0.774 | -0.013 |
| Caudate | 0.663 | 0.774 | -0.012 |
| Putamen | 0.577 | 0.774 | -0.019 |
| Pallidum | 0.319 | 0.774 | 0.043 |
| Hippocampus | 0.800 | 0.800 | -0.014 |
| Amygdala | 0.537 | 0.774 | 0.042 |
| Accumbens | 0.110 | 0.770 | 0.079 |

Table 16: Interaction effects between hemisphere and ‘Voices’ PE on subcortical volumes

| Brain Structure | P-value | FDR P-value | Β-coefficient |
| --- | --- | --- | --- |
| Thalamus | 0.123 | 0.513 | 0.154 |
| Caudate | 0.237 | 0.513 | 0.131 |
| Putamen | 0.747 | 0.872 | 0.032 |
| Pallidum | 0.293 | 0.513 | -0.114 |
| Hippocampus | 0.892 | 0.892 | -0.014 |
| Amygdala | 0.257 | 0.513 | -0.112 |
| Accumbens | 0.736 | 0.872 | 0.030 |

Table 17: Interaction effect between depression and ‘Voices’ PE on subcortical volumes

| Brain Structure | P-value | FDR P-value | Β-coefficient |
| --- | --- | --- | --- |
| Thalamus | 0.670 | 0.781 | 0.043 |
| Caudate | 0.575 | 0.781 | -0.063 |
| Putamen | 0.266 | 0.621 | 0.112 |
| Pallidum | 0.885 | 0.885 | 0.016 |
| Hippocampus | 0.027 | 0.134 | 0.232 |
| Amygdala | 0.038 | 0.134 | 0.205 |
| Accumbens | 0.641 | 0.781 | -0.042 |

Table 18: Interaction effect of sex and ‘Voices’ PE on subcortical volumes

| Brain Structure | P-value | FDR P-value | Β-coefficient |
| --- | --- | --- | --- |
| Thalamus | 0.036 | 0.127 | -0.165 |
| Caudate | 0.333 | 0.433 | -0.085 |
| Putamen | 0.090 | 0.209 | -0.134 |
| Pallidum | 0.183 | 0.320 | -0.113 |
| Hippocampus | 0.654 | 0.654 | -0.037 |
| Amygdala | 0.371 | 0.433 | -0.069 |
| Accumbens | 0.026 | 0.127 | -0.158 |

Table 19: Effect of ‘Voices’ PE on subcortical volumes in distressed cases only (N=96)

| Brain Structure | P-value | FDR P-Value | β-coefficient [95% CI] |
| --- | --- | --- | --- |
| Thalamus | 0.048 | 0.083 | -0.099 [-0.197;-0.001] |
| Caudate * | 0.018 | 0.041 | -0.131 [-0.240;-0.023] |
| Putamen * | 0.006 | 0.031 | -0.138 [-0.236;-0.040] |
| Pallidum | 0.083 | 0.117 | -0.093 [-0.199;0.012] |
| Hippocampus | 0.265 | 0.265 | -0.058 [-0.159;0.044] |
| Amygdala | 0.212 | 0.248 | -0.061 [-0.157;0.035] |
| Accumbens * | 0.009 | 0.031 | -0.117 [-0.205;-0.029] |

Table 20: Effect of ‘Voices’ PE on subcortical volumes with cannabis use added as a covariate to the model (N=791 as heavy users, of which 34 reported an ‘Voices’ PE)

| Brain Structure | P-value | FDR P-Value | β-coefficient [95% CI] |  |
| --- | --- | --- | --- | --- |
| Thalamus | 0.275 | 0.134 | -0.089 [-0.186;0.009] |  |
| Caudate | 0.014 | 0.050 | -0.128 [-0.236;-0.019] |  |
| Putamen * | 0.553 | 0.048 | -0.132 [-0.230;-0.034] |  |
| Pallidum | 0.277 | 0.157 | -0.086 [-0.191;0.020] |  |
| Hippocampus | 0.217 | 0.275 | -0.060 [-0.162;0.041] |  |
| Amygdala | 0.574 | 0.275 | -0.054 [-0.150;0.043] |  |
| Accumbens * | 0.003 | 0.048 | -0.111 [-0.199;-0.023] |  |

Table 21: Effect of Voices on subcortical structures with childhood trauma (N=5376 with trauma, of which 160 heard voices)

| Brain Structure | P-value | FDR P-Value | β-coefficient [95% CI] |
| --- | --- | --- | --- |
| Thalamus | 0.052 | 0.091 | -0.097 [-0.195;0.001] |
| Caudate * | 0.020 | 0.047 | -0.128 [-0.237;-0.020] |
| Putamen * | 0.007 | 0.036 | -0.134 [-0.232;-0.036] |
| Pallidum | 0.091 | 0.128 | -0.091 [-0.196;0.015] |
| Hippocampus | 0.267 | 0.267 | -0.057 [-0.159;0.044] |
| Amygdala | 0.245 | 0.267 | -0.057 [-0.153;0.039] |
| Accumbens * | 0.010 | 0.036 | -0.115 [-0.203;-0.027] |

Table 22: Effect of ‘Voices’ PE on subcortical volumes with antipsychotic medication use as covariate

| Brain Structure | P-value | FDR P-Value | β-coefficient [95% CI] |
| --- | --- | --- | --- |
| Thalamus | 0.119 | 0.167 | -0.081 [-0.183;0.021] |
| Caudate | 0.035 | 0.082 | -0.121 [-0.234;-0.008] |
| Putamen * | 0.004 | 0.029 | -0.150 [-0.252;-0.047] |
| Pallidum | 0.090 | 0.158 | -0.095 [-0.205;0.015] |
| Hippocampus | 0.372 | 0.372 | -0.048 [-0.154;0.057] |
| Amygdala | 0.220 | 0.257 | -0.063 [-0.163;0.037] |
| Accumbens | 0.015 | 0.051 | -0.114 [-0.206;-0.023] |

Table 23: Effect of ‘Voices’ PE on subcortical volumes excluding subjects with self-reported diagnosis of psychosis

### Communications

| Brain Structure | P-value | FDR P-Value | β-coefficient |
| --- | --- | --- | --- |
| Thalamus | 0.060 | 0.421 | -0.153 [-0.312;0.006] |
| Caudate | 0.152 | 0.533 | -0.129 [-0.305;0.048] |
| Putamen | 0.679 | 0.880 | -0.034 [-0.194;0.126] |
| Pallidum | 0.312 | 0.727 | -0.089 [-0.261;0.083] |
| Hippocampus | 0.909 | 0.909 | 0.010 [-0.156;0.175] |
| Amygdala | 0.683 | 0.880 | 0.033 [-0.124;0.189] |
| Accumbens | 0.754 | 0.880 | -0.023 [-0.166;0.120] |

Table 24: Effect of 'communications' PE on subcortical volumes

| Brain Structure | P-value | FDR P-Value | β-coefficient |
| --- | --- | --- | --- |
| Thalamus | 0.941 | 0.941 | -0.003 |
| Caudate | 0.661 | 0.925 | 0.019 |
| Putamen | 0.375 | 0.925 | 0.050 |
| Pallidum | 0.888 | 0.941 | -0.010 |
| Hippocampus | 0.589 | 0.925 | -0.047 |
| Amygdala | 0.401 | 0.925 | -0.093 |
| Accumbens | 0.516 | 0.925 | 0.052 |

Table 25: Interaction effects between hemisphere and 'communication' PE on subcortical volumes

| Brain Structure | P-value | FDR P-Value | β-coefficient |
| --- | --- | --- | --- |
| Thalamus | 0.924 | 0.997 | -0.015 |
| Caudate | 0.537 | 0.997 | -0.111 |
| Putamen | 0.827 | 0.997 | -0.036 |
| Pallidum | 0.908 | 0.997 | 0.020 |
| Hippocampus | 0.366 | 0.997 | -0.153 |
| Amygdala | 0.997 | 0.997 | 0.001 |
| Accumbens | 0.339 | 0.997 | 0.140 |

Table 26: Interaction effects between sex and 'communications' PE on subcortical volumes

| Brain Structure | P-value | FDR P-Value | β-coefficient |
| --- | --- | --- | --- |
| Thalamus | 0.557 | 0.953 | -0.096 |
| Caudate | 0.817 | 0.953 | -0.042 |
| Putamen | 0.762 | 0.953 | -0.050 |
| Pallidum | 0.806 | 0.953 | -0.043 |
| Hippocampus | 0.768 | 0.953 | -0.050 |
| Amygdala | 0.286 | 0.953 | -0.171 |
| Accumbens | 0.961 | 0.961 | -0.007 |

Table 27: Interaction effect between depression and 'communication' PE on subcortical volumes

### Conspiracies

| Brain Structure | P-value | FDR P-Value | β-coefficient |
| --- | --- | --- | --- |
| Thalamus | 0.016 | 0.067 | -0.189 [-0.343;-0.035] |
| Caudate | 0.239 | 0.557 | -0.103 [-0.273;0.068] |
| Putamen | 0.570 | 0.798 | -0.045 [-0.199;0.110] |
| Pallidum | 0.973 | 0.973 | 0.003 [-0.163;0.169] |
| Hippocampus | 0.959 | 0.973 | 0.004 [-0.155;0.164] |
| Amygdala | 0.019 | 0.067 | 0.181 [0.029;0.332] |
| Accumbens | 0.346 | 0.605 | -0.067 [-0.205;0.072] |

Table 28: Effect of 'conspiracy' PE on subcortical volumes

| Brain Structure | P-value | FDR P-Value | β-coefficient |
| --- | --- | --- | --- |
| Thalamus | 0.214 | 0.420 | 0.042 |
| Caudate | 0.974 | 0.974 | -0.001 |
| Putamen | 0.240 | 0.420 | -0.063 |
| Pallidum | 0.643 | 0.750 | -0.032 |
| Hippocampus | 0.055 | 0.194 | 0.163 |
| Amygdala | 0.041 | 0.194 | -0.219 |
| Accumbens | 0.493 | 0.690 | 0.053 |

Table 29: Interaction effects between hemisphere and 'conspiracy' on subcortical volumes

| Brain Structure | P-value | FDR P-Value | β-coefficient |
| --- | --- | --- | --- |
| Thalamus | 0.774 | 0.774 | -0.045 |
| Caudate | 0.483 | 0.774 | -0.123 |
| Putamen | 0.309 | 0.774 | 0.161 |
| Pallidum | 0.572 | 0.774 | -0.096 |
| Hippocampus | 0.690 | 0.774 | 0.065 |
| Amygdala | 0.273 | 0.774 | 0.170 |
| Accumbens | 0.494 | 0.774 | 0.097 |

Table 30: Interaction effects between sex and 'conspiracy' PE on subcortical volumes

| Brain Structure | P-value | FDR P-Value | β-coefficient |
| --- | --- | --- | --- |
| Thalamus | 0.723 | 0.942 | 0.057 |
| Caudate | 0.779 | 0.942 | 0.050 |
| Putamen | 0.942 | 0.942 | -0.012 |
| Pallidum | 0.842 | 0.942 | -0.034 |
| Hippocampus | 0.933 | 0.942 | 0.014 |
| Amygdala | 0.831 | 0.942 | -0.033 |
| Accumbens | 0.647 | 0.942 | -0.066 |

Table 31: Interaction effects between depression and 'conspiracy' PE on subcortical volumes

| Brain structure | P-value | FDR P-Value | β-coefficient |
| --- | --- | --- | --- |
| Volume L Thalamus | 0.010 | 0.078 | -0.018 |
| Volume R Thalamus | 0.033 | 0.167 | -0.015 |
| Volume L Caudate | 0.218 | 0.619 | -0.010 |
| Volume R Caudate | 0.289 | 0.619 | -0.008 |
| Volume L Putamen | 0.823 | 0.823 | -0.002 |
| Volume R Putamen | 0.391 | 0.622 | -0.006 |
| Volume L Pallidum | 0.758 | 0.823 | 0.002 |
| Volume R Pallidum | 0.803 | 0.823 | -0.002 |
| Volume L Hippocampus | 0.414 | 0.622 | -0.006 |
| Volume R Hippocampus | 0.367 | 0.622 | 0.007 |
| Volume L Amygdala * | 0.001 | 0.012 | 0.027 |
| Volume R Amygdala | 0.515 | 0.702 | 0.005 |
| Volume L Accumbens | 0.245 | 0.619 | -0.009 |
| Volume R Accumbens | 0.644 | 0.805 | -0.003 |

Table 32: Effect of 'conspiracies' PE on subcortical volumes in LM model with separate hemisphere

| Brain structure | P-value | FDR P-Value | β-coefficient |
| --- | --- | --- | --- |
| Volume L Thalamus | 0.009 | 0.067 | -0.018 |
| Volume R Thalamus | 0.031 | 0.153 | -0.015 |
| Volume L Caudate | 0.196 | 0.562 | -0.010 |
| Volume R Caudate | 0.262 | 0.562 | -0.009 |
| Volume L Putamen | 0.721 | 0.809 | -0.003 |
| Volume R Putamen | 0.355 | 0.615 | -0.007 |
| Volume L Pallidum | 0.809 | 0.809 | 0.002 |
| Volume R Pallidum | 0.770 | 0.809 | -0.002 |
| Volume L Hippocampus | 0.414 | 0.621 | -0.007 |
| Volume R Hippocampus | 0.369 | 0.615 | 0.007 |
| Volume L Amygdala | **0.001** | **0.016** | **0.026** |
| Volume R Amygdala | 0.577 | 0.743 | 0.005 |
| Volume L Accumbens | 0.234 | 0.562 | -0.009 |
| Volume R Accumbens | 0.594 | 0.743 | -0.004 |

Table 33: Effect of 'conspiracy' PE on subcortical volumes with cannabis use as covariate

| Brain structure | P-value | FDR P-Value | β-coefficient |
| --- | --- | --- | --- |
| Volume L Thalamus | 0.010 | 0.101 | -0.018 |
| Volume R Thalamus | 0.033 | 0.211 | -0.015 |
| Volume L Caudate | 0.218 | 0.607 | -0.010 |
| Volume R Caudate | 0.289 | 0.607 | -0.008 |
| Volume L Putamen | 0.823 | 0.823 | -0.002 |
| Volume R Putamen | 0.391 | 0.607 | -0.006 |
| Volume L Pallidum | 0.758 | 0.823 | 0.002 |
| Volume R Pallidum | 0.803 | 0.823 | -0.002 |
| Volume L Hippocampus | 0.414 | 0.607 | -0.006 |
| Volume R Hippocampus | 0.367 | 0.607 | 0.007 |
| Volume L Amygdala * | 0.001 | 0.015 | 0.027 |
| Volume R Amygdala | 0.515 | 0.629 | 0.005 |
| Volume L Accumbens | 0.245 | 0.607 | -0.009 |
| Volume R Accumbens | 0.644 | 0.747 | -0.003 |

Table 34: Effect of 'conspiracy' PE on subcortical volumes with antipsychotic medication as covariate

### Cannabis as mediator

We examined potential mediating effects of cannabis use and adverse life events on the structures that had significant associations with PEs. Adding cannabis use or adverse life events as covariates to the models did not change the pattern of results. The volume of the putamen in the ‘voices’ group showed a near significant decrease of volume when cannabis was added to the model as the explanatory variable, with a small negative effect size (β= -0.0783, p_corrected_=0.061) and cannabis showed a strong significant association with the presence of unreal voices (χ² statistic=50.81, p-value= 1.02e-12). Based on these results, we tested for a possible mediating effect of cannabis on the association with the ‘voices’ PE and the volume of the putamen. After finding a best fit for the model with the volume of the putamen as the dependent variable, with sex and age as covariates (and excluding positions in the scanner), there was only a significant for the direct effect of the ‘voices’ PE on the volume of the putamen (p=0.007 for the direct effect, p=0.427 for the indirect effect). We additionally looked at a mediation analysis where the ‘voices’ PE was the mediator for the effect of cannabis on the volume of the putamen. Again only the direct effect of cannabis on the putamen was highly significant (p=2.344e^-6^), whereas the indirect effect was not significant (p=0.422) (see supplementary material ST20-ST21).

|  | SE | Z-value | P-value | 95% CI (95%) |
| --- | --- | --- | --- | --- |
| Direct (Putamen ~ PE) * | 117.612 | -2.713 | 0.007 | [-549.58;-88.55] |
| Indirect (Putamen ~ PE + PE ~ Cannabis) | 9.750 | 0.795 | 0.427 | [-11.36;26.86] |
| Total (direct + indirect) | 124.166 | -0.895 | 0.371 | [-354.5;132.25] |

Table 35: Mediation model with Cannabis as mediator. Covariates included are: age and sex (CFI =0 .976, TLI=-0.919, RMSEA=0.052)

|  | SE | Z-value | P-value | 95% CI (95%) |
| --- | --- | --- | --- | --- |
| Direct (Putamen ~ Cannabis) * | 44.189 | 4.721 | 2.344e^-6^ | [122.02;295.23] |
| Indirect (Putamen ~ PE + PE ~ Cannabis) | 2.986 | -0.802 | 0.422 | [-8.25;3.46] |
| Total (direct + indirect) | 126.28 | -1.259 | 0.208 | [-406.48;88.52] |

Table 36: Mediation model with PE as mediator. Covariates included are: age and sex (CFI =0 .908, TLI=-0.692, RMSEA=0.102)

## Fractional anisotropy

### Any PE

| Subset | P-value | FDR P-value | Β-coefficient |
| --- | --- | --- | --- |
| General FA | 0.363 | 0.686 | 0.007 |
| Association fibres | 0.148 | 0.594 | 0.012 |
| Thalamic fibres | 0.603 | 0.686 | 0.004 |
| Sensory fibres | 0.686 | 0.686 | -0.003 |

Table 54: Effect of 'Any PE' on subsets white matter tracts

| Brain Structure | P-value | FDR P-Value | β-coefficient [95% CI] |
| --- | --- | --- | --- |
| Acoustic Radiation | 0.998 | 0.998 | 0.000 [-0.066;0.066] |
| Anterior Thalamic Radiation | 0.963 | 0.998 | -0.002 [-0.072;0.069] |
| Cingulate gyrus – part of cingulum | 0.270 | 0.680 | -0.033 [-0.093;0.026] |
| Parahippocampal part of cingulum | 0.823 | 0.998 | -0.008 [-0.074;0.059] |
| Corticospinal Tract | 0.363 | 0.680 | 0.033 [-0.038;0.104] |
| Inferior fronto-occipital fasciculus | 0.345 | 0.680 | -0.034 [-0.104;0.036] |
| Inferior longitudinal fasciculus | 0.084 | 0.575 | -0.061 [-0.131;0.008] |
| Medial Lemniscus | 0.766 | 0.998 | -0.010 [-0.075;0.056] |
| Posterior Thalamic Radiation | 0.115 | 0.575 | -0.056 [-0.126;0.014] |
| Superior Longitudinal fasciculus | 0.297 | 0.680 | -0.035 [-0.102;0.031] |
| Superior Thalamic Radiation | 0.216 | 0.680 | 0.046 [-0.027;0.120] |
| Uncinate Fasciculus | 0.941 | 0.998 | -0.003 [-0.070;0.065] |
| Forceps Major | 0.008 | 0.118 | -0.022 [-0.005;-0.001] |
| Forceps Minor | 0.719 | 0.998 | -0.003 [-0.002;0.001] |
| Middle Cerebellar Peduncle | 0.641 | 0.998 | 0.004 [-0.002;0.003] |

Table 55: Effect of any PE on FA values of white matter tracts

| % of variance explained | Fractional Anisotropy | Mean Diffusivity |
| --- | --- | --- |
| Global white matter integrity | 41.7% | 43.2% |
| Association tracts | 46.4% | 49.2% |
| Thalamic tracts | 65.0% | 75.1% |
| Projection tracts | 46.3% | 39.7% |

Table 56: Variance explained in different subsets of white matter tracts for both FA and MD values

| PE modality group | Global white matter integrity | | | | Association Tracts | | | | Thalamic Tracts | | | | Projection tracts | | |
| --- | --- | --- | --- | --- | --- | --- | --- | --- | --- | --- | --- | --- | --- | --- | --- |
|  | **P-value** | **FDR P-value** | **β-coefficient** | **P-value** | | **FDR P-value** | **β-coefficient** | **P-value** | | **FDR P-value** | **β-coefficient** | **P-value** | | **FDR P-value** | **β-coefficient** |
| Vision | 0.136 | 0.273 | 0.012 | 0.074 | | 0.273 | 0.014 | 0.267 | | 0.356 | 0.009 | 0.578 | | 0.578 | -0.005 |
| Voices | 0.990 | 0.990 | -1.024E-04 | 0.557 | | 0.792 | 0.005 | 0.594 | | 0.792 | 0.004 | 0.288 | | 0.792 | 0.009 |
| Communication | 0.900 | 0.962 | 0.001 | 0.579 | | 0.962 | 0.004 | 0.962 | | 0.962 | 0.001 | 0.386 | | 0.962 | -0.007 |
| Conspiracy | 0.074 | 0.148 | 0.014 | 0.020 | | 0.081 | 0.019 | 0.318 | | 0.424 | -0.008 | 0.537 | | 0.537 | 0.005 |
| Any PE | 0.363 | 0.686 | 0.007 | 0.148 | | 0.594 | 0.012 | 0.603 | | 0.686 | 0.004 | 0.686 | | 0.686 | -0.003 |

Table 57: Effect of PE on global white matter integrity and subsets of fractional anisotropy

| Brain Structure | P-value | FDR P-Value | β-coefficient |
| --- | --- | --- | --- |
| Acoustic Radiation | 0.998 | 0.998 | 0.040 |
| Anterior Thalamic Radiation | 0.963 | 0.998 | -0.046 |
| Cingulate gyrus – part of cingulum | 0.270 | 0.622 | 0.001 |
| Parahippocampal part of cingulum | 0.823 | 0.998 | -0.001 |
| Corticospinal Tract | 0.363 | 0.622 | 0.019 |
| Inferior fronto-occipital fasciculus | 0.345 | 0.622 | -0.020 |
| Inferior longitudinal fasciculus | 0.084 | 0.622 | -0.089 |
| Medial Lemniscus | 0.766 | 0.998 | 0.022 |
| Posterior Thalamic Radiation | 0.115 | 0.622 | -0.081 |
| Superior Longitudinal fasciculus | 0.297 | 0.622 | -0.040 |
| Superior Thalamic Radiation | 0.216 | 0.622 | 0.000 |
| Uncinate Fasciculus | 0.941 | 0.998 | -0.067 |
| Forceps Major | 0.998 | 0.998 | 0.040 |
| Forceps Minor | 0.963 | 0.998 | -0.046 |
| Middle Cerebellar Peduncle | 0.270 | 0.622 | 0.001 |

Table 58: Interaction effect between hemisphere and any PE on FA values

| Brain Structure | P-value | FDR P-Value | β-coefficient |
| --- | --- | --- | --- |
| Acoustic Radiation | 0.430 | 0.957 | 0.054 |
| Anterior Thalamic Radiation | 0.177 | 0.871 | 0.099 |
| Cingulate gyrus – part of cingulum | 0.290 | 0.871 | -0.065 |
| Parahippocampal part of cingulum | 0.977 | 0.978 | -0.002 |
| Corticospinal Tract | 0.558 | 0.957 | 0.043 |
| Inferior fronto-occipital fasciculus | 0.978 | 0.978 | 0.002 |
| Inferior longitudinal fasciculus | 0.776 | 0.957 | -0.020 |
| Medial Lemniscus | 0.247 | 0.871 | 0.079 |
| Posterior Thalamic Radiation | 0.798 | 0.957 | 0.019 |
| Superior Longitudinal fasciculus | 0.739 | 0.957 | 0.023 |
| Superior Thalamic Radiation | 0.128 | 0.871 | 0.116 |
| Uncinate Fasciculus | 0.660 | 0.957 | 0.031 |
| Forceps Major | 0.430 | 0.957 | 0.054 |
| Forceps Minor | 0.177 | 0.871 | 0.099 |
| Middle Cerebellar Peduncle | 0.290 | 0.871 | -0.065 |

Table 59: Interaction effect between sex and any PE on FA values

| Brain Structure | P-value | FDR P-Value | β-coefficient |
| --- | --- | --- | --- |
| Acoustic Radiation | 0.981 | 0.981 | 0.002 |
| Anterior Thalamic Radiation | 0.444 | 0.931 | 0.057 |
| Cingulate gyrus – part of cingulum | 0.788 | 0.981 | 0.017 |
| Parahippocampal part of cingulum | 0.354 | 0.931 | 0.064 |
| Corticospinal Tract | 0.575 | 0.931 | 0.042 |
| Inferior fronto-occipital fasciculus | 0.433 | 0.931 | 0.058 |
| Inferior longitudinal fasciculus | 0.588 | 0.931 | 0.040 |
| Medial Lemniscus | 0.946 | 0.981 | 0.005 |
| Posterior Thalamic Radiation | 0.128 | 0.931 | 0.112 |
| Superior Longitudinal fasciculus | 0.621 | 0.931 | -0.035 |
| Superior Thalamic Radiation | 0.758 | 0.981 | 0.024 |
| Uncinate Fasciculus | 0.476 | 0.931 | 0.051 |
| Forceps Major | 0.024 | 0.363 | -0.026 |
| Forceps Minor | 0.958 | 0.981 | -0.006 |
| Middle Cerebellar Peduncle | 0.607 | 0.931 | 0.001 |

Table 60: Interaction effect of any PE and depression on FA values

| Brain Structure | P-value | FDR P-Value | β-coefficient [95% C.I.] |
| --- | --- | --- | --- |
| Acoustic Radiation | 0.191 | 0.357 | -0.070 [-0.174;0.035] |
| Anterior Thalamic Radiation | 0.411 | 0.440 | -0.047 [-0.158;0.065] |
| Cingulate gyrus – part of cingulum | 0.013 | 0.049 | -0.119 [-0.213;-0.025] |
| Parahippocampal part of cingulum | 0.606 | 0.606 | -0.027 [-0.132;0.077] |
| Corticospinal Tract | 0.355 | 0.410 | -0.053 [-0.165;0.059] |
| Inferior fronto-occipital fasciculus | 0.039 | 0.096 | -0.116 [-0.226;-0.006] |
| Inferior longitudinal fasciculus * | 0.004 | 0.049 | -0.163 [-0.273;-0.053] |
| Medial Lemniscus | 0.242 | 0.368 | -0.062 [-0.165;0.042] |
| Posterior Thalamic Radiation * | 0.014 | 0.049 | -0.138 [-0.248;-0.028] |
| Superior Longitudinal fasciculus * | 0.016 | 0.049 | -0.128 [-0.233;-0.023] |
| Superior Thalamic Radiation * | 0.273 | 0.373 | -0.065 [-0.181;0.051] |
| Uncinate Fasciculus | 0.348 | 0.410 | -0.051 [-0.157;0.055] |
| Forceps Major * | 0.010 | 0.049 | -0.022 [-0.025;-0.018] |
| Forceps Minor | 0.245 | 0.368 | -0.010 [-0.012;-0.007] |
| Middle Cerebellar Peduncle | 0.126 | 0.270 | -0.013[-0.017;-0.009] |

Table 61: Association between experiencing any of the PEs and FA values in white matter tracts with only subjects who found the PE distressful (N=276). CI = Confidence Interval, FDR = False Discovery Rate, * = p_corrected_ <0.05

### Visions

| Subset | P-value | FDR P-value | β-coefficient |
| --- | --- | --- | --- |
| General MD | 0.136 | 0.273 | 0.012 |
| Association fibres | 0.074 | 0.273 | 0.014 |
| Thalamic fibres | 0.267 | 0.356 | 0.009 |
| Sensory fibres | 0.578 | 0.578 | -0.005 |

Table 62: Effect of 'Visions' on subsets white matter tracts

| Brain Structure | P-value | FDR P-Value | β-coefficient |
| --- | --- | --- | --- |
| Acoustic Radiation | 0.412 | 0.824 | 0.014 |
| Anterior Thalamic Radiation | 0.788 | 0.977 | -0.051 |
| Cingulate gyrus – part of cingulum | 0.197 | 0.592 | 0.037 |
| Parahippocampal part of cingulum | 0.844 | 0.977 | -0.038 |
| Corticospinal Tract | 0.970 | 0.977 | 0.031 |
| Inferior fronto-occipital fasciculus | 0.315 | 0.755 | -0.012 |
| Inferior longitudinal fasciculus | 0.035 | 0.365 | -0.104 |
| Medial Lemniscus | 0.542 | 0.930 | 0.094 |
| Posterior Thalamic Radiation | 0.061 | 0.365 | -0.125 |
| Superior Longitudinal fasciculus | 0.153 | 0.592 | -0.102 |
| Superior Thalamic Radiation | 0.729 | 0.977 | -0.031 |
| Uncinate Fasciculus | 0.977 | 0.977 | -0.098 |

Table 63: Interaction effect between hemisphere and ‘Visions’ PE on FA values

| Brain Structure | P-value | FDR P-Value | β-coefficient |
| --- | --- | --- | --- |
| Acoustic Radiation | 0.814 | 0.935 | 0.021 |
| Anterior Thalamic Radiation | 0.477 | 0.935 | 0.068 |
| Cingulate gyrus – part of cingulum | 0.115 | 0.935 | -0.126 |
| Parahippocampal part of cingulum | 0.772 | 0.935 | 0.026 |
| Corticospinal Tract | 0.857 | 0.935 | 0.017 |
| Inferior fronto-occipital fasciculus | 0.966 | 0.966 | -0.004 |
| Inferior longitudinal fasciculus | 0.554 | 0.935 | -0.055 |
| Medial Lemniscus | 0.191 | 0.935 | 0.116 |
| Posterior Thalamic Radiation | 0.745 | 0.935 | -0.031 |
| Superior Longitudinal fasciculus | 0.696 | 0.935 | -0.035 |
| Superior Thalamic Radiation | 0.499 | 0.935 | 0.067 |
| Uncinate Fasciculus | 0.803 | 0.935 | 0.023 |

Table 64: Interaction effect between sex and ‘Visions’ PE on FA values

| Brain Structure | P-value | FDR P-Value | β-coefficient |
| --- | --- | --- | --- |
| Acoustic Radiation | 0.741 | 0.971 | -0.029 |
| Anterior Thalamic Radiation | 0.714 | 0.971 | 0.034 |
| Cingulate gyrus – part of cingulum | 0.798 | 0.971 | 0.020 |
| Parahippocampal part of cingulum | 0.407 | 0.867 | 0.072 |
| Corticospinal Tract | 0.347 | 0.867 | -0.088 |
| Inferior fronto-occipital fasciculus | 0.938 | 0.971 | 0.007 |
| Inferior longitudinal fasciculus | 0.950 | 0.971 | -0.006 |
| Medial Lemniscus | 0.158 | 0.867 | -0.122 |
| Posterior Thalamic Radiation | 0.316 | 0.867 | 0.092 |
| Superior Longitudinal fasciculus | 0.337 | 0.867 | -0.084 |
| Superior Thalamic Radiation | 0.355 | 0.867 | -0.090 |
| Uncinate Fasciculus | 0.838 | 0.971 | 0.018 |
| Forceps Major * | 0.001 | 0.018 | -0.029 |
| Forceps Minor | 0.463 | 0.867 | -0.008 |
| Middle Cerebellar Peduncle | 0.971 | 0.971 | 0.000 |

Table 65: Interaction effect of 'visions' PE and depression on FA values

| Brain Structure | P-value | FDR P-Value | β-coefficient |
| --- | --- | --- | --- |
| Acoustic Radiation | 0.434 | 0.814 | -0.001 |
| Anterior Thalamic Radiation | 0.853 | 0.958 | 0.000 |
| Cingulate gyrus – part of cingulum | 0.204 | 0.611 | -0.002 |
| Parahippocampal part of cingulum | 0.834 | 0.958 | 0.000 |
| Corticospinal Tract | 0.883 | 0.958 | 0.000 |
| Inferior fronto-occipital fasciculus | 0.389 | 0.814 | -0.001 |
| Inferior longitudinal fasciculus | 0.053 | 0.394 | -0.002 |
| Medial Lemniscus | 0.631 | 0.958 | -0.001 |
| Posterior Thalamic Radiation | 0.079 | 0.394 | -0.002 |
| Superior Longitudinal fasciculus | 0.170 | 0.611 | -0.001 |
| Superior Thalamic Radiation | 0.644 | 0.958 | 0.000 |
| Uncinate Fasciculus | 0.989 | 0.989 | 0.000 |
| Forceps Major * | 0.001 | 0.009 | -0.029 |
| Forceps Minor | 0.396 | 0.814 | -0.029 |
| Middle Cerebellar Peduncle | 0.894 | 0.958 | -0.029 |

Table 66: Effect of ‘Visions’ PE on FA values with cannabis use as covariate

| Brain Structure | P-value | FDR P-Value | β-coefficient |
| --- | --- | --- | --- |
| Acoustic Radiation | 0.166 | 0.499 | -0.089 |
| Anterior Thalamic Radiation | 0.616 | 0.793 | -0.035 |
| Cingulate gyrus – part of cingulum | 0.314 | 0.562 | -0.061 |
| Parahippocampal part of cingulum | 0.889 | 0.889 | 0.010 |
| Corticospinal Tract | 0.306 | 0.562 | -0.067 |
| Inferior fronto-occipital fasciculus | 0.337 | 0.562 | -0.066 |
| Inferior longitudinal fasciculus | 0.084 | 0.499 | -0.122 |
| Medial Lemniscus | 0.740 | 0.793 | -0.022 |
| Posterior Thalamic Radiation | 0.142 | 0.499 | -0.102 |
| Superior Longitudinal fasciculus | 0.125 | 0.499 | -0.108 |
| Superior Thalamic Radiation | 0.295 | 0.562 | -0.080 |
| Uncinate Fasciculus | 0.728 | 0.793 | -0.024 |
| Forceps Major * | **0.001** | **0.013** | **-0.022** |
| Forceps Minor | 0.715 | 0.793 | 0.002 |
| Middle Cerebellar Peduncle | 0.453 | 0.679 | 0.006 |

Table 67: Effect of ‘Visions’ PE on FA values with antipsychotic medication use as covariate

| Brain Structure | P-value | FDR P-Value | β-coefficient [95% CI] |
| --- | --- | --- | --- |
| Acoustic Radiation | 0.413 | 0.774 | -0.035 |
| Anterior Thalamic Radiation | 0.737 | 0.978 | -0.015 |
| Cingulate gyrus – part of cingulum | 0.197 | 0.590 | -0.050 |
| Parahippocampal part of cingulum | 0.978 | 0.978 | 0.001 |
| Corticospinal Tract | 0.943 | 0.978 | -0.003 |
| Inferior fronto-occipital fasciculus | 0.268 | 0.669 | -0.051 |
| Inferior longitudinal fasciculus | 0.027 | 0.204 | -0.100 |
| Medial Lemniscus | 0.563 | 0.938 | -0.025 |
| Posterior Thalamic Radiation | 0.074 | 0.371 | -0.082 |
| Superior Longitudinal fasciculus | 0.169 | 0.590 | -0.060 |
| Superior Thalamic Radiation | 0.792 | 0.978 | 0.013 |
| Uncinate Fasciculus | 0.836 | 0.978 | -0.009 |
| Forceps Major * | 0.001 | 0.009 | -0.029 |
| Forceps Minor | 0.380 | 0.774 | -0.007 |
| Middle Cerebellar Peduncle | 0.965 | 0.978 | 0.000 |

Table 68: Effect of ‘Visions’ PE on FA values excluding subjects with self-reported diagnosis of psychosis

| Brain Structure | P-value | FDR P-Value | β-coefficient [95% CI] |
| --- | --- | --- | --- |
| Acoustic Radiation | 0.037 | 0.138 | -0.152 [-0.294;-0.009] |
| Anterior Thalamic Radiation | 0.921 | 0.921 | 0.008 [-0.145;0.160] |
| Cingulate gyrus – part of cingulum | 0.117 | 0.251 | -0.103 [-0.231;0.026] |
| Parahippocampal part of cingulum | 0.803 | 0.860 | -0.018 [-0.161;0.124] |
| Corticospinal Tract | 0.428 | 0.535 | -0.062 [-0.215;0.091] |
| Inferior fronto-occipital fasciculus | 0.259 | 0.432 | -0.087 [-0.237;0.064] |
| Inferior longitudinal fasciculus | 0.022 | 0.135 | -0.176 [-0.326;-0.026] |
| Medial Lemniscus | 0.211 | 0.395 | -0.090 [-0.232;0.051] |
| Posterior Thalamic Radiation | 0.027 | 0.135 | -0.170 [-0.321;-0.019] |
| Superior Longitudinal fasciculus | 0.048 | 0.143 | -0.145 [-0.288;-0.002] |
| Superior Thalamic Radiation | 0.117 | 0.251 | -0.127 [-0.286;0.032] |
| Uncinate Fasciculus | 0.499 | 0.576 | -0.050 [-0.195;0.095] |
| Forceps Major * | 0.001 | 0.014 | -0.028 [-0.033;-0.023] |
| Forceps Minor | 0.294 | 0.442 | -0.009 [-0.012;-0.005] |
| Middle Cerebellar Peduncle | 0.410 | 0.535 | -0.007 [-0.012;-0.002] |

Table 69: Effect of ‘Visions’ PE on FA values in only distressed cases

| Brain Structure | P-value | FDR P-Value | β-coefficient with distressed group |
| --- | --- | --- | --- |
| Acoustic Radiation | 0.022 | 0.111 | -0.271 |
| Anterior Thalamic Radiation | 0.722 | 0.722 | -0.061 |
| Cingulate gyrus – part of cingulum | 0.015 | 0.111 | -0.212 |
| Parahippocampal part of cingulum | 0.147 | 0.276 | -0.116 |
| Corticospinal Tract | 0.118 | 0.276 | -0.218 |
| Inferior fronto-occipital fasciculus | 0.544 | 0.583 | -0.120 |
| Inferior longitudinal fasciculus | 0.166 | 0.276 | -0.205 |
| Medial Lemniscus | 0.129 | 0.276 | -0.202 |
| Posterior Thalamic Radiation | 0.185 | 0.278 | -0.201 |
| Superior Longitudinal fasciculus | 0.125 | 0.276 | -0.207 |
| Superior Thalamic Radiation | 0.009 | 0.111 | -0.357 |
| Uncinate Fasciculus | 0.529 | 0.583 | -0.072 |
| Forceps Major | 0.163 | 0.276 | -0.106 |
| Forceps Minor | 0.519 | 0.583 | -0.062 |
| Middle Cerebellar Peduncle | 0.426 | 0.581 | -0.073 |

Table 70: Difference in effect sizes between 'neutral', 'positive' and 'distressing' group

### Voices

| Subset | P-value | FDR P-value | β-coefficient |
| --- | --- | --- | --- |
| General MD | 0.990 | 0.990 | 0.000 |
| Association fibbers | 0.557 | 0.792 | 0.005 |
| Thalamic fibres | 0.594 | 0.792 | 0.004 |
| Sensory fibres | 0.288 | 0.792 | 0.009 |

Table 71: Effect of 'Voices' on subsets white matter tracts

| Brain Structure | P-value | FDR P-Value | β-coefficient [95% CI] |
| --- | --- | --- | --- |
| Acoustic Radiation | 0.852 | 0.972 | 0.010 [-0.096;0.116] |
| Anterior Thalamic Radiation | 0.581 | 0.896 | 0.032 [-0.081;0.145] |
| Cingulate gyrus – part of cingulum | 0.324 | 0.896 | -0.048 [-0.143;0.047] |
| Parahippocampal part of cingulum | 0.657 | 0.896 | 0.024 [-0.082;0.130] |
| Corticospinal Tract | 0.306 | 0.896 | 0.059 [-0.054;0.173] |
| Inferior fronto-occipital fasciculus | 0.952 | 0.972 | -0.003 [-0.115;0.108] |
| Inferior longitudinal fasciculus | 0.630 | 0.896 | -0.027 [-0.139;0.084] |
| Medial Lemniscus | 0.585 | 0.896 | 0.029 [-0.076;0.134] |
| Posterior Thalamic Radiation | 0.547 | 0.896 | -0.034 [-0.146;0.077] |
| Superior Longitudinal fasciculus | 0.417 | 0.896 | -0.044 [-0.150;0.062] |
| Superior Thalamic Radiation | 0.173 | 0.896 | 0.082 [-0.036;0.200] |
| Uncinate Fasciculus | 0.972 | 0.972 | -0.002 [-0.110;0.106] |
| Forceps Major | 0.242 | 0.896 | -0.010 [-0.006;0.001] |
| Forceps Minor | 0.841 | 0.972 | -0.002 [-0.003;0.002] |
| Middle Cerebellar Peduncle | 0.128 | 0.896 | 0.013 [-0.001;0.007] |

Table 72: Effect of ‘Voices’ PE on FA values

| Brain Structure | P-value | FDR P-Value | β-coefficient |
| --- | --- | --- | --- |
| Acoustic Radiation | 0.852 | 0.972 | 0.113 |
| Anterior Thalamic Radiation | 0.581 | 0.876 | -0.085 |
| Cingulate gyrus – part of cingulum | 0.324 | 0.876 | -0.053 |
| Parahippocampal part of cingulum | 0.657 | 0.876 | 0.148 |
| Corticospinal Tract | 0.306 | 0.876 | 0.027 |
| Inferior fronto-occipital fasciculus | 0.952 | 0.972 | -0.041 |
| Inferior longitudinal fasciculus | 0.630 | 0.876 | -0.119 |
| Medial Lemniscus | 0.585 | 0.876 | 0.057 |
| Posterior Thalamic Radiation | 0.547 | 0.876 | -0.098 |
| Superior Longitudinal fasciculus | 0.417 | 0.876 | 0.003 |
| Superior Thalamic Radiation | 0.173 | 0.876 | -0.018 |
| Uncinate Fasciculus | 0.972 | 0.972 | -0.079 |

Table 73: Interaction effect between hemisphere and ‘Voices’ PE on FA values

| Brain Structure | P-value | FDR P-Value | β-coefficient |
| --- | --- | --- | --- |
| Acoustic Radiation | 0.318 | 0.692 | 0.108 |
| Anterior Thalamic Radiation | 0.549 | 0.692 | 0.070 |
| Cingulate gyrus – part of cingulum | 0.577 | 0.692 | -0.055 |
| Parahippocampal part of cingulum | 0.475 | 0.692 | 0.078 |
| Corticospinal Tract | 0.487 | 0.692 | 0.081 |
| Inferior fronto-occipital fasciculus | 0.519 | 0.692 | -0.074 |
| Inferior longitudinal fasciculus | 0.210 | 0.692 | -0.143 |
| Medial Lemniscus | 0.886 | 0.886 | 0.016 |
| Posterior Thalamic Radiation | 0.417 | 0.692 | -0.093 |
| Superior Longitudinal fasciculus | 0.877 | 0.886 | 0.017 |
| Superior Thalamic Radiation | 0.352 | 0.692 | 0.113 |
| Uncinate Fasciculus | 0.522 | 0.692 | 0.071 |

Table 74: Interaction effect between sex and ‘Voices’ PE on FA values

| Brain Structure | P-value | FDR P-Value | β-coefficient |
| --- | --- | --- | --- |
| Acoustic Radiation | 0.436 | 0.826 | -0.084 |
| Anterior Thalamic Radiation | 0.841 | 0.916 | 0.023 |
| Cingulate gyrus – part of cingulum | 0.495 | 0.826 | 0.067 |
| Parahippocampal part of cingulum | 0.458 | 0.826 | 0.081 |
| Corticospinal Tract | 0.422 | 0.826 | 0.094 |
| Inferior fronto-occipital fasciculus | 0.674 | 0.916 | 0.048 |
| Inferior longitudinal fasciculus | 0.860 | 0.916 | -0.020 |
| Medial Lemniscus | 0.140 | 0.826 | 0.159 |
| Posterior Thalamic Radiation | 0.659 | 0.916 | 0.051 |
| Superior Longitudinal fasciculus | 0.321 | 0.826 | -0.108 |
| Superior Thalamic Radiation | 0.913 | 0.916 | 0.013 |
| Uncinate Fasciculus | 0.345 | 0.826 | 0.104 |
| Forceps Major | 0.431 | 0.826 | -0.006 |
| Forceps Minor | 0.916 | 0.916 | -0.002 |
| Middle Cerebellar Peduncle | 0.132 | 0.826 | 0.013 |

Table 75: Interaction effect of 'voices'' PE and depression on FA values

### Communications

| Subset | P-value | FDR P-value | Β-coefficient |
| --- | --- | --- | --- |
| General MD | 0.900 | 0.962 | 0.001 |
| Association fibres | 0.579 | 0.962 | 0.004 |
| Thalamic fibres | 0.962 | 0.962 | 0.000 |
| Sensory fibres | 0.386 | 0.962 | -0.007 |

Table 76: Effect of 'Communications' on subsets white matter tracts

| Brain Structure | P-value | FDR P-Value | β-coefficient [95% CI] |
| --- | --- | --- | --- |
| Acoustic Radiation | 0.959 | 0.995 | -0.005 [-0.177;0.168] |
| Anterior Thalamic Radiation | 0.663 | 0.995 | 0.041 [-0.143;0.226] |
| Cingulate gyrus – part of cingulum | 0.590 | 0.995 | -0.043 [-0.198;0.113] |
| Parahippocampal part of cingulum | 0.373 | 0.995 | -0.079 [-0.251;0.094] |
| Corticospinal Tract | 0.440 | 0.995 | 0.073 [-0.112;0.259] |
| Inferior fronto-occipital fasciculus | 0.995 | 0.995 | -0.001 [-0.183;0.182] |
| Inferior longitudinal fasciculus | 0.596 | 0.995 | -0.049 [-0.231;0.133] |
| Medial Lemniscus | 0.694 | 0.995 | -0.034 [-0.206;0.137] |
| Posterior Thalamic Radiation | 0.924 | 0.995 | -0.009 [-0.192;0.174] |
| Superior Longitudinal fasciculus | 0.853 | 0.995 | -0.016 [-0.190;0.157] |
| Superior Thalamic Radiation | 0.946 | 0.995 | 0.007 [-0.186;0.199] |
| Uncinate Fasciculus | 0.631 | 0.995 | 0.043 [-0.133;0.219] |
| Forceps Major | 0.530 | 0.995 | -0.005 [-0.008;0.004] |
| Forceps Minor | 0.449 | 0.995 | 0.006 [-0.003;0.006] |
| Middle Cerebellar Peduncle | 0.064 | 0.966 | 0.015 [0.000;0.012] |

Table 77: Effect of 'communications' PE on FA values

| Brain Structure | P-value | FDR P-Value | β-coefficient |
| --- | --- | --- | --- |
| Acoustic Radiation | 0.959 | 0.995 | -0.102 |
| Anterior Thalamic Radiation | 0.663 | 0.995 | -0.016 |
| Cingulate gyrus – part of cingulum | 0.590 | 0.995 | 0.009 |
| Parahippocampal part of cingulum | 0.373 | 0.995 | 0.100 |
| Corticospinal Tract | 0.440 | 0.995 | -0.053 |
| Inferior fronto-occipital fasciculus | 0.995 | 0.995 | -0.122 |
| Inferior longitudinal fasciculus | 0.596 | 0.995 | -0.138 |
| Medial Lemniscus | 0.694 | 0.995 | -0.284 |
| Posterior Thalamic Radiation | 0.924 | 0.995 | -0.033 |
| Superior Longitudinal fasciculus | 0.853 | 0.995 | -0.023 |
| Superior Thalamic Radiation | 0.946 | 0.995 | -0.099 |
| Uncinate Fasciculus | 0.631 | 0.995 | 0.011 |

Table 78: Interaction effect between hemisphere and 'communications' PE on FA values

| Brain Structure | P-value | FDR P-Value | β-coefficient |
| --- | --- | --- | --- |
| Acoustic Radiation | 0.218 | 0.430 | 0.217 |
| Anterior Thalamic Radiation | 0.112 | 0.430 | 0.300 |
| Cingulate gyrus – part of cingulum | 0.552 | 0.552 | -0.094 |
| Parahippocampal part of cingulum | 0.202 | 0.430 | 0.225 |
| Corticospinal Tract | 0.083 | 0.430 | 0.329 |
| Inferior fronto-occipital fasciculus | 0.375 | 0.443 | 0.166 |
| Inferior longitudinal fasciculus | 0.406 | 0.443 | 0.154 |
| Medial Lemniscus | 0.240 | 0.430 | 0.205 |
| Posterior Thalamic Radiation | 0.251 | 0.430 | 0.214 |
| Superior Longitudinal fasciculus | 0.312 | 0.443 | 0.179 |
| Superior Thalamic Radiation | 0.035 | 0.416 | 0.415 |
| Uncinate Fasciculus | 0.397 | 0.443 | 0.152 |

Table 79: Interaction effect between sex and 'communications' PE on FA values

| Brain Structure | P-value | FDR P-Value | β-coefficient |
| --- | --- | --- | --- |
| Acoustic Radiation | 0.314 | 0.762 | -0.178 |
| Anterior Thalamic Radiation | 0.282 | 0.762 | 0.204 |
| Cingulate gyrus – part of cingulum | 0.356 | 0.762 | -0.147 |
| Parahippocampal part of cingulum | 0.102 | 0.761 | -0.290 |
| Corticospinal Tract | 0.181 | 0.762 | 0.255 |
| Inferior fronto-occipital fasciculus | 0.711 | 0.892 | 0.069 |
| Inferior longitudinal fasciculus | 0.747 | 0.892 | -0.060 |
| Medial Lemniscus | 0.731 | 0.892 | -0.060 |
| Posterior Thalamic Radiation | 0.773 | 0.892 | -0.054 |
| Superior Longitudinal fasciculus | 0.673 | 0.892 | -0.075 |
| Superior Thalamic Radiation | 0.956 | 0.956 | 0.011 |
| Uncinate Fasciculus | 0.615 | 0.892 | -0.091 |
| Forceps Major* | 0.876 | 0.939 | 0.006 |
| Forceps Minor | 0.241 | 0.762 | 0.012 |
| Middle Cerebellar Peduncle | 0.034 | 0.516 | 0.011 |

Table 80: Interaction effect of 'communications' PE and depression on FA values

### Conspiracies

| Subset | P-value | FDR P-value | Β-coefficient |
| --- | --- | --- | --- |
| General MD | 0.074 | 0.148 | 0.014 |
| Association fibres | 0.020 | 0.081 | 0.019 |
| Thalamic fibres | 0.318 | 0.424 | -0.008 |
| Sensory fibres | 0.537 | 0.537 | 0.005 |

Table 81: Effect of 'Conspiracies' on FA scores of subsets white matter tracts

| Brain Structure | P-value | FDR P-Value | β-coefficient [95% CI] |
| --- | --- | --- | --- |
| Acoustic Radiation | 0.772 | 0.827 | -0.024 [-0.187;0.139] |
| Anterior Thalamic Radiation | 0.372 | 0.533 | -0.079 [-0.253;0.095] |
| Cingulate gyrus – part of cingulum | 0.020 | 0.099 | -0.174 [-0.321;-0.028] |
| Parahippocampal part of cingulum | 0.487 | 0.562 | -0.058 [-0.221;0.105] |
| Corticospinal Tract | 0.891 | 0.891 | 0.012 [-0.163;0.187] |
| Inferior fronto-occipital fasciculus | 0.018 | 0.099 | -0.209 [-0.381;-0.037] |
| Inferior longitudinal fasciculus | 0.018 | 0.099 | -0.207 [-0.378;-0.035] |
| Medial Lemniscus | 0.297 | 0.513 | -0.086 [-0.247;0.076] |
| Posterior Thalamic Radiation | 0.066 | 0.247 | -0.162 [-0.334;0.011] |
| Superior Longitudinal fasciculus | 0.209 | 0.448 | -0.105 [-0.269;0.059] |
| Superior Thalamic Radiation | 0.466 | 0.562 | 0.068 [-0.114;0.249] |
| Uncinate Fasciculus | 0.127 | 0.317 | -0.129 [-0.295;0.037] |
| Forceps Major | 0.113 | 0.317 | -0.013 [-0.010;0.001] |
| Forceps Minor | 0.308 | 0.513 | -0.008 [-0.006;0.002] |
| Middle Cerebellar Peduncle | 0.391 | 0.533 | -0.007 [-0.008;0.003] |

Table 82: Effect of 'conspiracies' PE on FA values

| Brain Structure | P-value | FDR P-Value | β-coefficient |
| --- | --- | --- | --- |
| Acoustic Radiation | 0.772 | 0.842 | -0.096 |
| Anterior Thalamic Radiation | 0.372 | 0.557 | -0.062 |
| Cingulate gyrus – part of cingulum | 0.020 | 0.079 | -0.231 |
| Parahippocampal part of cingulum | 0.487 | 0.585 | -0.252 |
| Corticospinal Tract | 0.891 | 0.891 | -0.079 |
| Inferior fronto-occipital fasciculus | 0.018 | 0.079 | -0.162 |
| Inferior longitudinal fasciculus | 0.018 | 0.079 | -0.182 |
| Medial Lemniscus | 0.297 | 0.509 | -0.276 |
| Posterior Thalamic Radiation | 0.066 | 0.197 | -0.086 |
| Superior Longitudinal fasciculus | 0.209 | 0.418 | -0.207 |
| Superior Thalamic Radiation | 0.466 | 0.585 | 0.023 |
| Uncinate Fasciculus | 0.127 | 0.305 | -0.005 |

Table 83: Interaction effect between hemisphere and 'conspiracies' PE

| Brain Structure | P-value | FDR P-Value | β-coefficient |
| --- | --- | --- | --- |
| Acoustic Radiation | 0.135 | 0.544 | -0.249 |
| Anterior Thalamic Radiation | 0.737 | 0.737 | -0.060 |
| Cingulate gyrus – part of cingulum | 0.529 | 0.635 | -0.095 |
| Parahippocampal part of cingulum | 0.507 | 0.635 | -0.111 |
| Corticospinal Tract | 0.107 | 0.544 | -0.290 |
| Inferior fronto-occipital fasciculus | 0.305 | 0.544 | -0.181 |
| Inferior longitudinal fasciculus | 0.317 | 0.544 | -0.176 |
| Medial Lemniscus | 0.172 | 0.544 | -0.226 |
| Posterior Thalamic Radiation | 0.253 | 0.544 | -0.202 |
| Superior Longitudinal fasciculus | 0.412 | 0.618 | -0.138 |
| Superior Thalamic Radiation | 0.624 | 0.681 | -0.091 |
| Uncinate Fasciculus | 0.270 | 0.544 | -0.188 |

Table 84: Interaction effect of 'conspiracy' PE and sex on FA values

| Brain Structure | P-value | FDR P-Value | β-coefficient |
| --- | --- | --- | --- |
| Acoustic Radiation | 0.260 | 0.434 | 0.190 |
| Anterior Thalamic Radiation | 0.011 | 0.083 | 0.457 |
| Cingulate gyrus – part of cingulum | 0.317 | 0.436 | 0.152 |
| Parahippocampal part of cingulum | 0.663 | 0.663 | 0.074 |
| Corticospinal Tract | 0.143 | 0.307 | 0.265 |
| Inferior fronto-occipital fasciculus | 0.007 | 0.083 | 0.482 |
| Inferior longitudinal fasciculus | 0.021 | 0.083 | 0.409 |
| Medial Lemniscus | 0.602 | 0.645 | 0.087 |
| Posterior Thalamic Radiation | 0.022 | 0.083 | 0.408 |
| Superior Longitudinal fasciculus | 0.082 | 0.230 | 0.295 |
| Superior Thalamic Radiation | 0.196 | 0.368 | 0.243 |
| Uncinate Fasciculus | 0.092 | 0.230 | 0.290 |
| Forceps Major | 0.320 | 0.436 | -0.025 |
| Forceps Minor | 0.494 | 0.570 | -0.017 |
| Middle Cerebellar Peduncle | 0.369 | 0.461 | -0.028 |

Table 85: Interaction effect of 'conspiracy' PE and depression on FA values

## Mean Diffusivity

### Any PE

| Subset | P-value | FDR P-value | β-coefficient |
| --- | --- | --- | --- |
| General MD | 0.242 | 0.388 | -0.009 |
| Association fibres | 0.128 | 0.388 | -0.012 |
| Thalamic fibres | 0.291 | 0.388 | -0.008 |
| Sensory fibres | 0.997 | 0.997 | 0.000 |

Table 86: Effect of 'Any PE' on subsets white matter tracts

| Brain Structure | P-value | FDR P-Value | β-coefficient |
| --- | --- | --- | --- |
| Acoustic Radiation | 0.694 | 0.771 | -0.013 |
| Anterior Thalamic Radiation | 0.224 | 0.690 | 0.041 |
| Cingulate gyrus – part of cingulum | 0.279 | 0.690 | 0.039 |
| Parahippocampal part of cingulum | 0.149 | 0.690 | 0.049 |
| Corticospinal Tract | 0.521 | 0.771 | -0.024 |
| Inferior fronto-occipital fasciculus | 0.253 | 0.690 | 0.041 |
| Inferior longitudinal fasciculus | 0.173 | 0.690 | 0.047 |
| Medial Lemniscus | 0.692 | 0.771 | -0.013 |
| Posterior Thalamic Radiation | 0.439 | 0.732 | 0.026 |
| Superior Longitudinal fasciculus | 0.322 | 0.690 | 0.035 |
| Superior Thalamic Radiation | 0.720 | 0.771 | 0.012 |
| Uncinate Fasciculus | 0.261 | 0.690 | 0.036 |
| Forceps Major | 0.429 | 0.732 | 0.006 |
| Forceps Minor | 0.963 | 0.963 | 0.000 |
| Middle Cerebellar Peduncle | 0.604 | 0.771 | 0.004 |

Table 87: Effect of any PE on MD values of white matter tracts

| Brain Structure | P-value | FDR P-Value | β-coefficient |
| --- | --- | --- | --- |
| Acoustic Radiation | 0.694 | 0.720 | 0.012 |
| Anterior Thalamic Radiation | 0.224 | 0.552 | 0.080 |
| Cingulate gyrus – part of cingulum | 0.279 | 0.552 | 0.016 |
| Parahippocampal part of cingulum | 0.149 | 0.552 | 0.020 |
| Corticospinal Tract | 0.521 | 0.695 | -0.003 |
| Inferior fronto-occipital fasciculus | 0.253 | 0.552 | 0.044 |
| Inferior longitudinal fasciculus | 0.173 | 0.552 | 0.038 |
| Medial Lemniscus | 0.692 | 0.720 | -0.081 |
| Posterior Thalamic Radiation | 0.439 | 0.659 | 0.039 |
| Superior Longitudinal fasciculus | 0.322 | 0.552 | 0.017 |
| Superior Thalamic Radiation | 0.720 | 0.720 | 0.021 |
| Uncinate Fasciculus | 0.261 | 0.552 | 0.051 |

Table 88: Interaction effect between hemisphere and any PE on MD values

| Brain Structure | P-value | FDR P-Value | β-coefficient |
| --- | --- | --- | --- |
| Acoustic Radiation | 0.563 | 0.967 | -0.040 |
| Anterior Thalamic Radiation | 0.870 | 0.967 | -0.011 |
| Cingulate gyrus – part of cingulum | 0.834 | 0.967 | 0.015 |
| Parahippocampal part of cingulum | 0.515 | 0.967 | 0.045 |
| Corticospinal Tract | 0.831 | 0.967 | 0.016 |
| Inferior fronto-occipital fasciculus | 0.983 | 0.983 | 0.002 |
| Inferior longitudinal fasciculus | 0.842 | 0.967 | 0.014 |
| Medial Lemniscus | 0.750 | 0.967 | 0.022 |
| Posterior Thalamic Radiation | 0.850 | 0.967 | 0.013 |
| Superior Longitudinal fasciculus | 0.658 | 0.967 | -0.032 |
| Superior Thalamic Radiation | 0.545 | 0.967 | -0.041 |
| Uncinate Fasciculus | 0.886 | 0.967 | -0.009 |

Table 89: Interaction effect between sex and any PE on MD values

| Brain Structure | P-value | FDR P-Value | β-coefficient |
| --- | --- | --- | --- |
| Acoustic Radiation | 0.921 | 0.946 | 0.007 |
| Anterior Thalamic Radiation | 0.484 | 0.879 | -0.049 |
| Cingulate gyrus – part of cingulum | 0.778 | 0.946 | -0.021 |
| Parahippocampal part of cingulum | 0.341 | 0.852 | -0.066 |
| Corticospinal Tract | 0.108 | 0.538 | -0.123 |
| Inferior fronto-occipital fasciculus | 0.202 | 0.605 | -0.094 |
| Inferior longitudinal fasciculus | 0.144 | 0.542 | -0.105 |
| Medial Lemniscus | 0.044 | 0.538 | -0.140 |
| Posterior Thalamic Radiation | 0.101 | 0.538 | -0.115 |
| Superior Longitudinal fasciculus | 0.774 | 0.946 | 0.021 |
| Superior Thalamic Radiation | 0.442 | 0.879 | -0.053 |
| Uncinate Fasciculus | 0.691 | 0.946 | -0.026 |
| Forceps Major | 0.528 | 0.879 | 0.009 |
| Forceps Minor | 0.946 | 0.946 | 0.003 |
| Middle Cerebellar Peduncle | 0.851 | 0.946 | -0.006 |

Table 90: Interaction effect between depression and any PE on MD values

### Visions

| Subset | P-value | FDR P-value | Β-coefficient |
| --- | --- | --- | --- |
| General MD | 0.278 | 0.487 | -0.008 |
| Association fibres | 0.171 | 0.487 | -0.011 |
| Thalamic fibres | 0.365 | 0.487 | -0.007 |
| Sensory fibres | 0.993 | 0.993 | 0.000 |

Table 91: Effect of 'Visions' on MD values of subsets white matter tracts

| Brain Structure | P-value | FDR P-Value | β-coefficient |
| --- | --- | --- | --- |
| Acoustic Radiation | 0.841 | 0.970 | -0.009 |
| Anterior Thalamic Radiation | 0.274 | 0.684 | 0.047 |
| Cingulate gyrus – part of cingulum | 0.476 | 0.793 | 0.032 |
| Parahippocampal part of cingulum | 0.465 | 0.793 | 0.031 |
| Corticospinal Tract | 0.986 | 0.986 | 0.001 |
| Inferior fronto-occipital fasciculus | 0.249 | 0.684 | 0.052 |
| Inferior longitudinal fasciculus | 0.177 | 0.684 | 0.059 |
| Medial Lemniscus | 0.831 | 0.970 | -0.009 |
| Posterior Thalamic Radiation | 0.660 | 0.970 | 0.019 |
| Superior Longitudinal fasciculus | 0.183 | 0.684 | 0.060 |
| Superior Thalamic Radiation | 0.381 | 0.793 | 0.037 |
| Uncinate Fasciculus | 0.184 | 0.684 | 0.053 |
| Forceps Major* | 0.080 | 0.684 | 0.014 |
| Forceps Minor | 0.967 | 0.986 | 0.000 |
| Middle Cerebellar Peduncle | 0.822 | 0.970 | 0.002 |

Table 92: Effect of Visions on MD values of white matter tracts

| Brain Structure | P-value | FDR P-Value | β-coefficient |
| --- | --- | --- | --- |
| Acoustic Radiation | 0.841 | 0.917 | 0.030 |
| Anterior Thalamic Radiation | 0.274 | 0.657 | 0.068 |
| Cingulate gyrus – part of cingulum | 0.476 | 0.714 | -0.011 |
| Parahippocampal part of cingulum | 0.465 | 0.714 | -0.023 |
| Corticospinal Tract | 0.986 | 0.986 | 0.015 |
| Inferior fronto-occipital fasciculus | 0.249 | 0.657 | 0.042 |
| Inferior longitudinal fasciculus | 0.177 | 0.657 | 0.054 |
| Medial Lemniscus | 0.831 | 0.917 | -0.093 |
| Posterior Thalamic Radiation | 0.660 | 0.880 | 0.039 |
| Superior Longitudinal fasciculus | 0.183 | 0.657 | 0.058 |
| Superior Thalamic Radiation | 0.381 | 0.714 | 0.004 |
| Uncinate Fasciculus | 0.184 | 0.657 | 0.118 |

Table 93: Interaction effect between hemisphere and Visions on MD values

| Brain Structure | P-value | FDR P-Value | β-coefficient |
| --- | --- | --- | --- |
| Acoustic Radiation | 0.792 | 0.964 | -0.024 |
| Anterior Thalamic Radiation | 0.820 | 0.964 | -0.020 |
| Cingulate gyrus – part of cingulum | 0.344 | 0.964 | 0.089 |
| Parahippocampal part of cingulum | 0.799 | 0.964 | 0.023 |
| Corticospinal Tract | 0.316 | 0.964 | 0.098 |
| Inferior fronto-occipital fasciculus | 0.770 | 0.964 | 0.027 |
| Inferior longitudinal fasciculus | 0.633 | 0.964 | 0.044 |
| Medial Lemniscus | 0.372 | 0.964 | 0.079 |
| Posterior Thalamic Radiation | 0.924 | 0.964 | 0.009 |
| Superior Longitudinal fasciculus | 0.765 | 0.964 | 0.028 |
| Superior Thalamic Radiation | 0.964 | 0.964 | 0.004 |
| Uncinate Fasciculus | 0.935 | 0.964 | 0.007 |

Table 94: Interaction effect between sex and Visions on MD values

| Brain Structure | P-value | FDR P-Value | β-coefficient |
| --- | --- | --- | --- |
| Acoustic Radiation | 0.472 | 0.842 | 0.063 |
| Anterior Thalamic Radiation | 0.669 | 0.986 | -0.038 |
| Cingulate gyrus – part of cingulum | 0.787 | 0.986 | -0.025 |
| Parahippocampal part of cingulum | 0.308 | 0.842 | -0.089 |
| Corticospinal Tract | 0.360 | 0.842 | -0.088 |
| Inferior fronto-occipital fasciculus | 0.505 | 0.842 | -0.061 |
| Inferior longitudinal fasciculus | 0.488 | 0.842 | -0.062 |
| Medial Lemniscus | 0.460 | 0.842 | -0.064 |
| Posterior Thalamic Radiation | 0.410 | 0.842 | -0.073 |
| Superior Longitudinal fasciculus | 0.362 | 0.842 | 0.084 |
| Superior Thalamic Radiation | 0.960 | 0.986 | -0.004 |
| Uncinate Fasciculus | 0.943 | 0.986 | 0.006 |
| Forceps Major* | 0.100 | 0.842 | 0.013 |
| Forceps Minor | 0.981 | 0.986 | 0.000 |
| Middle Cerebellar Peduncle | 0.986 | 0.986 | -0.011 |

Table 95: Interaction effect between depression and Visions on MD values

### Voices

| Subset | P-value | FDR P-value | Β-coefficient |
| --- | --- | --- | --- |
| General MD | 0.654 | 0.872 | -0.003 |
| Association fibres | 0.557 | 0.872 | -0.005 |
| Thalamic fibres | 0.550 | 0.872 | -0.004 |
| Sensory fibres | 0.881 | 0.881 | -0.001 |

Table 96: Effect of 'Voices' on MD values of subsets white matter tracts

| Brain Structure | P-value | FDR P-Value | β-coefficient |
| --- | --- | --- | --- |
| Acoustic Radiation | 0.260 | 0.856 | -0.061 |
| Anterior Thalamic Radiation | 0.538 | 0.856 | 0.033 |
| Cingulate gyrus – part of cingulum | 0.877 | 0.940 | -0.009 |
| Parahippocampal part of cingulum | 0.628 | 0.856 | 0.026 |
| Corticospinal Tract | 0.185 | 0.856 | -0.079 |
| Inferior fronto-occipital fasciculus | 0.426 | 0.856 | 0.045 |
| Inferior longitudinal fasciculus | 0.386 | 0.856 | 0.048 |
| Medial Lemniscus | 0.824 | 0.940 | -0.012 |
| Posterior Thalamic Radiation | 0.287 | 0.856 | 0.058 |
| Superior Longitudinal fasciculus | 0.487 | 0.856 | 0.040 |
| Superior Thalamic Radiation | 0.842 | 0.940 | -0.011 |
| Uncinate Fasciculus | 0.586 | 0.856 | 0.028 |
| Forceps Major* | 0.603 | 0.856 | 0.004 |
| Forceps Minor | 0.948 | 0.948 | -0.001 |
| Middle Cerebellar Peduncle | 0.092 | 0.856 | 0.014 |

Table 97: Effect of Voices on MD values of white matter tracts

| Brain Structure | P-value | FDR P-Value | β-coefficient |
| --- | --- | --- | --- |
| Acoustic Radiation | 0.260 | 0.837 | -0.115 |
| Anterior Thalamic Radiation | 0.538 | 0.837 | 0.097 |
| Cingulate gyrus – part of cingulum | 0.877 | 0.877 | 0.001 |
| Parahippocampal part of cingulum | 0.628 | 0.837 | -0.078 |
| Corticospinal Tract | 0.185 | 0.837 | -0.053 |
| Inferior fronto-occipital fasciculus | 0.426 | 0.837 | 0.115 |
| Inferior longitudinal fasciculus | 0.386 | 0.837 | 0.093 |
| Medial Lemniscus | 0.824 | 0.877 | -0.111 |
| Posterior Thalamic Radiation | 0.287 | 0.837 | 0.158 |
| Superior Longitudinal fasciculus | 0.487 | 0.837 | 0.022 |
| Superior Thalamic Radiation | 0.842 | 0.877 | 0.071 |
| Uncinate Fasciculus | 0.586 | 0.837 | 0.024 |

Table 98: Interaction effect between hemisphere and Voices on MD values

| Brain Structure | P-value | FDR P-Value | β-coefficient |
| --- | --- | --- | --- |
| Acoustic Radiation | 0.862 | 0.920 | -0.019 |
| Anterior Thalamic Radiation | 0.739 | 0.907 | 0.036 |
| Cingulate gyrus – part of cingulum | 0.500 | 0.907 | -0.078 |
| Parahippocampal part of cingulum | 0.547 | 0.907 | -0.065 |
| Corticospinal Tract | 0.673 | 0.907 | -0.050 |
| Inferior fronto-occipital fasciculus | 0.920 | 0.920 | 0.012 |
| Inferior longitudinal fasciculus | 0.692 | 0.907 | 0.044 |
| Medial Lemniscus | 0.514 | 0.907 | 0.071 |
| Posterior Thalamic Radiation | 0.320 | 0.907 | 0.109 |
| Superior Longitudinal fasciculus | 0.657 | 0.907 | -0.051 |
| Superior Thalamic Radiation | 0.266 | 0.907 | -0.120 |
| Uncinate Fasciculus | 0.756 | 0.907 | -0.032 |

Table 99: Interaction effect between sex and Visions on MD values

| Brain Structure | P-value | FDR P-Value | β-coefficient |
| --- | --- | --- | --- |
| Acoustic Radiation | 0.290 | 0.947 | 0.116 |
| Anterior Thalamic Radiation | 0.426 | 0.947 | 0.087 |
| Cingulate gyrus – part of cingulum | 0.744 | 0.947 | 0.038 |
| Parahippocampal part of cingulum | 0.947 | 0.947 | 0.007 |
| Corticospinal Tract | 0.756 | 0.947 | 0.037 |
| Inferior fronto-occipital fasciculus | 0.764 | 0.947 | 0.034 |
| Inferior longitudinal fasciculus | 0.823 | 0.947 | 0.025 |
| Medial Lemniscus | 0.512 | 0.947 | -0.071 |
| Posterior Thalamic Radiation | 0.893 | 0.947 | -0.015 |
| Superior Longitudinal fasciculus | 0.132 | 0.817 | 0.173 |
| Superior Thalamic Radiation | 0.163 | 0.817 | 0.150 |
| Uncinate Fasciculus | 0.772 | 0.947 | 0.030 |
| Forceps Major* | 0.687 | 0.947 | -0.005 |
| Forceps Minor | 0.934 | 0.947 | 0.001 |
| Middle Cerebellar Peduncle | 0.152 | 0.817 | 0.008 |

Table 100: Interaction effect between depression and Voices on MD values

### Communications

| Subset | P-value | FDR P-value | Β-coefficient |
| --- | --- | --- | --- |
| General MD | 0.628 | 0.876 | -0.004 |
| Association fibres | 0.476 | 0.876 | -0.006 |
| Thalamic fibres | 0.742 | 0.876 | -0.002 |
| Sensory fibres | 0.876 | 0.876 | -0.001 |

Table 101: Effect of 'Communications' on MD values of subsets white matter tracts

| Brain Structure | P-value | FDR P-Value | β-coefficient |
| --- | --- | --- | --- |
| Acoustic Radiation | 0.801 | 0.980 | -0.022 |
| Anterior Thalamic Radiation | 0.980 | 0.980 | 0.002 |
| Cingulate gyrus – part of cingulum | 0.181 | 0.980 | 0.125 |
| Parahippocampal part of cingulum | 0.273 | 0.980 | 0.096 |
| Corticospinal Tract | 0.551 | 0.980 | -0.058 |
| Inferior fronto-occipital fasciculus | 0.930 | 0.980 | 0.008 |
| Inferior longitudinal fasciculus | 0.947 | 0.980 | -0.006 |
| Medial Lemniscus | 0.689 | 0.980 | -0.035 |
| Posterior Thalamic Radiation | 0.817 | 0.980 | 0.021 |
| Superior Longitudinal fasciculus | 0.661 | 0.980 | -0.041 |
| Superior Thalamic Radiation | 0.785 | 0.980 | -0.024 |
| Uncinate Fasciculus | 0.784 | 0.980 | -0.023 |
| Forceps Major* | 0.665 | 0.980 | 0.003 |
| Forceps Minor | 0.754 | 0.980 | -0.002 |
| Middle Cerebellar Peduncle | 0.647 | 0.980 | 0.004 |

Table 102: Effect of Communications on MD values of white matter tracts

| Brain Structure | P-value | FDR P-Value | β-coefficient |
| --- | --- | --- | --- |
| Acoustic Radiation | 0.801 | 0.980 | -0.001 |
| Anterior Thalamic Radiation | 0.980 | 0.980 | 0.024 |
| Cingulate gyrus – part of cingulum | 0.181 | 0.980 | 0.144 |
| Parahippocampal part of cingulum | 0.273 | 0.980 | -0.324 |
| Corticospinal Tract | 0.551 | 0.980 | -0.063 |
| Inferior fronto-occipital fasciculus | 0.930 | 0.980 | 0.008 |
| Inferior longitudinal fasciculus | 0.947 | 0.980 | -0.035 |
| Medial Lemniscus | 0.689 | 0.980 | -0.093 |
| Posterior Thalamic Radiation | 0.817 | 0.980 | -0.048 |
| Superior Longitudinal fasciculus | 0.661 | 0.980 | -0.002 |
| Superior Thalamic Radiation | 0.785 | 0.980 | 0.046 |
| Uncinate Fasciculus | 0.784 | 0.980 | 0.031 |

Table 103: Interaction effect between hemisphere and Communications on MD values

| Brain Structure | P-value | FDR P-Value | β-coefficient |
| --- | --- | --- | --- |
| Acoustic Radiation | 0.790 | 0.918 | 0.047 |
| Anterior Thalamic Radiation | 0.394 | 0.849 | -0.151 |
| Cingulate gyrus – part of cingulum | 0.451 | 0.849 | -0.141 |
| Parahippocampal part of cingulum | 0.392 | 0.849 | 0.150 |
| Corticospinal Tract | 0.460 | 0.849 | -0.143 |
| Inferior fronto-occipital fasciculus | 0.618 | 0.849 | -0.093 |
| Inferior longitudinal fasciculus | 0.637 | 0.849 | -0.086 |
| Medial Lemniscus | 0.930 | 0.930 | 0.015 |
| Posterior Thalamic Radiation | 0.841 | 0.918 | -0.036 |
| Superior Longitudinal fasciculus | 0.607 | 0.849 | -0.096 |
| Superior Thalamic Radiation | 0.288 | 0.849 | -0.186 |
| Uncinate Fasciculus | 0.331 | 0.849 | -0.162 |

Table 104: Interaction effect between sex and Communications on MD values

| Brain Structure | P-value | FDR P-Value | β-coefficient |
| --- | --- | --- | --- |
| Acoustic Radiation | 0.430 | 0.717 | -0.141 |
| Anterior Thalamic Radiation | 0.229 | 0.717 | -0.215 |
| Cingulate gyrus – part of cingulum | 0.395 | 0.717 | -0.160 |
| Parahippocampal part of cingulum | 0.248 | 0.717 | 0.204 |
| Corticospinal Tract | 0.035 | 0.525 | -0.410 |
| Inferior fronto-occipital fasciculus | 0.341 | 0.717 | -0.178 |
| Inferior longitudinal fasciculus | 0.380 | 0.717 | -0.161 |
| Medial Lemniscus | 0.496 | 0.732 | -0.120 |
| Posterior Thalamic Radiation | 0.826 | 0.826 | 0.039 |
| Superior Longitudinal fasciculus | 0.537 | 0.732 | -0.116 |
| Superior Thalamic Radiation | 0.396 | 0.717 | -0.149 |
| Uncinate Fasciculus | 0.397 | 0.717 | -0.142 |
| Forceps Major* | 0.720 | 0.826 | 0.002 |
| Forceps Minor | 0.743 | 0.826 | 0.006 |
| Middle Cerebellar Peduncle | 0.771 | 0.826 | 0.004 |

Table 105: Interaction effect between depression and Communications on MD values

### Conspiracies

| Subset | P-value | FDR P-value | Β-coefficient |
| --- | --- | --- | --- |
| General MD | 0.183 | 0.365 | -0.010 |
| Association fibres | 0.067 | 0.268 | -0.014 |
| Thalamic fibres | 0.434 | 0.579 | -0.006 |
| Sensory fibres | 0.986 | 0.986 | 0.000 |

Table 106: Effect of 'Conspiracies' on MD values of subsets white matter tracts

| Brain Structure | P-value | FDR P-Value | β-coefficient |
| --- | --- | --- | --- |
| Acoustic Radiation | 0.416 | 0.626 | 0.068 |
| Anterior Thalamic Radiation | 0.501 | 0.626 | 0.056 |
| Cingulate gyrus – part of cingulum | 0.258 | 0.626 | 0.100 |
| Parahippocampal part of cingulum | 0.035 | 0.518 | 0.175 |
| Corticospinal Tract | 0.608 | 0.702 | 0.047 |
| Inferior fronto-occipital fasciculus | 0.413 | 0.626 | 0.072 |
| Inferior longitudinal fasciculus | 0.268 | 0.626 | 0.095 |
| Medial Lemniscus | 0.487 | 0.626 | -0.058 |
| Posterior Thalamic Radiation | 0.458 | 0.626 | 0.062 |
| Superior Longitudinal fasciculus | 0.286 | 0.626 | 0.094 |
| Superior Thalamic Radiation | 0.443 | 0.626 | 0.063 |
| Uncinate Fasciculus | 0.209 | 0.626 | 0.099 |
| Forceps Major* | 0.709 | 0.760 | 0.003 |
| Forceps Minor | 0.366 | 0.626 | 0.007 |
| Middle Cerebellar Peduncle | 0.769 | 0.769 | -0.002 |

Table 107: Effect of Conspiracies on MD values of white matter tracts

| Brain Structure | P-value | FDR P-Value | β-coefficient |
| --- | --- | --- | --- |
| Acoustic Radiation | 0.416 | 0.546 | 0.094 |
| Anterior Thalamic Radiation | 0.501 | 0.546 | 0.125 |
| Cingulate gyrus – part of cingulum | 0.258 | 0.546 | 0.088 |
| Parahippocampal part of cingulum | 0.035 | 0.414 | 0.446 |
| Corticospinal Tract | 0.608 | 0.608 | -0.006 |
| Inferior fronto-occipital fasciculus | 0.413 | 0.546 | 0.025 |
| Inferior longitudinal fasciculus | 0.268 | 0.546 | 0.052 |
| Medial Lemniscus | 0.487 | 0.546 | -0.236 |
| Posterior Thalamic Radiation | 0.458 | 0.546 | -0.071 |
| Superior Longitudinal fasciculus | 0.286 | 0.546 | 0.154 |
| Superior Thalamic Radiation | 0.443 | 0.546 | 0.144 |
| Uncinate Fasciculus | 0.209 | 0.546 | 0.043 |

Table 108: Interaction effect between hemisphere and Conspiracies on MD values

| Brain Structure | P-value | FDR P-Value | β-coefficient |
| --- | --- | --- | --- |
| Acoustic Radiation | 0.757 | 0.757 | 0.052 |
| Anterior Thalamic Radiation | 0.562 | 0.741 | 0.098 |
| Cingulate gyrus – part of cingulum | 0.397 | 0.741 | 0.150 |
| Parahippocampal part of cingulum | 0.552 | 0.741 | 0.099 |
| Corticospinal Tract | 0.679 | 0.741 | 0.076 |
| Inferior fronto-occipital fasciculus | 0.463 | 0.741 | 0.129 |
| Inferior longitudinal fasciculus | 0.309 | 0.741 | 0.175 |
| Medial Lemniscus | 0.663 | 0.741 | 0.073 |
| Posterior Thalamic Radiation | 0.667 | 0.741 | 0.073 |
| Superior Longitudinal fasciculus | 0.346 | 0.741 | 0.167 |
| Superior Thalamic Radiation | 0.620 | 0.741 | 0.082 |
| Uncinate Fasciculus | 0.392 | 0.741 | 0.136 |

Table 109: Interaction effect between sex and Conspiracies on MD values

| Brain Structure | P-value | FDR P-Value | β-coefficient |
| --- | --- | --- | --- |
| Acoustic Radiation | 0.223 | 0.371 | -0.207 |
| Anterior Thalamic Radiation | 0.060 | 0.197 | -0.320 |
| Cingulate gyrus – part of cingulum | 0.314 | 0.471 | -0.180 |
| Parahippocampal part of cingulum | 0.110 | 0.236 | -0.269 |
| Corticospinal Tract | 0.140 | 0.262 | -0.273 |
| Inferior fronto-occipital fasciculus | 0.025 | 0.197 | -0.399 |
| Inferior longitudinal fasciculus | 0.048 | 0.197 | -0.344 |
| Medial Lemniscus | 0.628 | 0.673 | -0.082 |
| Posterior Thalamic Radiation | 0.034 | 0.197 | -0.361 |
| Superior Longitudinal fasciculus | 0.079 | 0.197 | -0.314 |
| Superior Thalamic Radiation | 0.074 | 0.197 | -0.299 |
| Uncinate Fasciculus | 0.623 | 0.673 | -0.079 |
| Forceps Major* | 0.806 | 0.806 | 0.012 |
| Forceps Minor | 0.370 | 0.505 | 0.022 |
| Middle Cerebellar Peduncle | 0.578 | 0.673 | -0.012 |

Table 110: Interaction effect between depression and Conspiracies on MD values

# References

1. Karla, L. M. *et al.* Multimodal population brain imaging in the UK Biobank prospective epidemiological study. *Nat. Neurosci.* **19**, (2016).

2. Alfaro-Almagro, F. *et al.* Image processing and Quality Control for the first 10,000 brain imaging datasets from UK Biobank. *Neuroimage* **166**, 400–424 (2018).

3. Zhang, Y., Brady, M. & Smith, S. Segmentation of brain MR images through a hidden Markov random field model and the expectation-maximization algorithm. *IEEE Trans. Med. Imaging* **20**, 45–57 (2001).

4. Patenaude, B., Smith, S. M., Kennedy, D. N. & Jenkinson, M. A Bayesian model of shape and appearance for subcortical brain segmentation. *Neuroimage* **56**, 907–922 (2011).

5. Neilson, E. *et al.* Impact of polygenic risk for Schizophrenia on cortical structure in UK Biobank. *Biol. Psychiatry* (2019).

6. Cox, S. R. *et al.* Ageing and brain white matter structure in 3,513 UK Biobank participants. *Nat. Commun.* **7**, (2016).

7. Shen, X. *et al.* Subcortical volume and white matter integrity abnormalities in major depressive disorder: findings from UK Biobank imaging data. *Sci Rep* **7**, 5547 (2017).

8. Barbu, M. C. *et al.* Association of Whole-Genome and NETRIN1 Signaling Pathway-Derived Polygenic Risk Scores for Major Depressive Disorder and White Matter Microstructure in the UK Biobank. *Biol. psychiatry. Cogn. Neurosci. neuroimaging* **4**, (2019).

9. Benjamini, Y. & Hochberg, Y. Controlling the False Discovery Rate: A Practical and Powerful Approach to Multiple Testing. *J. R. Stat. Soc. Ser. B* **57**, 289–300 (1995).

10. Linscott, R. & van Os, J. An updated and conservative systematic review and meta-analysis of epidemiological evidence on psychotic experiences in children and adults: on the pathway from proneness to persistence to dimensional expression across mental disorders. *Psychol. Med.* **43**, 1133–1149 (2013).

11. Desikan, R. S. *et al.* An automated labeling system for subdividing the human cerebral cortex on MRI scans into gyral based regions of interest. *Neuroimage* **31**, 968–980 (2006).
